# Designing optimal perturbation inputs for system identification in neuroscience

**DOI:** 10.7554/eLife.110030

**Published:** 2026-10-13

**Authors:** Mikito Ogino, Daiki Sekizawa, Jun Kitazono, Masafumi Oizumi

**Affiliations:** 1 Graduate School of Arts and Sciences, The University of Tokyo Meguro-ku Japan; 2 RIKEN Center for Brain Science, RIKEN Wako Japan; 3 Graduate School of Data Science, Yokohama City University Yokohama Japan; University College London United Kingdom; FORTH Institute of Molecular Biology and Biotechnology Greece

**Keywords:** brain stimulation, control theory, linear dynamical systems, resonance, system identification, tACS, None

## Abstract

Investigating the dynamics of neural networks, which are governed by connectivity between neurons, is a fundamental challenge in neuroscience. Because passive (spontaneous) activity provides only limited information for estimating connectivity, perturbation-based approaches are widely applied in neuroscience as they can evoke underlying hidden dynamics. However, the characteristics of such perturbations have typically been designed based on empirical or biological intuition. To enable more accurate estimation of connectivity, we propose a data-driven and theoretically grounded framework for optimally designing perturbation inputs, based on formulating the neural model as a control system. The core theoretical insight underlying our approach is that neural signals observed in the passive state lack sufficient latent information, which leads to failures in the system identification. Perturbations reveal these hidden dynamics and lead to improved estimation. Guided by these insights, we derive a theoretical basis for optimizing perturbation inputs that minimize estimation errors in neural system identification. Building upon this, we further explore the relationship of this theory with stimulation patterns commonly used in neuroscience, such as frequency, impulse, and step inputs. We demonstrate the effectiveness of this framework for neuroscience through simulations grounded in experimental paradigms, such as neural state classification and optimal control of neural states. Our theoretical analysis, together with multiple simulations, consistently shows that perturbations designed according to our framework achieve substantially more accurate system identification compared to the conventional, intuition-based inputs. This study provides a theoretical foundation for designing perturbation inputs to achieve accurate estimation of neural dynamics. This, in turn, enables reliable discrimination of neural states such as levels of consciousness and pathological conditions, and facilitates precise control of their transitions toward recovery from abnormal states.

## Introduction

**Figure 1.**
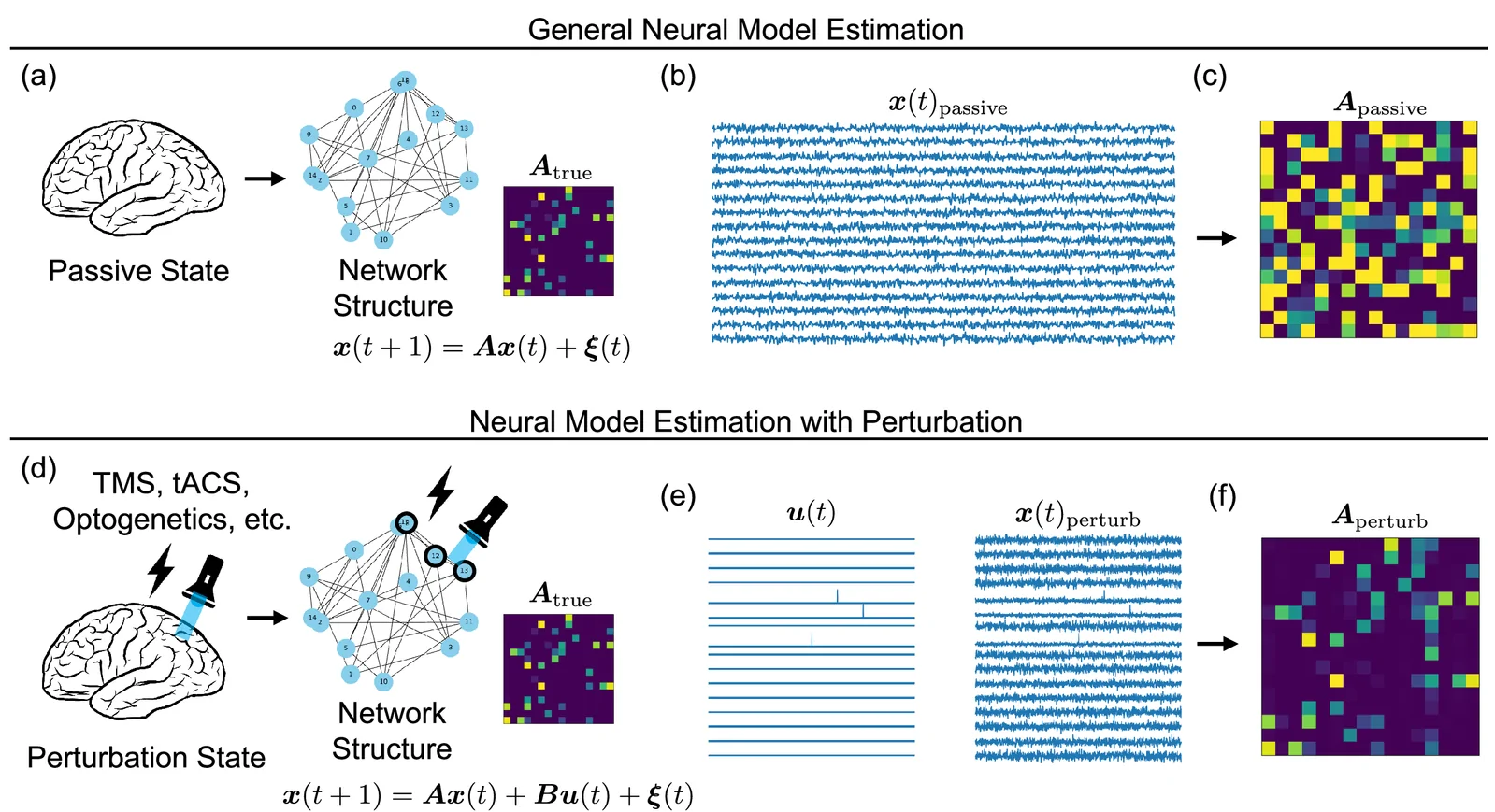
Passive and perturbation states and the resulting neural dynamics models. (**a**) Passive state of the neural network and the corresponding ground-truth model. (**b**) Recorded neural activities without perturbation. (**c**) Estimated model obtained without perturbation. (**d**) Perturbation state of the neural network and corresponding ground-truth model. (**e**) Recorded neural activity with perturbation. (**f**) Estimated model obtained with perturbation.

Much recent interest has focused on how interactions between individual neurons and neuronal populations support cognitive functions and behavior, and on how the disruption of these interactions contributes to various neurological and psychiatric disorders (Waites et al., 2006; Friston, 2011; Boly et al., 2012; Sporns, 2014; Petersen and Sporns, 2015). This interaction—called neuronal connectivity (Sporns, 2016; Seguin et al., 2023)—is often represented as a network structure or a connectivity matrix. In particular, a commonly studied aspect is functional connectivity, which captures the temporal dependencies between neural activities (Stevens, 2009; Honey et al., 2009; Rubinov and Sporns, 2010; Friston et al., 2013). The functional connectivity is estimated through recording techniques, such as neural spike recording techniques (Siegle et al., 2021; Yan and Murphy, 2024), electrocorticography (ECoG) (Park et al., 2016; Kucyi et al., 2018), functional magnetic resonance imaging (fMRI) (Greicius et al., 2007; Rocchi et al., 2022) and electroencephalography (EEG) (Nentwich et al., 2020; Bogéa Ribeiro and da Silva Filho, 2023). While various methods have been proposed and used to estimate these connections (see for example Friston, 2011; Reid et al., 2019 for a comprehensive review), one typical method is based on dynamic modeling, among which is a simple but widely used linear auto-regressive model (Figure 1a; Goebel et al., 2003; Ding et al., 2006; Ting et al., 2015; Seth et al., 2015; Cekic et al., 2018). Connectivity is statistically estimated from the time series data of neural activity (Figure 1b) by fitting the model parameters (Figure 1c). A neural connectivity matrix helps visualize the connections between different brain regions, and provides a detailed map of functional interactions within the brain. By comparing connectivity matrices across individuals or groups, researchers can deepen their understanding of neuronal architectures and communication across various disciplines (Stevens, 2009; Ruiz et al., 2014; Bassett and Sporns, 2017; Lee et al., 2020; Bergmann et al., 2021; Siddiqi et al., 2022).

**Figure 2.**
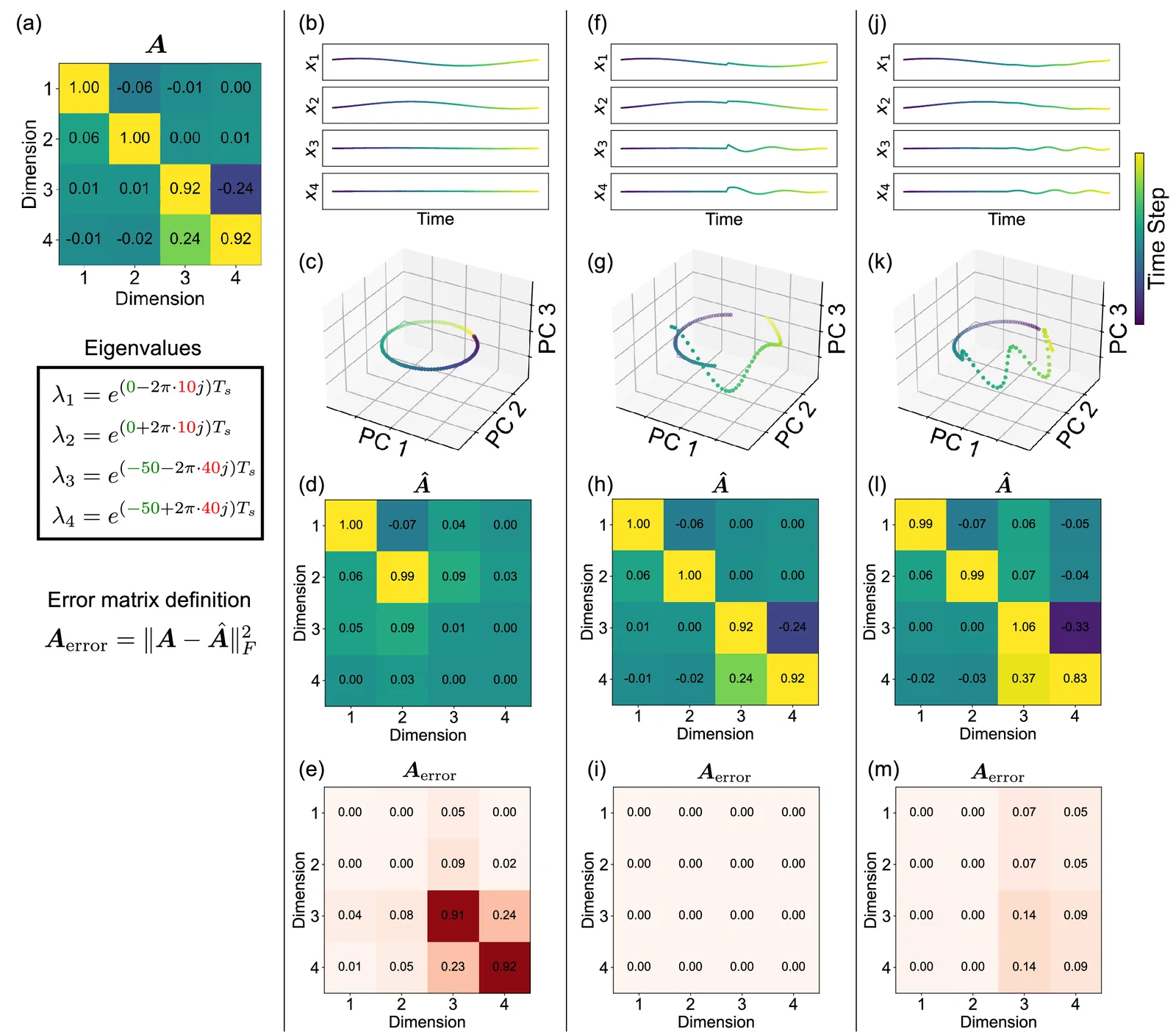
Visualization of continuous-time system dynamics by perturbations. (**a**) System matrix $\boldsymbol {A}$ representing the dynamics and its eigenvalues. (**b**) Temporal changes in the state variables of the passive state without perturbation. (**c**) Time-varying trajectories of each PCA component. The color gradient indicates temporal progression. (**d**) Estimated connectivity matrix in the passive condition. (**e**) Error matrix for the passive-state estimation. (**f–i**) Temporal changes and estimation results in response to an impulse input. (**j–m**) Temporal changes and estimation results in response to a sinusoidal input.

A fundamental problem with passive observation is that it fails to reveal hidden dynamics, which leads to an invalid estimate of the corresponding parts of the model. This limitation stems from the attenuation of latent dynamical modes, such as transient or damped components. When neural activity is modeled as a linear dynamical system, as shown in Figure 1a, the true connectivity matrix governs the generation of time-series signals (Figure 1b). Passive observation records these spontaneous neural activities within an arbitrarily predefined temporal window and attempts to estimate the connectivity matrix using parameter estimation techniques. However, the resulting matrix $\boldsymbol {A}_{\mathrm{passive}}$ deviates significantly from the true model (Figure 1c), because some modes quickly decay and vanish from the observable data (see also Figure 2 for an intuitive explanation). Consequently, relying solely on passive recordings leads to misinterpretations of the neural system—such as overlooking fast transient dynamics or underestimating connectivity strength—which, in turn, may result in inaccurate conclusions in both basic neuroscience and clinical contexts.

To overcome these limitations, the application of perturbations has emerged as a powerful approach for improving the estimation of connectivity in neuroscience (Bergmann et al., 2021; Casali et al., 2013; Stepaniants et al., 2020; Lepperød et al., 2023). Its effectiveness lies in its ability to actively manipulate the neural system, rather than merely observing it, and to thereby uncover dynamic and causal interactions that are otherwise hidden (Stepaniants et al., 2020). Recent studies using stimulation techniques, such as optogenetics (Lepperød et al., 2023; Steinberg and Janak, 2013) and transcranial magnetic stimulation (TMS) Bergmann et al., 2021; Hallett et al., 2017; Tik et al., 2023 have demonstrated that these interventions can significantly improve the detection of causal relationships within the neural system. Such approaches have been applied to the study of cognitive processes and states of consciousness (Bergmann et al., 2021; Casali et al., 2013). These studies underscore the value of perturbation inputs in driving state transitions in the neural system, thereby enabling more accurate and reliable connectivity estimation.

However, the parameters of perturbation protocols, such as the location, intensity, and shape of stimulation, have often been determined based on anatomical and physiological insights and on empirically established techniques (Bergmann et al., 2021; Massimini et al., 2007; George, 2019; Bernal-Casas et al., 2017; Grimm et al., 2024). While these approaches have provided practical utility, they may not optimally exploit the underlying neural dynamics or maximize the informativeness of the perturbation. To overcome these limitations and enhance the reliability of connectivity inference, there is a pressing need to design data-driven and theoretically grounded perturbations.

In this paper, we propose a framework for designing the optimal perturbation input through control theory in neuroscience. We interpret neural dynamics as a control system (Gu et al., 2015; Deng et al., 2022; Kawakita et al., 2022; Kamiya et al., 2023; Moradi Amani et al., 2024; Shikauchi et al., 2025), and treat external perturbations as control inputs to design properties of neural stimulation (Figure 1d). If the optimal perturbation input can be systematically designed, it becomes possible to steer the neural system toward states that are maximally informative (Figure 1e), thereby enhancing the accuracy of the inferred connectivity (Figure 1f). While recent studies have begun to develop algorithmic approaches to active stimulation design for specific experimental platforms (Minai et al., 2024; Wagenmaker et al., 2024), a general theoretical framework for why and which perturbation inputs are effective has yet to be established. We first describe how to formulate neural dynamics as a control system and how to estimate the model parameters from observed data. Building upon this formulation, we derive a theoretical basis that enables us to design the optimal perturbation inputs for the neural system identification. We demonstrate the validity and utility of this theoretical basis by exploring its implications for optimizing parameters of common neurostimulation techniques and by applying it to practical examples, including neural state classification (Casali et al., 2013; Hallett et al., 2017; Shikauchi et al., 2025; Angulo-Sherman et al., 2017) and control of neural states (Gu et al., 2015; Kawakita et al., 2022; Kamiya et al., 2023; Karrer et al., 2020). In these demonstrations, we define concrete problems and apply the theory to validate its practical utility.

Our research offers a comprehensive framework for system identification with perturbation in neuroscience, and paves the way for more precise and effective analysis of neural dynamics. By providing clear guidelines on the design and application of perturbations, this framework serves as a practical reference for experimental settings, helping researchers determine the most effective stimulation parameters for their studies.

### Background

To evaluate how external perturbations enhance the accuracy of system identification, this section reviews the estimation formulations for linear dynamical systems, comparing the cases with and without perturbation inputs.

### System identification with external perturbation

This section reviews a well-established framework for system identification of linear dynamical systems with external perturbations. Researchers have modeled brain dynamics as a discrete-time linear dynamical system with external inputs and stochastic system noise (Gu et al., 2015; Deng et al., 2022; Betzel et al., 2016; Kim et al., 2018; Braun et al., 2021). We assume that the brain state evolves according to the following linear dynamics:

(1) $$\displaystyle \boldsymbol{x}(t+1) = \boldsymbol{A}\boldsymbol{x}(t) + \boldsymbol{B}\boldsymbol{u}(t) + \boldsymbol{\xi}(t),$$

where $\boldsymbol {x}(t) \in \mathbb{R}^{n}$ denotes the neural state vector at time *t*, such as the activity of neurons, populations, or brain regions, and *n* represents its dimension. The connectivity matrix $\boldsymbol {A}\in \mathbb{R}^{n \times n}$ characterizes intrinsic interactions reflecting functional connectivity. The input matrix $\boldsymbol {B}\in \mathbb{R}^{n \times m}$ specifies how external inputs $\boldsymbol {u}(t) \in \mathbb{R}^{m}$—including sensory stimuli or interventions, such as TMS—affect the system, where m represents the number of perturbation channels. The input $\boldsymbol {u}(t)$ is freely designed and known to the experimenter. Finally, $\boldsymbol {\xi }(t) \in \mathbb{R}^{n}$ represents stochastic fluctuations (e.g. synaptic noise or unobserved inputs), modeled as i.i.d. zero-mean Gaussian noise across time (i.e. delta-correlated in time) with covariance matrix $\boldsymbol {\Sigma }_{\xi } = \mathbb{E}[\boldsymbol {\xi }(t)\boldsymbol {\xi }(t)^{\!\top }] \succ 0$. We remark that neural activities measured at the macroscopic level, such as functional magnetic resonance imaging (fMRI) or intracranial electroencephalography (iEEG) have been experimentally and theoretically validated to follow approximately linear dynamical systems in Ahmed and Nozari, 2022; Nozari et al., 2024.

Here, we assume the input matrix ***B*** is known, and we are only estimating the connectivity matrix ***A*** from the time series data of ***x*** and ***u***. We construct the data matrices as

(2) $$\displaystyle \mathbf{X}=[\boldsymbol{x}(0),\boldsymbol{x}(1),\ldots,\boldsymbol{x}(T-1)],\quad \mathbf{Y}=[\boldsymbol{x}(1),\ldots,\boldsymbol{x}(T)],\quad \mathbf{U}=[\boldsymbol{u}(0),\ldots,\boldsymbol{u}(T-1)],$$

where ***T*** represents the length of the time series data. Using these matrices, the parameter matrix ***A*** can be estimated by minimizing the reconstruction error in the sense of ordinary least squares (OLS):

(3) $$\displaystyle \hat {\boldsymbol {A}} = \underset{\boldsymbol {A}}{\arg\min}\|\boldsymbol {Y}- (\boldsymbol {A}\boldsymbol {X}+ \boldsymbol {B}\boldsymbol {U})\|_{F}^{2}$$

(4) $$\displaystyle = (\boldsymbol {Y}- \boldsymbol {B}\boldsymbol {U}) \boldsymbol {X}^{\dagger },$$

where $\|\cdot \|_{F}$ represents the Frobenius norm and the symbol ^†^ denotes the pseudoinverse of a matrix. This manuscript derives a theoretical framework based on this formulation, which assumes linear dynamics with the input matrix ***B*** known. The relaxation of these assumptions—nonlinear state dynamics and the case of unknown ***B***—is addressed in Appendix 2.2 and Appendix 2.3.

### System identification without external perturbation

To compare the quality of system identification with and without perturbations, we also consider the passive case without external input. This corresponds to setting $\boldsymbol {u}(t) = \boldsymbol {0}$ in the formulation in the perturbed case, yielding the following dynamics:

(5) $$\displaystyle \boldsymbol {x}_{\mathrm{passive}}(t+1) = \boldsymbol {A}\boldsymbol {x}_{\mathrm{passive}}(t) + \boldsymbol {\xi }(t).$$

Here, ***A*** and $\boldsymbol {\xi }(t)$ are identical to those defined in Equation 1. Because this system evolves differently from the perturbed case, we denote its state trajectory as $\boldsymbol {x}_{\mathrm{passive}}(t)$ to explicitly distinguish it from the dynamics under perturbation.

Similarly, the parameter matrix ***A*** in the passive condition can be estimated by applying the ordinary least squares (OLS) method:

(6) $$\displaystyle \hat {\boldsymbol {A}}_{\mathrm{passive}}= \boldsymbol {Y}_{\mathrm{passive}}\boldsymbol {X}_{\mathrm{passive}}^{\dagger },$$

where

(7) $$\displaystyle \boldsymbol {X}_{\mathrm{passive}}= \begin{bmatrix} \boldsymbol {x}_{\mathrm{passive}}(0), \boldsymbol {x}_{\mathrm{passive}}(1), \dots , \boldsymbol {x}_{\mathrm{passive}}(T-1) \end{bmatrix} , \quad \boldsymbol {Y}_{\mathrm{passive}}= \begin{bmatrix} \boldsymbol {x}_{\mathrm{passive}}(1), \dots , \boldsymbol {x}_{\mathrm{passive}}(T) \end{bmatrix} .$$

The subscript ‘passive’ is used again to explicitly distinguish variables associated with the unperturbed dynamics from those obtained under external perturbations.

### Neural stimulation as control inputs

This section describes how commonly used neural stimulation techniques can be related to input signals in control theory. Their adjustable parameters vary depending on how the stimulation inputs are modulated.

Three non-invasive electrical stimulation methods illustrate how stimulation paradigms map onto basic control inputs. Transcranial magnetic stimulation (TMS) induces brief and transient perturbations via electromagnetic pulses (Momi et al., 2023), which are naturally represented as a sequence of impulse-like inputs, where the timing and intensity of each pulse are the primary controllable parameters. Transcranial direct current stimulation (tDCS) primarily modulates neural activity through approximately constant inputs (Tapsell et al., 2025), which can be viewed as a step-like signal whose main controllable parameter is the amplitude of the applied current. Transcranial alternating current stimulation (tACS) delivers oscillatory inputs (Grover et al., 2023), corresponding to sinusoidal signals characterized by amplitude, frequency, and phase. In control theory, impulse, step, and sinusoidal inputs are the basic components used to characterize system responses and dynamics (Ogata, 2010; Nise, 2020).

The control input framework extends beyond non-invasive techniques to invasive and optogenetic stimulation. Invasive electrical stimulation, including intracranial microstimulation and deep brain stimulation (DBS), enables direct delivery of electrical inputs to neural tissue (Lozano et al., 2019), providing flexible control over amplitude and timing through pulse trains or temporally structured waveforms. Optogenetic stimulation allows genetically targeted activation or inhibition of specific neurons using light (Deisseroth, 2011), providing fine-grained control over multiple input dimensions, including amplitude (light intensity), temporal pattern, and cell-type specificity. In particular, recent developments enable stimulation at the level of individual neurons with high temporal precision (Tong et al., 2023; LaFosse et al., 2023), allowing flexible construction of spatiotemporal input patterns.

These stimulation examples demonstrate that the theoretical framework developed in this paper connects to practical experimental settings. While a substantial gap remains between idealized control inputs in theory and experimentally realizable stimulation, the core principles established in the following sections provide a foundation that naturally extends to these practical stimulation paradigms.

## Results

We present a theoretical framework for determining the optimal perturbation inputs to enhance neural system identification. We begin by presenting an intuitive example in which the failure of passive estimation is demonstrated, and the benefit of perturbation inputs in re-exciting weakly observable dynamics becomes evident. Following this, we provide a guiding theoretical principle that estimation error of ***A*** is reduced by excitation of the state $\(\boldsymbol {x}\)$ by perturbation input ***u***. We then validate this theoretical foundation by applying it to canonical perturbation signals used in neuroscience—sinusoidal, impulse, and step inputs, as typically delivered by tACS, TMS, and tDCS, respectively—and derive analytical relationships between input parameters and estimation errors. Furthermore, we demonstrate the applicability of the proposed framework to practical problems in neuroscience, such as neural state classification and optimal control for neural state transitions. Finally, we outline a practical framework for designing optimal perturbation inputs to estimate neural dynamics.

### Typical failure of passive-state model estimation

We demonstrate why passive state observation, a common experimental condition in system neuroscience, fails to estimate the system model of neural dynamics. Although passive observation is widely employed due to its convenience and non-invasiveness, this practice has fundamental limitations: it inevitably overlooks causal and dynamical information that vanishes under spontaneous conditions but can be recovered through external perturbations, leading to degraded connectivity estimates. Such limitations lead to misinterpretations of the underlying neural functions when neural dynamics are modeled as a control system.

We estimate the connectivity matrix from simulated time series data generated by the true model. The true model, characterized by a connectivity matrix ***A***, is illustrated in Figure 2a. Neural data are generated based on the connectivity matrix, yielding the time series shown in Figure 2b. These data are not influenced by external input (passive state), and thus the oscillations of $x_{3}$ and $x_{4}$ gradually attenuate over time. This attenuation is further confirmed by principal component analysis (PCA), which reveals that the first and second principal components capture the 10 Hz dynamics, while the third principal component, associated with the 40 Hz mode, contributes negligibly to the total variance (Figure 2c). Consequently, the matrix estimated by OLS deviates substantially from the true connectivity matrix (Figure 2d), particularly showing errors in the lower-right components, as highlighted in Figure 2e. This failure arises because the true matrix ***A*** contains two distinct dynamical modes: one is a continuous oscillatory mode at 10 Hz, and the other is a damped mode at 40 Hz. The damped mode becomes unobservable in the time series once its contribution has attenuated and vanished. Passive recordings capture only superficial aspects of the connectivity and fail to estimate the true underlying neural dynamics.

The limitation of the passive state can be resolved by applying perturbation inputs. Figure 2f–i shows the application of an impulse input ***u*** to the system. This perturbation input primarily affects the nodes corresponding to $x_{3}$ and $x_{4}$, reintroducing variations in the third principal component direction. Estimating matrix ***A*** from this perturbed time-series data yields a more accurate estimate than in the passive case. Similarly, an improvement is observed when different types of input—a sinusoidal input, for example—are used instead of the impulse input, as shown in Figure 2j–m. These results illustrate that appropriate perturbation inputs can effectively re-excite weak dynamics, thereby enhancing system identification accuracy. This can be theoretically supported by the concept of persistent excitation (Green and Moore, 1986; Shimkin and Feuer, 1987; Jenkins et al., 2018; Lu and Cannon, 2023). According to this theory, a perturbation input is said to be persistently exciting if it causes the system’s state to sufficiently explore the state space over time. These insights underscore the importance of incorporating controlled stimulation in experimental design, particularly when accurate system identification is desired. A similar trend under nonlinear dynamics is shown in Appendix 2.2.

### Intuitive perturbation design informed by covariance eigenvalues

In this section, we show how perturbations reduce the estimation error of the system matrix ***A*** by exciting the state dynamics, thereby providing a theoretical basis for designing effective perturbation inputs. When the data length ***T*** is sufficiently large ($T \to \infty$), the estimation error of ***A*** asymptotically converges to the following expression (Hamilton, 1994):

(8) $$\displaystyle \begin{array}{ll}\mathbb{E}\bigl [\|\hat {\boldsymbol {A}}- \boldsymbol {A}\|_{F}^{2}\bigr ] \approx \frac{1}{T}\,{\rm tr}(\boldsymbol {\Sigma }_{\xi })\,{\rm tr} (\boldsymbol {\Sigma }_{\boldsymbol {X}}^{-1}).\end{array}$$

where $\boldsymbol {\Sigma }_{\boldsymbol {X}}= \frac{1}{T}(\boldsymbol {X}- \bar {\boldsymbol {X}})(\boldsymbol {X}- \bar {\boldsymbol {X}})^{\top }$ with $\bar {\boldsymbol {X}}= \frac{1}{T}\sum _{t=0}^{T-1}\boldsymbol {x}(t)$ represents the covariance matrix of the state vector *x*, and tr denotes the trace of a matrix. The detailed derivation of this equation is provided in Appendix 1.1. This equation indicates that the estimation error of ***A*** can be reduced either by increasing the measurement duration *T* or by minimizing the trace of the inverse covariance matrix $\boldsymbol {\Sigma }_{\boldsymbol {X}}^{-1}$, which depends on the perturbation input $\boldsymbol {u}(t)$. Intuitively, this relationship resembles a signal-to-noise ratio, where increasing the covariance of the state dynamics relative to the noise covariance leads to improved estimation accuracy.

The asymptotic expression in Equation 8 reveals that the estimation error of ***A*** depends inversely on the eigenvalues of the covariance matrix $\(\boldsymbol {\Sigma }_{\boldsymbol {X}}\)$. Expressing this relationship explicitly in terms of the eigenvalues yields

(9) $$\displaystyle  \begin{array}{ll}\mathbb{E}\bigl [\|\hat {\boldsymbol {A}}- \boldsymbol {A}\|_{F}^{2}\bigr ] \approx \frac{1}{T}\,{\rm tr} (\boldsymbol {\Sigma }_{\xi })\,\sum _{i=1}^{n} \frac{1}{\mu _{i}}.\end{array}$$

where $\mu _{i}$ denotes the *i*-th eigenvalue of $\boldsymbol {\Sigma }_{\boldsymbol {X}}$. This relationship indicates that small eigenvalues dominate the estimation error through their inverse contributions. Therefore, the perturbation input should be designed not only to increase the overall variance of the state *x*, but to enlarge the eigenvalues of $\boldsymbol {\Sigma }_{\boldsymbol {X}}$ more uniformly across all directions of the state space. In other words, exciting all dynamical modes of the system—rather than amplifying a limited subset—is essential for improving estimation accuracy.

**Figure 3.**
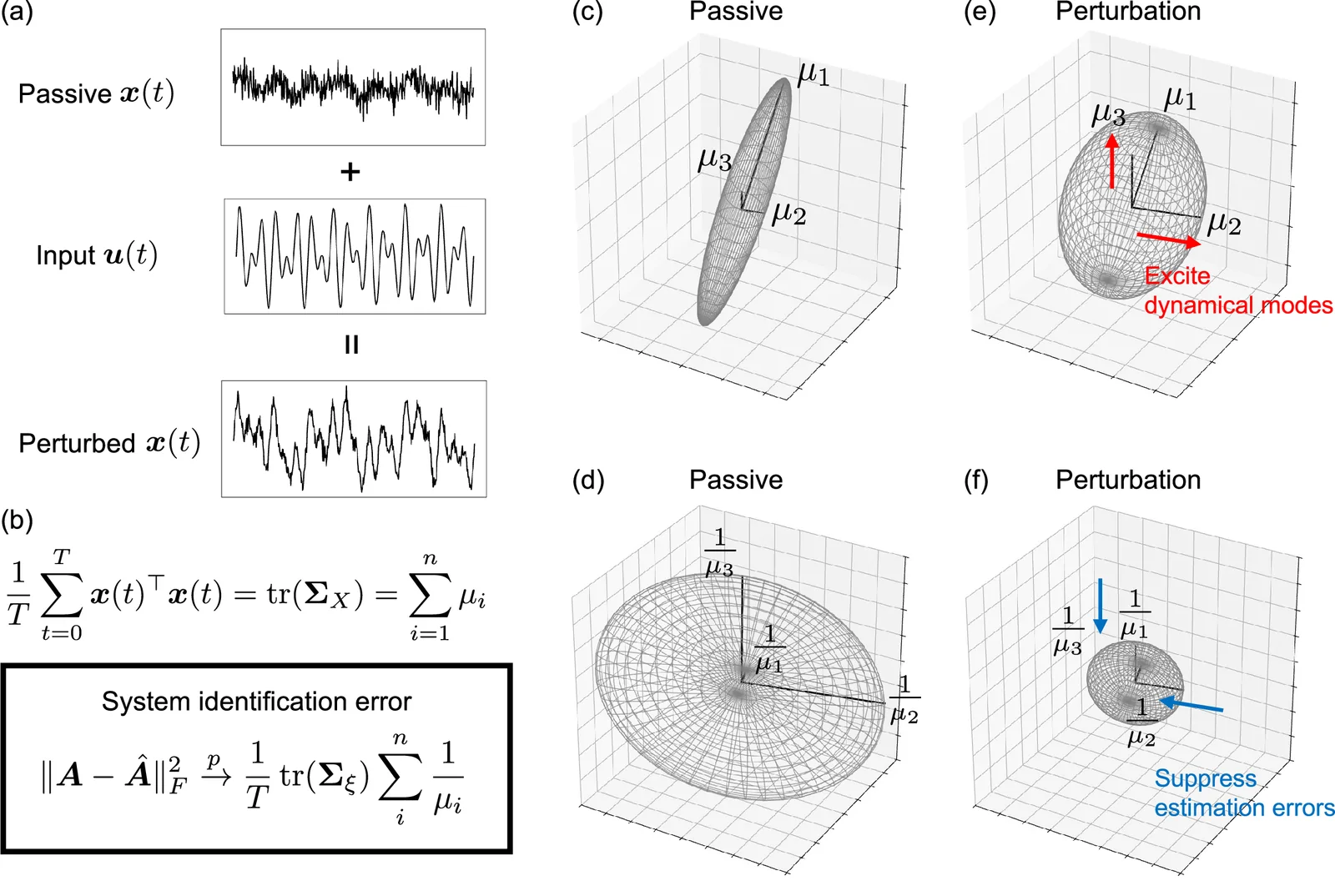
Effect of perturbation on the eigenvalues of the state covariance matrix. Ellipsoidal plots show the eigenvalues and reciprocal eigenvalues of the covariance matrix of the state trajectories $\boldsymbol {X}$, comparing passive dynamics and the case with perturbation. All ellipsoidal plots (**c**–**f**) are drawn along the first three eigenvector directions of $\boldsymbol {\Sigma }_{\boldsymbol {X}}$; panels (**c, e**) scale the axes by the eigenvalues $\mu _{i}$, while panels (**d, f**) scale them by the reciprocals $1/\mu _{i}$. (**a**) Examples of a passive signal, a perturbation input, and a perturbed signal. (**b**) Analytical relationship linking the eigenvalues of the state covariance matrix to system identification error. (**c**) Passive: The covariance matrix is dominated by a single eigenvalue direction. (**d**) Passive (reciprocal): A single dominant eigenvalue produces a widely spread reciprocal-space ellipsoid (**e**) Perturbation: Additional dynamical modes are excited, increasing the smaller eigenvalues. (**f**) Perturbation (reciprocal): Perturbations yield a more uniform reciprocal-space ellipsoid, reducing estimation error.

This theoretical insight can be illustrated intuitively in Figure 3. The figure visualizes ellipsoids constructed from the eigenvalues $\mu _{i}$ and their reciprocals $1/\mu _{i}$ of the covariance matrix $\boldsymbol {\Sigma }_{\boldsymbol {X}}$. The covariance matrices $\boldsymbol {\Sigma }_{\boldsymbol {X}}$ are calculated from the time series of the state vector *x* obtained under passive and perturbation conditions (Figure 3a). The length of each axis of the ellipsoid corresponds to the variance of the state along that direction, which is associated with the eigenvalue $\mu _{i}$. Its reciprocal counterpart $1/\mu _{i}$ represents the contribution to the estimation error (Figure 3b). As shown in Figure 3c, when the neural dynamics is dominated by a single eigenvalue $\mu _{1}$, the other eigenvalues $\mu _{2}$ and $\mu _{3}$ remain small, resulting in an elongated reciprocal ellipsoid (Figure 3d) and a large estimation error. When perturbation inputs that excite the directions associated with $\mu _{2}$ and $\mu _{3}$ are applied, the ellipsoid expands and becomes closer to a sphere (Figure 3e). As a result, the estimation error decreases in all directions, as illustrated in Figure 3f. This uniform enlargement of the eigenvalues provides a clear guideline: perturbations should be designed so that the variance becomes large in all directions, rather than being confined to specific modes.

### Theoretical formulation of sinusoidal inputs for an overall increase in eigenvalues

Based on the guideline presented in the previous section, we derive analytical expressions to investigate which frequency of perturbation input will efficiently excite the dynamical modes of a system. In neuroscience, such a perturbation is analogous to tACS. Since neural dynamics possess modal frequencies, there should exist perturbation input frequencies that resonate with these modes. Such resonance leads to an amplification of $\boldsymbol {x}(t)$, which generally results in larger eigenvalues $\mu _{i}$ of the covariance matrix $\boldsymbol {\Sigma }_{\boldsymbol {X}}$, reflecting increased variability along the corresponding modes. To clarify this relationship, we derive an analytical expression for $\boldsymbol {x}(t)$ to investigate how the input frequency affects its amplitude. The solution $\boldsymbol {x}(t)$ to the model in Equation 1 can be obtained by iterating the state transition matrix. Specifically,

(10) $$\displaystyle  \boldsymbol {x}(t) = \boldsymbol {A}^{t} \boldsymbol {x}(0) + \sum _{k=0}^{t-1}\boldsymbol {A}^{t-1-k}\boldsymbol {B}\boldsymbol {u}(k) + \sum _{k=0}^{t-1}\boldsymbol {A}^{t-1-k}\boldsymbol {\xi }(k)$$

We assume a cosine input $\boldsymbol {u}(k)=\sum _{l=1}^{L}\cos (\omega _{l}k)\boldsymbol {u}_{0}$, where $\omega _{l}$ are the angular frequencies. By substituting this into the second term, we can obtain the following equation by performing diagonalization of $\boldsymbol {A}$ and expressing the eigenvalues in polar form.

(11) $$\displaystyle \boldsymbol {x}(t) = \boldsymbol {x}_{\mathrm{passive}}(t)+\boldsymbol {x}_{\mathrm{diff}}(t),$$

(12) $$\displaystyle  \boldsymbol {x}_{\mathrm{passive}}(t) = \boldsymbol {A}^{t} \boldsymbol {x}(0) + \sum _{k=0}^{t-1}\boldsymbol {A}^{t-1-k}\boldsymbol {\xi }(k)$$

(13) $$\displaystyle  \boldsymbol {x}_{\mathrm{diff}}(t) = \sum _{l=1}^{L}\sum _{d=1}^{n}\mathrm{Re}\left [\frac{r_{d}^{t+1}e^{i\theta _d(t-1)}-r_{d}^{t}e^{i(\theta _dt-\omega _l)}-r_{d}e^{i(\omega _l t-\theta _d)}+e^{i\omega _l(t-1)}}{r_{d}^{2}-2r_{d}\cos (\theta _{d}-\omega _{l})+1}\boldsymbol {v}_{d} \boldsymbol {w}_{d}^{\top } \boldsymbol {B}\,\boldsymbol {u}_{0}\right ]$$

where each eigenvalue of $\boldsymbol {A}$ is written in polar form as $\lambda _{d} = r_{d} e^{i\theta _d}$, with $r_{d} = |\lambda _{d}|$ denoting the magnitude and $\theta _{d} = \arg (\lambda _{d})$ denoting the argument. $\boldsymbol {x}_{\mathrm{passive}}$ represents the state *x* that would be realized if no perturbation were applied, i.e., the state that would evolve according to Equation 5. The vector $\boldsymbol {v}_{d}$ denotes the right eigenvector of ***A*** associated with $\lambda _{d}$, and $\boldsymbol {w}_{d}^{\top }$ denotes the corresponding left eigenvector. The detailed derivation is provided in Appendix 1.2.1.

We focus on $\boldsymbol {x}_{\mathrm{diff}}(t)$ simply because it is the component directly driven by the control input. When the input frequencies $\omega _{l}$ coincide with the mode’s angular component $\theta _{d}$, a resonance-like amplification occurs in $\boldsymbol {x}_{\mathrm{diff}}(t)$—that is, the amplitude of $\boldsymbol {x}(t)$ increases markedly. Increasing the magnitude of $\boldsymbol {x}(t)$ through resonance naturally leads to an increase in the overall variance in $\boldsymbol {\Sigma }_{\boldsymbol {X}}$. This enhancement directly contributes to increasing all eigenvalues $\mu _{i}$ of the covariance matrix rather than leaving some unexcited. Therefore, the analysis of Equation 13 provides the necessary guidelines to target and amplify specific dynamical modes, suggesting that oscillatory inputs, such as tACS, should be designed with frequencies that correspond to the true dynamical mode frequencies.

### Theoretical formulation of impulse and step inputs

The eigenvalues of the covariance matrix can also be increased by manipulating the intensity of the perturbation input. In neuroscience, external perturbations, such as TMS and tDCS, can be modeled as impulse-like or step-like inputs to neural systems. The state vector ***x****(t)* for impulse inputs can be written as:

(14) $$\displaystyle  \boldsymbol {x}_{\mathrm{passive}}(t) = \boldsymbol {A}^{t}\boldsymbol {x}(0)+\sum _{k=0}^{t-1}\boldsymbol {A}^{t-1-k}\boldsymbol {\xi }(k),\quad \boldsymbol {x}_{\mathrm{diff}}(t) = \alpha \,\boldsymbol {A}^{t-1}\boldsymbol {B}\boldsymbol {u}_{0}.$$

For a step input, the state vector is described by,

(15) $$\displaystyle  \boldsymbol {x}_{\mathrm{passive}}(t) = \boldsymbol {A}^{t}\boldsymbol {x}(0)+\sum _{k=0}^{t-1}\boldsymbol {A}^{t-1-k}\boldsymbol {\xi }(k),\quad \boldsymbol {x}_{\mathrm{diff}}(t) = \beta \sum _{k=0}^{t-1}\boldsymbol {A}^{t-1-k}\boldsymbol {B}\boldsymbol {u}_{0}.$$

where *α* and *β* are the intensities of the impulse and step inputs. The detailed derivation is provided in Appendix 1.2.2. From these equations, it is clear that the contribution of the perturbation to the state covariance increases proportionally to the squares of the input strengths, $\alpha ^{2}$ and $\beta ^{2}$. However, estimation accuracy is not determined by input intensity alone. Equations 14; 15 show that the resulting dynamics $\boldsymbol {x}_{\mathrm{diff}}(t)$ are critically dependent on the term $\boldsymbol {B}\boldsymbol {u}_{0}$. This term represents how the input vector $\boldsymbol {u}_{0}$ (which defines the spatial pattern of the stimulation, i.e. which nodes are targeted) interacts with the system’s input matrix ***B***. Therefore, while increasing intensity (*α*, *β*) within experimental constraints is beneficial, the spatial pattern $\boldsymbol {u}_{0}$ is the key design parameter that determines which dynamical modes are excited. An improperly chosen $\boldsymbol {u}_{0}$ may excite only the modes already dominant in the passive state, failing to enlarge the smaller eigenvalues that are critical for system identification. The problem of how to design the optimal spatial pattern $\boldsymbol {u}_{0}$ to most effectively excite the weakly observable dynamics will be addressed in a later section.

### Demonstration of sinusoidal inputs

In this section, we demonstrate how to design the frequency of oscillatory input by analyzing the eigenvalues of the covariance matrix. Theoretical formulations indicate that tuning the frequency of oscillatory perturbation inputs is more complex compared to that of impulse and step inputs. We generate neural dynamics governed by the connectivity matrix ***A***, which is characterized by designated dynamical modes and compare its eigenvalues ${\lambda _i}$ with eigenvalues $\mu _{i}$ of the covariance matrix of the perturbed state vectors. The demonstration for impulse and step inputs can be found in Appendix 2.4.

**Figure 4.**
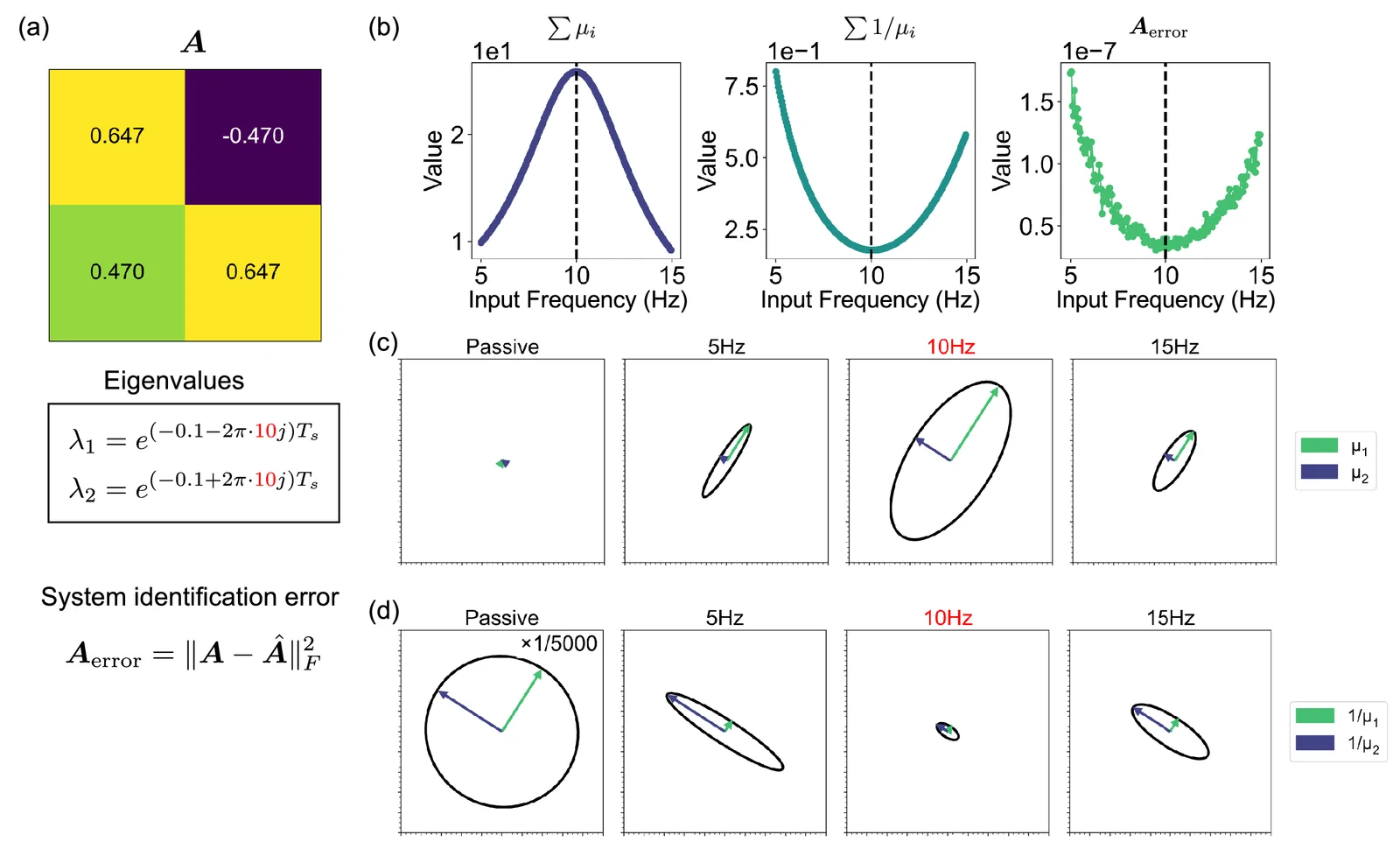
Error in the system matrix and eigenvalue-scaled ellipsoids. (**a**) State transition matrix $\boldsymbol {A}$ and its eigenvalues $\lambda _{i}$. (**b**) System frequency responses, showing the sum of eigenvalues of the state covariance matrix $\boldsymbol {\Sigma }_{\boldsymbol {X}}$ (left), the sum of inverse eigenvalues of the state covariance matrix $\boldsymbol {\Sigma }_{\boldsymbol {X}}$ (middle), and the system matrix error (right). (**c**) Eigenvalue ellipsoids of $\boldsymbol {\Sigma }_{\boldsymbol {X}}$ under passive, 5 , 10 , and 15 Hz conditions. (**d**) Reciprocal-eigenvalue ellipsoids of $\boldsymbol {\Sigma }_{\boldsymbol {X}}$ under passive, 5 , 10 , and 15 Hz conditions.

The simulation results show that an oscillatory input with the same frequency as a mode of the system matrix minimized the estimation error when the system had a single mode. Figure 4a shows the system matrix and its eigenvalues, which correspond to a single 10 Hz mode. We generated time series data using this system matrix and estimated the matrix. Figure 4b illustrates the changes in the sum of eigenvalues of the state covariance matrix $\boldsymbol {\Sigma }_{\boldsymbol {X}}$, denoted as $\sum _{i} \mu _{i}$, the sum of reciprocal eigenvalues $\sum _{i} (1/\mu _{i})$, and the estimation error defined by the Frobenius norm. The sum of eigenvalues is maximized by a 10 Hz sinusoidal input, while the sum of reciprocal eigenvalues is minimized. Consistent with these results, the estimation error is minimized by the 10 Hz sinusoidal input. As shown in Equation 9, the estimation error is proportional to the sum of the inverse eigenvalues. This tendency can be explained more clearly by plotting the eigenvalues as ellipsoids. We visualized the eigenvalues using eigenvalue-scaled ellipsoids, as shown in Figure 4c and d. Both the major and minor axes of the ellipsoids in Figure 4c are extended by the sinusoidal inputs, especially by the 10 Hz input. In contrast, the major and minor axes of the ellipsoids in Figure 4d are substantially shortened by the 10 Hz sinusoidal input.

**Figure 5.**
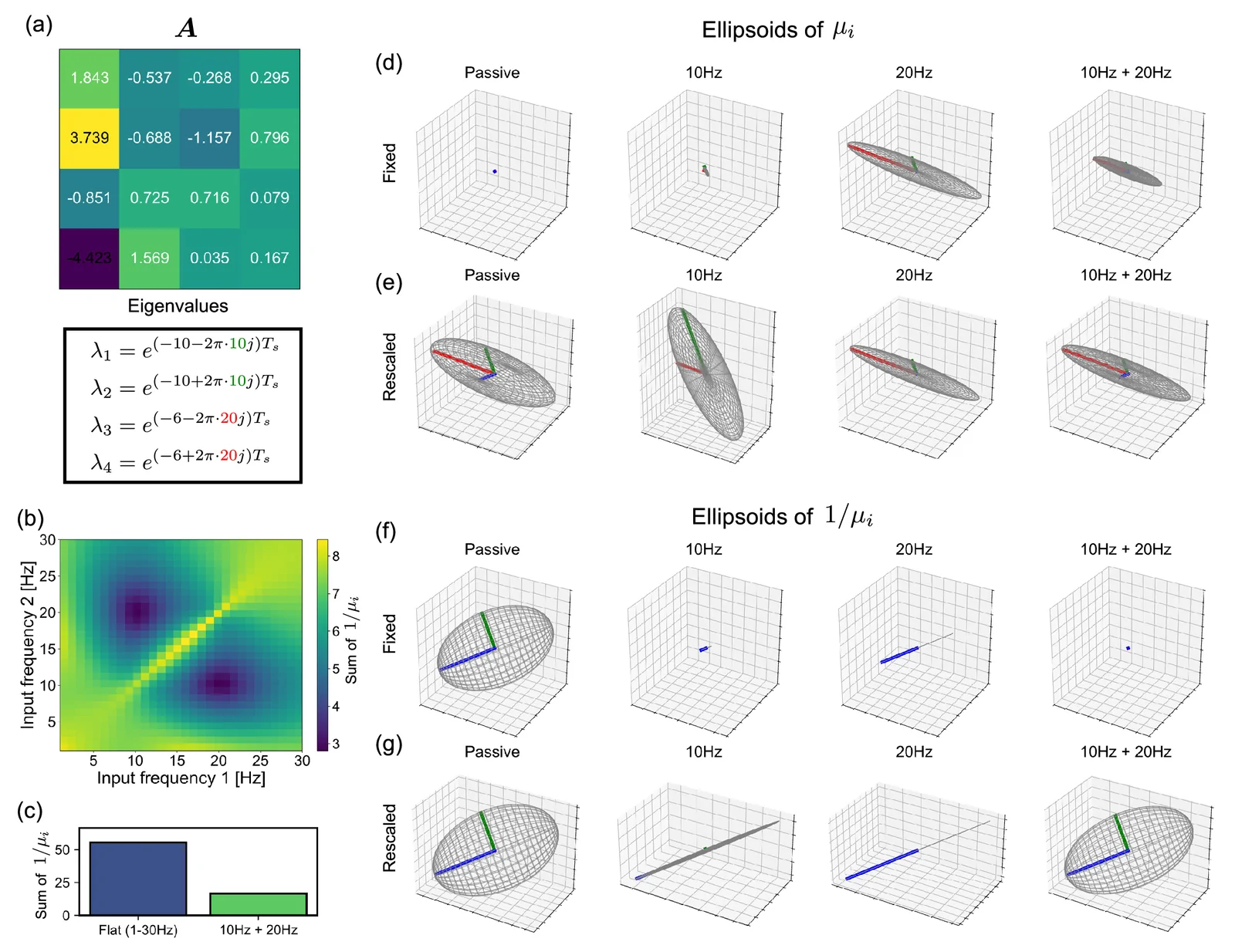
Simulation of a multi-mode system and eigenvalue-scaled ellipsoids. (**a**) System matrix $\boldsymbol {A}$ and its eigenvalues; the system exhibits two damped oscillatory modes at 10 and 20 Hz. (**b**) Theoretical estimation error, defined as $\sum _{i} (1/\mu _{i})$ for two-frequency input combinations; cooler colors indicate smaller estimation errors. (**c**) Comparison of the reciprocal eigenvalue sum for flat-spectrum versus composite-frequency inputs. (**d**) Eigenvalue-scaled ellipsoids (*μ*-scaled) under four input conditions (columns): Passive, 10 , 20 , and 10 Hz +20 Hz; ellipsoid axes align with the eigenvectors. (**e**) Relative-scale view of (**d**). (**f**) Eigenvalue-scaled ellipsoids (inverse-scaled, $1/\mu _{i}$) for the same four conditions. (**g**) Relative-scale view of (**f**).

When the system exhibits multiple modes, the input frequencies should be designed to reflect all these modes. We demonstrate this using the system matrix shown in Figure 5a, along with simulated time series and the corresponding matrix estimation. Because the system matrix contains two distinct pairs of eigenvalues (10 and 20 Hz), we constructed the evaluation input ***u*** as a combination of two frequencies. The sum of eigenvalue reciprocals is minimized only when the input contained both 10 and 20 Hz components; inputs with a single frequency yield larger values (Figure 5b). To further evaluate the effect of input design, we compared the two-frequency input with a flat-spectrum input (Figure 5c). When the total input energy was normalized, the two-frequency input achieves better identification performance than the flat-spectrum input, which is commonly employed in control and system identification studies. This result highlights the importance of tailoring the input spectrum to the system’s intrinsic dynamics rather than relying on uniform excitation. The superior performance of the two-frequency combination can be understood through the eigenvalue-scaled ellipsoids in Figure 5d–g. Figure 5d illustrates ellipsoids determined by the three dominant eigenvalues. The ellipsoid volume is expanded by three types of sinusoidal inputs (10 Hz, 20 Hz, and the combined input). However, the third axis (blue line) is not extended under single-frequency inputs, as shown in Figure 5e, leading to larger estimation errors. Small eigenvalues disproportionately increase the sum of reciprocal eigenvalues, as seen in Figure 5f and g. In these cases, the reciprocal eigenvalue-scaled ellipsoids are enlarged along the third axis (blue line) when only a single-frequency input is applied. By contrast, the combined input uniformly increases all eigenvalues, including the third, thereby successfully minimizing the reciprocal eigenvalue-scaled ellipsoid volume. These findings indicate that sinusoidal inputs should be designed to match multiple system modes rather than single modes. It should be noted that the true system modes cannot generally be known a priori. They must be identified through iterative experiments and refinement of the estimated system matrix. This practical procedure for optimal perturbation design is described in a later section (see Practical designing procedure for optimal perturbation inputs).

### Demonstration of location tuning

This section illustrates how our theoretical framework enables location-specific tuning of perturbation inputs within neural networks. As established earlier, perturbations should be designed so that the variance becomes large in all directions, rather than being confined to specific modes. Two candidate strategies arise naturally: directly stimulating the nodes associated with heavily damped modes to target those specific modes, or stimulating a hub node whose outgoing connections propagate the perturbation across the entire network.

**Figure 6.**
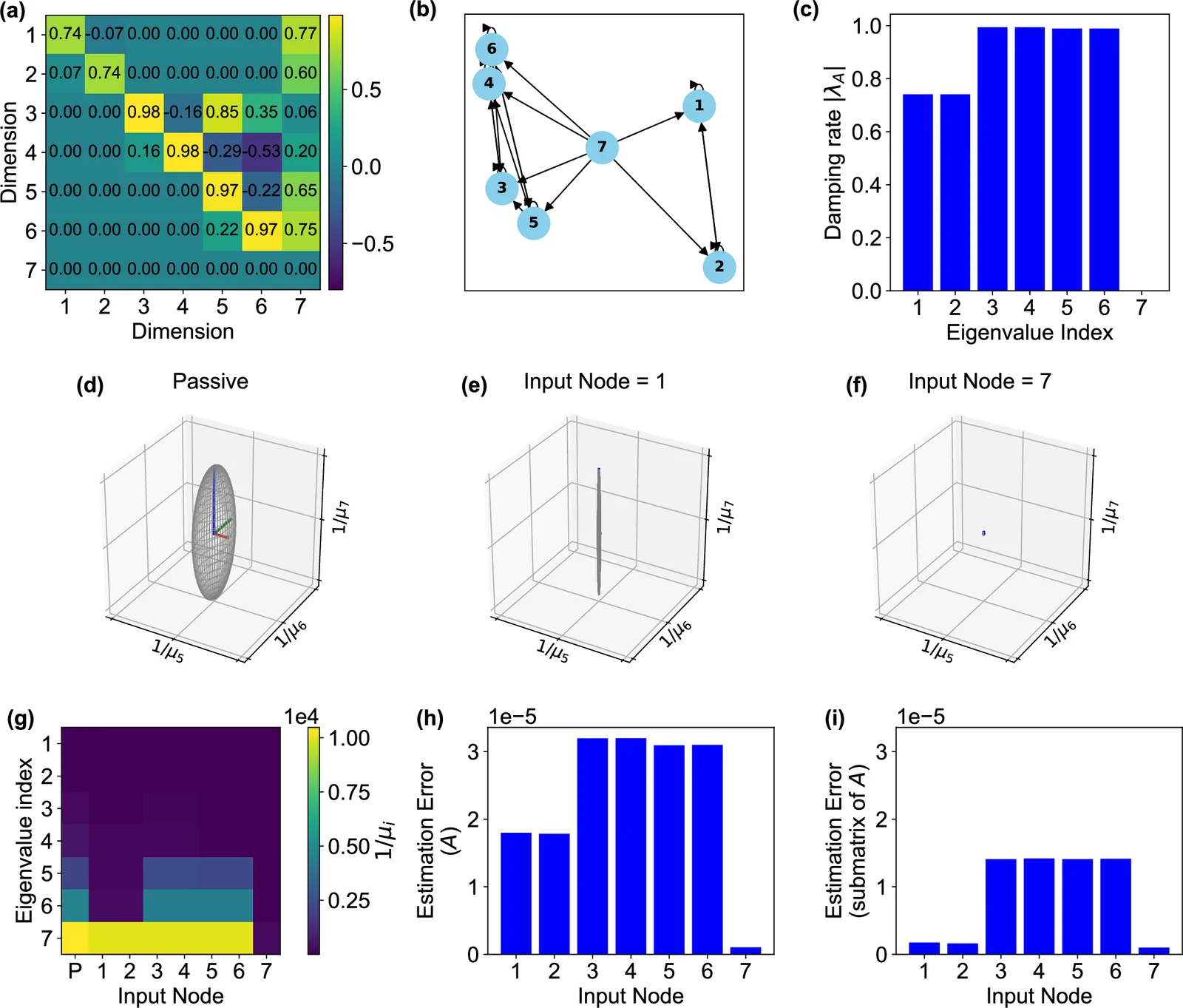
Optimal stimulation location (a hub node or the heavily-damped subnetwork) minimizes estimation error. A 7-node network with three oscillatory modes (15, 25, and 35 Hz) is used to identify effective perturbation locations. The 15 Hz mode (Nodes 1–2) is heavily damped; Node 7 is a hub with only outgoing edges. (**a**) The 7×7 connectivity matrix $\boldsymbol {A}$. (**b**) Graph representation. (**c**) Damping rates $|\lambda _{A}|$ of the eigenvalues of $\boldsymbol {A}$. (**d**–**f**) 3D ellipsoids whose axes are proportional to $1/\mu _{5}$, $1/\mu _{6}$, $1/\mu _{7}$ (reciprocals of the three smallest eigenvalues of $\boldsymbol {\Sigma }_{\boldsymbol {X}}$; larger axes indicate greater estimation error): (**d**) passive, (**e**) impulse to Node 1, (**f**) impulse to Node 7. (**g**) Heatmap of $1/\mu _{i}$ across input nodes (*P*=passive); uniformly small values appear only for Node 7. (**h**) Full-matrix estimation error per input node. (**i**) Estimation error for the submatrix excluding Node 7; Nodes 1, 2, and 7 achieve comparable errors, confirming that direct stimulation of the damped subnetwork matches hub stimulation.

To examine this relationship in practice, we constructed a network as shown in Figure 6a and b. The network consists of seven nodes, structured into three oscillatory modes (Nodes 1–6) and one hub node (Node 7). It is designed to have three oscillatory modes at 15, 25, and 35 Hz. Figure 6c shows the damping rates $|\lambda _{A}|$ of the eigenvalues of ***A***: the mode formed by Nodes 1 and 2 (15 Hz) is heavily damped, while those formed by Nodes 3–6 are moderately damped. Node 7 serves as a hub with only outgoing edges. Each node was individually perturbed by an impulse input. Here, an impulse input is defined as a Kronecker delta at t=0 with fixed amplitude α=10, with no external input applied at any subsequent time step.

Stimulating Node 7 (hub node) minimizes the total estimation error of ***A*** (Figure 6h), because its outgoing connections propagate the perturbation to all nodes and re-excite every dynamical mode. Figure 6d–f illustrate how the choice of input node shapes the smallest eigenvalues of $\boldsymbol {\Sigma }_{\boldsymbol {X}}$. Because the 15 Hz mode (Nodes 1–2) is heavily damped, it decays rapidly and contributes almost no variance to $\boldsymbol {\Sigma }_{\boldsymbol {X}}$ under passive conditions; as a result, the fifth through seventh eigenvalues $\mu _{5}$–$\mu _{7}$ are small and their reciprocals $1/\mu _{5}$–$1/\mu _{7}$ are large, producing the elongated ellipsoid seen in Figure 6d. When Node 1 receives an impulse (Figure 6e), the eigenvalue associated with the 15 Hz mode grows and the $1/\mu _{5}$ and $1/\mu _{6}$ axes shrink; however, the $1/\mu _{7}$ axis—which corresponds to directions that Node 1 alone does not excite—remains large. Stimulating Node 7 (Figure 6f) re-excites all six oscillatory nodes through its outgoing connections, simultaneously reducing all three axes. Figure 6g confirms this pattern across all candidate nodes: the contribution $1/\mu _{i}$ remains uniformly small only when Node 7 receives the impulse. Figure 6h and i show that Node 7 achieves the smallest full-matrix estimation error, followed by Nodes 1 and 2, which directly drive the heavily damped 15 Hz mode.

As this simulation represents only one example of location design, its limitations and the corresponding countermeasures should be stated. In actual experiments, the most effective perturbation location depends on factors, such as hub-node connectivity and modal damping rates, and ***B*** itself may not always be known a priori. In such cases, approaches, such as iterative optimization of $\mathrm{tr}(\boldsymbol {\Sigma }_{\boldsymbol {X}}^{-1})$ (described in a later section) and joint estimation of ***A*** and ***B*** (Appendix 2.3) provide systematic alternatives. Nevertheless, the results presented here provide an intuitive guideline: stimulation directed at hub nodes or at nodes driving heavily damped modes effectively excites the full set of dynamical modes and minimizes estimation error.

### Neural state classification

We demonstrate the effectiveness of our framework by applying it to a neural state classification problem. Neural state classification is crucial for diagnosing illnesses and assessing brain conditions in many experimental neuroscience settings (Casali et al., 2013; Hallett et al., 2017; Shikauchi et al., 2025; Angulo-Sherman et al., 2017). Some of these studies have already applied arbitrary perturbation inputs for classification, but not optimal perturbation inputs. By simulating such real-world applications, we demonstrate how well optimal perturbation design can contribute to neuroscience research.

**Figure 7.**
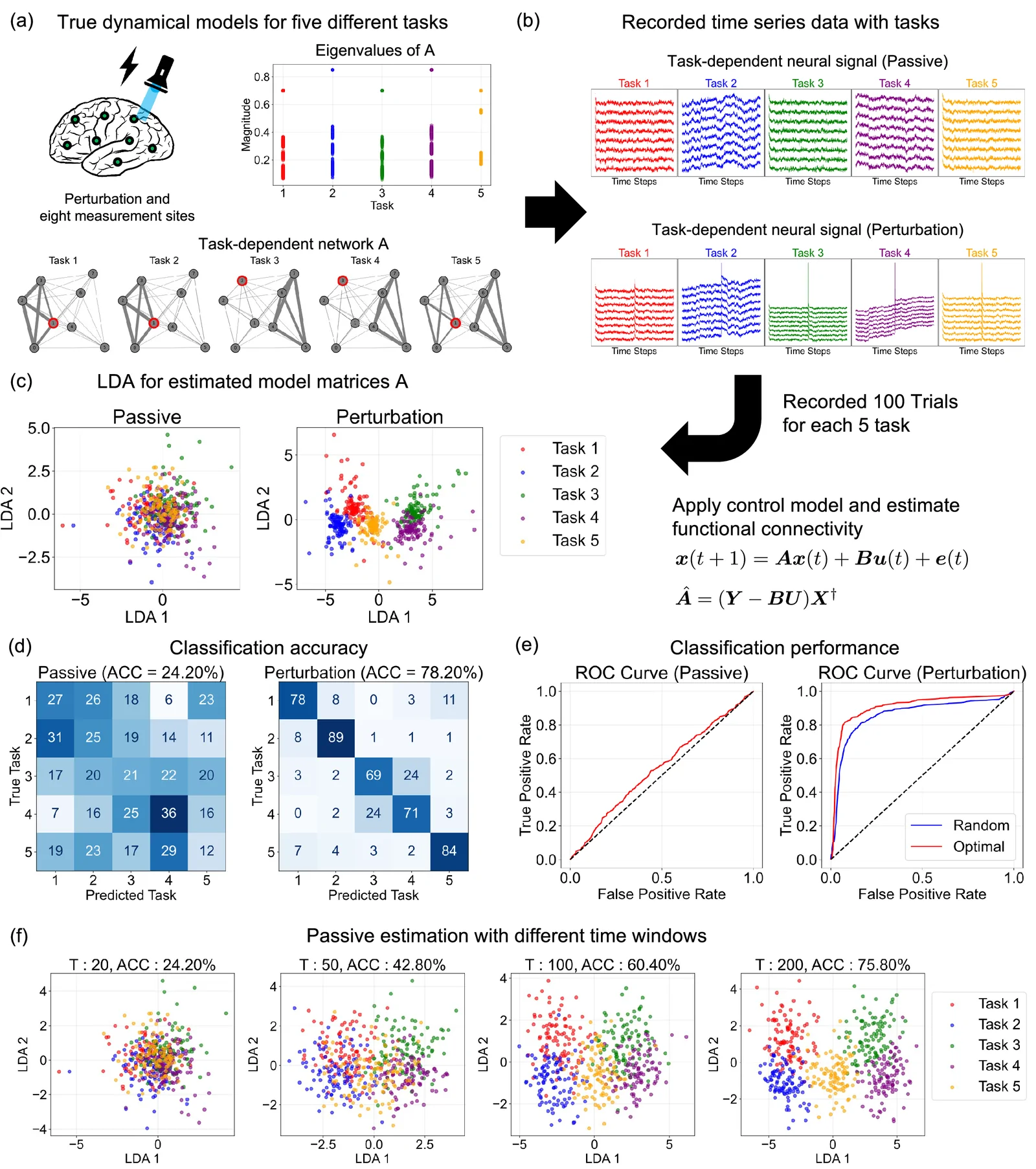
Neural state classification enhanced by perturbation-aided model estimation. (**a**) Ground-truth dynamics models defining five distinct task conditions, each with unique eigenvalue distributions and network structures of matrix $\boldsymbol {A}$. The optimally excited node is highlighted in red. (**b**) Simulated neural states (activity) induced by the five task conditions, shown separately for the passive (top) and perturbed (bottom) regimes. (**c**) Estimated model matrices $\boldsymbol {A}$ visualized using linear discriminant analysis (LDA). (**d**) Confusion matrices showing classification accuracy for the passive condition (left) and the optimally designed perturbation condition (right). (**e**) ROC curves for the passive condition (left) and a comparison of random and optimal perturbation conditions (right). (**f**) LDA plots for passive condition with increasing time window length, showing clustering and classification accuracy as the time window length T increases from 20 to 200.

We designed a neural network with clearly distinct task conditions and considered a simulation setting in which these conditions are classified using signals of a fixed duration. These distinct task conditions consist of five types, each defined by a unique linear dynamical system characterized by differing eigenvalue spectra and connectivity topologies of matrix ***A*** (Figure 7a). These task conditions are intended to mimic different cognitive or behavioral contexts. For example, in a typical motor task experiment, such conditions could correspond to motor execution or imagery involving the left or right hand, or resting state (Shikauchi et al., 2025; Angulo-Sherman et al., 2017). The neural signals were simulated under five different task conditions and two stimulation conditions: passive observation and external perturbation. Perturbation was applied as impulse-type inputs, such as TMS. The stimulus location was determined for each task condition by applying an impulse to each node and selecting the one that minimized $\mathrm{tr}(\boldsymbol {\Sigma }_{\boldsymbol {X}}^{-1})$. The resulting time-series data are shown in Figure 7b. Using this data, we estimated the underlying dynamical system via a control-based identification approach presented in Equation 4, which corresponds to an estimation of functional connectivity.

The simulation demonstrates that classification performance under perturbation is superior to that under the passive condition, both in visual inspection and in quantitative evaluation. As revealed by linear discriminant analysis (LDA) projection, task clusters in the passive condition exhibit substantial overlap, whereas perturbation leads to clear separation among the tasks (Figure 7c). Classification with LDA and 10-fold cross-validation shows the average accuracy is substantially higher in the perturbation condition (78.20%) compared to the passive condition (24.20%), as shown in Figure 7d. ROC curve analysis further confirms that perturbation substantially improves discriminability across all tasks and outperforms the random perturbation condition (Figure 7e). These results can be explained by Equation 8: the designed perturbation inputs increase the eigenvalues of the covariance matrix, including even the smallest ones, which in turn leads to a decrease in the estimation error of ***A***.

To obtain estimates under the passive condition that are comparable to those derived under perturbation, it is necessary to experimentally observe extensive time-series data. Figure 7f illustrates the LDA projection and classification accuracy for different time-series lengths. The leftmost LDA plot (T=20) corresponds to the passive condition shown in Figure 7c, indicating that the estimation performance in the passive condition becomes comparable to that in the perturbation condition only when the time window reaches approximately T=200, a 10-fold increase compared to T=20. While the absolute duration depends on the interpretation of the time unit, such time windows may not be prohibitive in some experimental settings. Nevertheless, our results consistently show that passive observation requires substantially longer recordings to achieve comparable performance, highlighting the efficiency of the perturbation-based approach when the available data length is limited.

### Neural state transitions via optimal control

**Figure 8.**
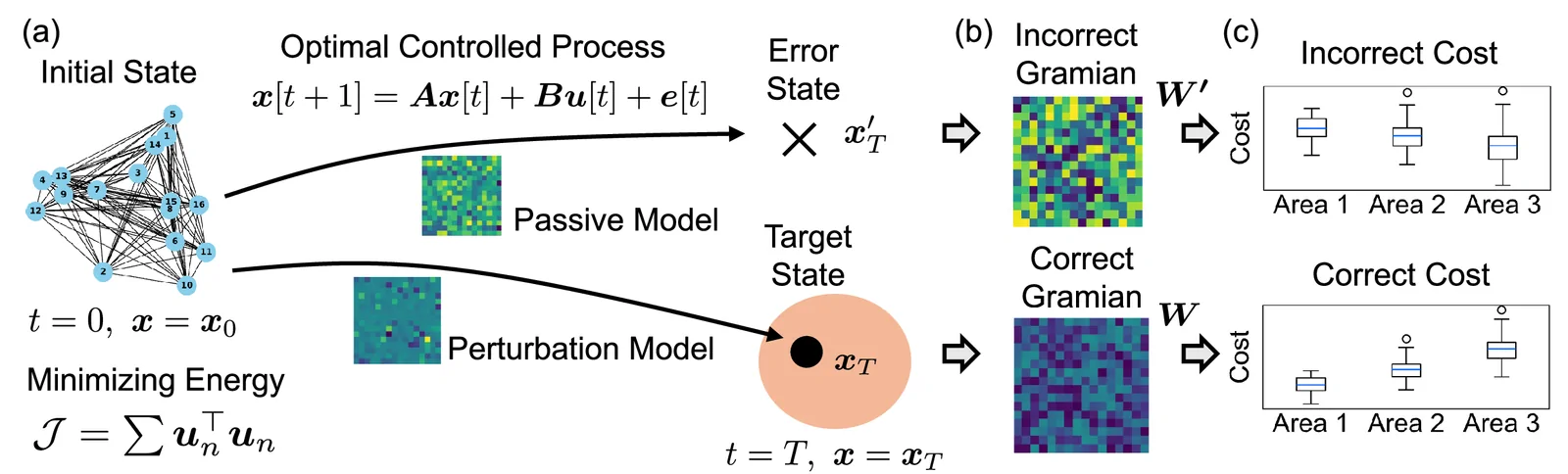
Effects of system identification on network control theory. (**a**) State transitions using models identified from the passive and perturbed conditions. (**b**) Controllability Gramians with two models. (**c**) Control costs computed from the estimated controllability Gramians.

This section demonstrates the practical utility of our framework by applying it to a neural state control problem (Gu et al., 2015; Karrer et al., 2020). Here, we estimate the system parameters from both passive and perturbation states. Then, we determine optimal control inputs which control the neural states to desired targets, and associated control costs. By using perturbation inputs, ***A*** is accurately estimated, which in turn allows precise determination of the optimal inputs and the corresponding controlled state transitions (Figure 8a). Moreover, use of the perturbation approach to derive the model allows the controllability Gramian (Gu et al., 2015; Shikauchi et al., 2025), as well as the estimation of control costs for each network area, to be more reliably assessed (Figure 8b and c). This simulation underscores the importance of accurate system identification for achieving optimal control and reliable estimation of neural functions. Throughout this section, we use the term *perturbation input* to refer to the input used for system identification and *control input* to refer to the input used for state transitions.

**Figure 9.**
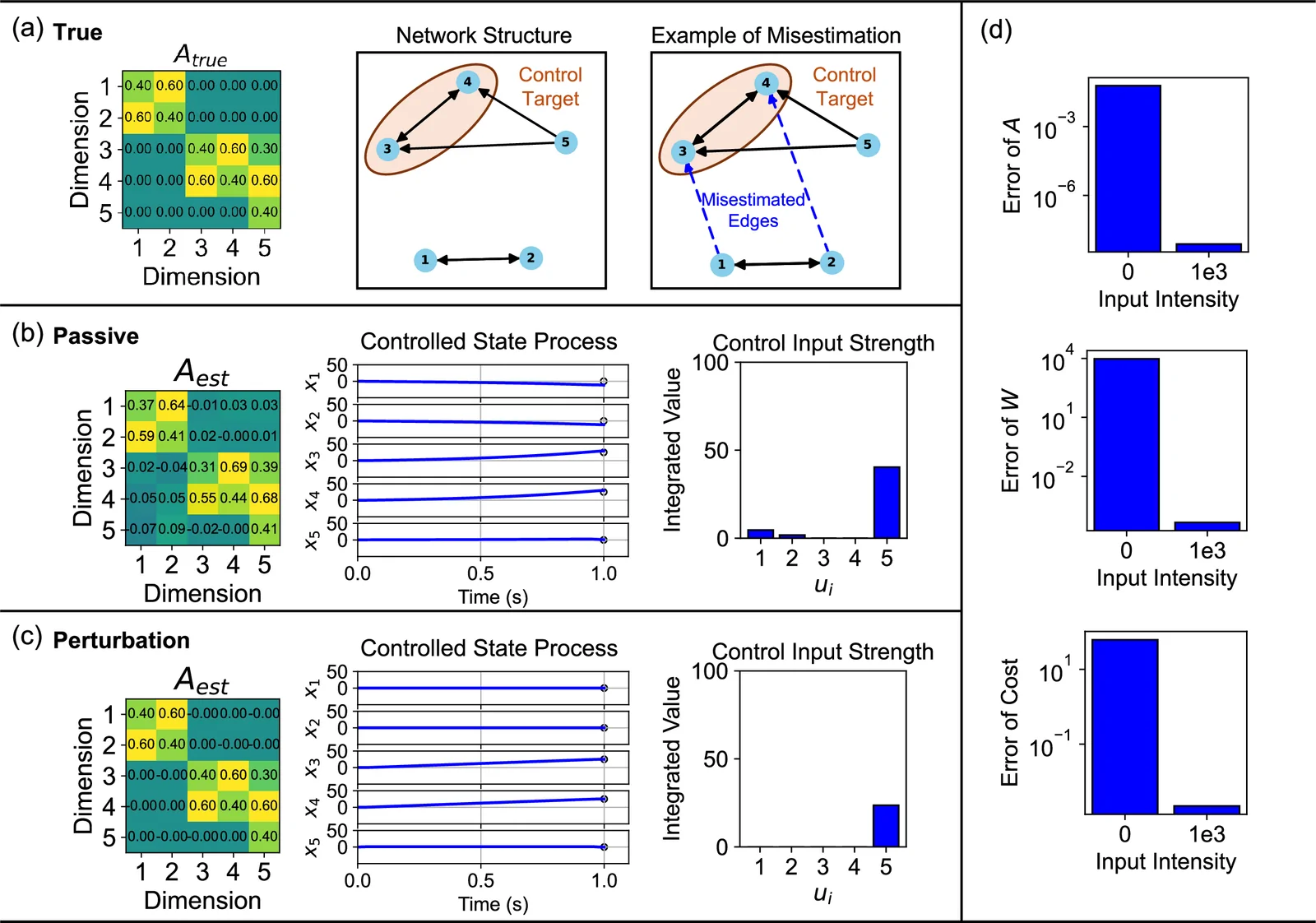
Accurate system identification via perturbation enables precise optimal control. (**a**) True matrix $\boldsymbol {A}$ and its network structure. Nodes 1, 2, and 5 are controllable nodes. Nodes 3 and 4 are targets controlled by the determined control input. When the connectivity matrix is misestimated, the structure has edges between nodes 1 and 2, and nodes 3 and 4. (**b**) Matrix estimation failure under passive conditions and controlled state transitions. The control objectives are plotted as white circles. (**c**) Successful matrix estimations and controlled state transitions with sufficiently strong impulse inputs ($\alpha = 10^{3}$). (**d**) Errors in the estimated matrices $\boldsymbol {A}$ and $\boldsymbol {W}$ and the control cost (average controllability).

We designed a network as shown in Figure 9a to clearly show the differences in system identification between passive and perturbation states. The network consists of two disconnected groups. Nodes 1 and 2, as well as nodes 3, 4, and 5, are each connected separately, with no connections between these groups, as shown in matrix ***A***. We assumed a known input matrix ***B*** in which nodes 3 and 4 cannot be directly controlled. In this scenario, the optimal control strategy to manipulate nodes 3 and 4 is to apply inputs to node 5. However, if the system identification is inaccurate, there is the possibility that control will be attempted through nodes 1 and 2, which are disconnected from nodes 3 and 4. These matrices were estimated from the time series signals generated by simulation. We generated passive and perturbation states to estimate the parameter matrices. The perturbation condition uses an impulse input with sufficient intensity ($\alpha =10^{3}$). By using the estimated system model, we determined optimal control inputs, then evaluated the state transitions and associated costs. The controlled transition test was run with T=50. The control objective was to set nodes 3 and 4–25 while keeping all other nodes at 0 without any movement.

The simulation results show that perturbation-based estimation enables the precise control of neural states. If the estimation of ***A*** is inaccurate, the state transitions under optimal control cannot be executed correctly (Figure 9b). Nodes 3 and 4 fail to reach the target, and unnecessary activations occur in nodes 1 and 2. Furthermore, unnecessary control inputs are required in nodes 1 and 2. On the other hand, when the model is correctly estimated using a high-strength impulse, the resulting control inputs enable accurate state transitions (Figure 9c). In this case, the strength of the impulse is correctly concentrated on node 5. As the error in matrix ***A*** increases, the estimation errors of the controllability Gramian ***W*** lead to inaccuracies in estimating the control cost (Figure 9d). Here, we adopt average controllability as an example measure of control cost (Gu et al., 2015; Moradi Amani et al., 2024). The key point here is that, since the controllability Gramian involves multiple products of ***A*** (Equation 21), even small errors in ***A*** can result in significant discrepancies. Therefore, when estimating optimal control input for neural transitions or control costs, it is more appropriate to identify those model parameters with at least arbitrary perturbations, and ideally with designed perturbations.

### Practical designing procedure for optimal perturbation inputs

This section presents a practical framework for designing optimal perturbation inputs to estimate neural dynamics. The practical framework progressively refines the input signal through repeated cycles of model identification and perturbation input design. By leveraging this alternating scheme, each iteration utilizes the current model estimate to design a more informative input for the next round of identification. We also focus on perturbations characterized by a broad and uniform frequency distribution, a form commonly adopted in control engineering and conceptually analogous to transcranial random noise stimulation (tRNS) in neuroscience (Paulus, 2011; Paulus et al., 2016). As shown in Figure 5 and Appendix 2—figure 4, the designed composite-wave input allows for more accurate model estimation than the uniform-frequency input when their total input energies are equal.

**Figure 10.**
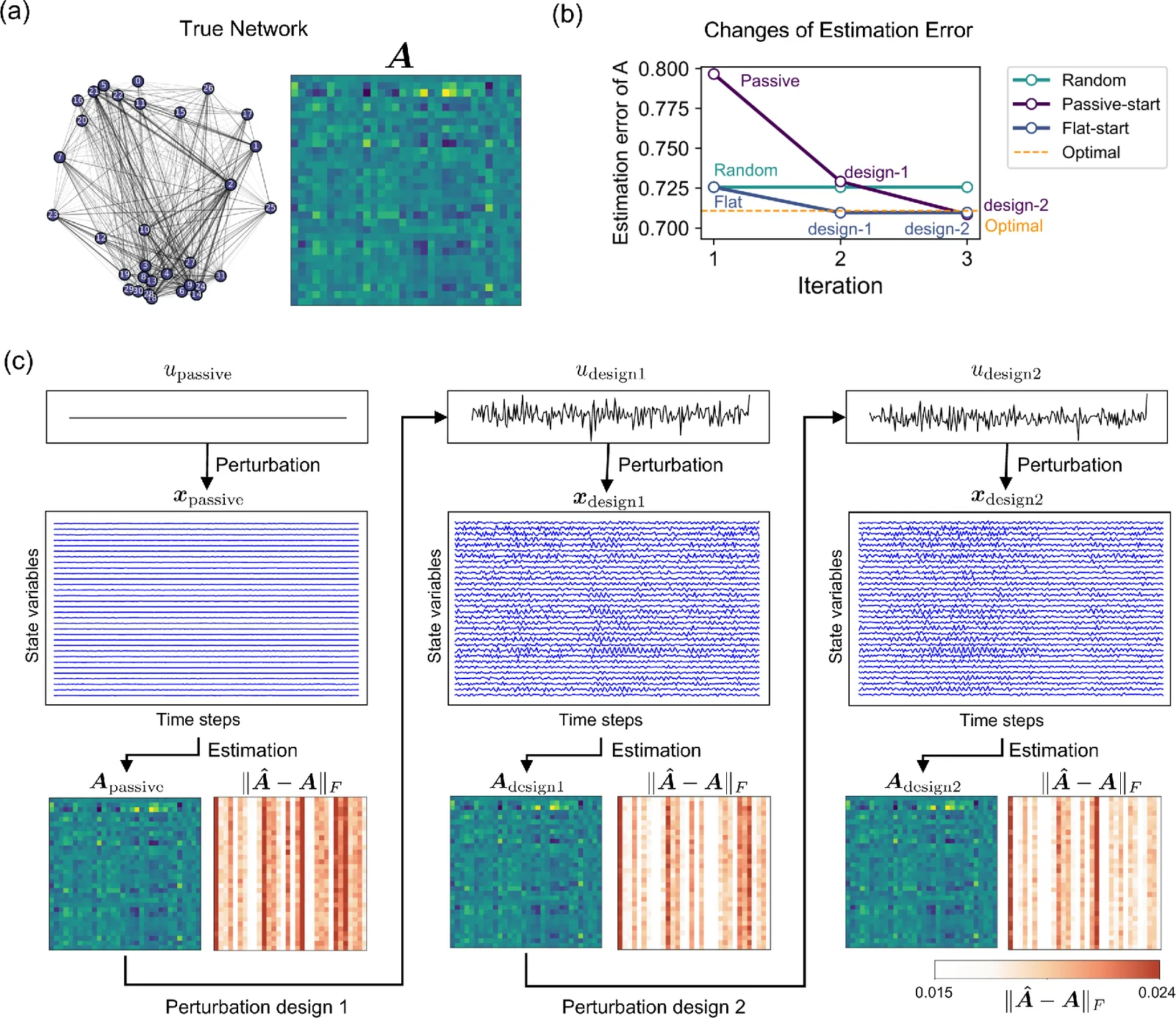
Iterative perturbation design converges to the theoretical optimum in a 32-channel neural network simulation. (**a**) True matrix $\boldsymbol {A}$ and its network structure, which was designed to include both oscillatory and attenuating components. (**b**) Comparison of estimation errors. The purple line indicates the iterative design process initialized from the passive-state estimate (Iteration 1). The blue line shows the iterative design starting from a flat-spectrum input (Iteration 1). Both processes converge towards the theoretically optimal error (dashed line) as the design is refined (design-1, design-2). The third line (labeled ‘Random’) represents a baseline in which a single randomly chosen node is stimulated with a flat-spectrum input throughout all iterations. (**c**) Schematic of the iterative procedure. An initial estimated system matrix ($\boldsymbol {A}_{\mathrm{passive}}$) is used to design the first perturbation input ($\boldsymbol {u}_{\mathrm{design1}}$), which yields a better estimate ($\boldsymbol {A}_{\mathrm{design1}}$), and the cycle repeats.

Iterative refinement of both the perturbation design and the estimation process progressively improves the accuracy of ***A***. The time-series data is collected from 32 points, where the matrix ***A*** (Figure 10a) is designed to have 16 oscillatory modes (i.e. 16 complex-conjugate eigenvalue pairs, yielding 32 eigenvalues in total). In this simulation, one stimulation session is applied to a single node at each design step. At each iteration, the stimulation is designed as a composite-frequency sinusoidal input encompassing all modes of the estimated $\boldsymbol {\hat {A}}$, and the target node of the perturbation is determined through numerical optimization that minimizes $\mathrm{tr}(\boldsymbol {\Sigma }_{\boldsymbol {X}}^{-1})$. Appendix 2—table 1 summarizes the simulation conditions, including measurement duration and number of trials used in each simulation. The stimulation duration was set equal to the measurement duration.

The following procedure outlines a practical approach (Figure 10c) for this simulation, aiming to precisely estimate the matrix by designing an optimal perturbation input (assuming frequency stimulation, such as tACS).

1. Record spontaneous activity as the passive state and estimate ***A***_passive_.
2. Based on the estimated matrix, the optimal frequency and target node of the perturbation input are determined through numerical optimization following Equation 9.
3. The designed perturbation ***u***_design1_ is applied to the system, and a more precise matrix ***A***_design1_ is identified.
4. The obtained matrix ***A***_design1_ is again used to design a new perturbation input ***u***_design2_ and estimate a more accurate matrix ***A***_design2_. This iterative process can be continued until convergence criteria, such as changes in ***A***, are met.

The proposed framework improved estimation accuracy iteration by iteration. As shown in Figure 10b, the estimation error converges toward the theoretical optimum (dashed line) with each design iteration. Here, the theoretical optimum is obtained from a simulation in which the input is constructed as a composite sinusoid covering all dynamical modes of the true matrix ***A***, and the stimulation location is numerically selected to minimize $\mathrm{tr}(\boldsymbol {\Sigma }_{\boldsymbol {X}}^{-1})$. The error from the second iteration (second design) is smaller than that from the first (first design), which in turn is substantially lower than the initial estimate. This convergence holds both when starting from the passive condition and when starting from a flat-spectrum input (a practical approach analogous to tRNS). While the flat-spectrum input (blue line, Iteration 1) provided a better starting point than passive data, the designed input from the initial flat-spectrum estimate further reduced the error by the second iteration. Furthermore, the converged estimation error was lower than that of a random stimulation baseline—in which a single randomly chosen node was stimulated using a flat-spectrum (all-frequency) input throughout all iterations (green line)—whose estimation error remained approximately constant across iterations. This contrast directly demonstrates that the benefit of the proposed framework arises specifically from model-based optimization of both the spatial (node selection) and spectral (frequency composition) stimulation parameters. These results should be interpreted as a case-specific illustration rather than a universal gain, as the magnitude of improvement depends on factors, such as network structure, recording duration, noise level, and stimulation design. This simulation demonstrates that our perturbation design framework enables the step-by-step refinement of system identification even without prior knowledge of the system.

## Discussion

This study addressed the challenge of designing optimal perturbations for effectively identifying neural system dynamics. We introduced a framework for estimating the optimal perturbation input for identification of neural systems. Our findings demonstrated that incorporating perturbation inputs, including TMS, tDCS, and tACS, significantly improves identification accuracy. Specifically, alignment of their parameters with the intrinsic frequencies of matrix ***A***, high input intensity, and targeted inputs directed at nodes that efficiently excite heavily damped dynamical modes (either nodes directly associated with those modes or hub nodes whose outgoing connections propagate the perturbation throughout the network) enhances system identification. Furthermore, we outlined an approach for designing the optimal perturbation input by iterating system identifications with perturbations, and demonstrated that the approach progressively decreases parameter estimation error. Beyond the parameter identification of neural systems, our findings provide insights into how these estimated parameters influence optimal control theory in neuroscience. These results emphasize the potential of perturbation input design to advance understanding of neural dynamics.

The assumption of linearity and first-order autoregressiveness in neural dynamics warrants discussion. Biological signals often exhibit complex and nonlinear interactions that cannot be fully captured by linear models. Nevertheless, prior studies have demonstrated that nonlinear behaviors can frequently be approximated using linear models (Honey et al., 2009; Gu et al., 2015; Fernández Galán, 2008). Linear models are particularly effective for providing accurate approximations of nonlinear systems within a specific operating range (Khalil, 2002). Therefore, establishing theoretical foundations based on linear assumptions can contribute to the development of theories for nonlinear systems or be effectively utilized in their advancement. To extend these theories into the nonlinear domain, it would be necessary to incorporate advanced frameworks, such as the Koopman operator (Koopman, 1931; Chow et al., 2024) and system identification methods leveraging machine learning techniques (Suk et al., 2016; Glaser et al., 2019). On the other hand, discussions regarding higher-order VAR models are relatively straightforward due to the simplicity of the estimation method. Previous studies involving actual functional data have employed VAR models of an order greater than one (Tseng et al., 1995; Chang et al., 2012; Shakeel et al., 2021). The framework proposed in this paper can be naturally and meaningfully extended to higher-order VAR models, broadening its applicability to more complex temporal dependencies.

Safety and experimental constraints play a pivotal role in the practical implementation of this framework, influencing both the scope and design of potential applications. In biological systems, stimulation parameters, such as amplitude, frequency, and location, must adhere to stringent safety thresholds to avoid adverse effects, such as tissue damage or unintended physiological responses (Rocchi et al., 2022; Bikson et al., 2016). For stimulus intensity, our proposed framework suggested that a stronger stimulus improves system identification; however, an excessively strong stimulus poses safety risks (Antal et al., 2017). With respect to sinusoidal input, stimuli are typically applied within ranges corresponding to neural activity. The optimal input frequencies derived from the proposed theory are expected to align with these ranges, suggesting that the proposed framework can be implemented without major complications (Antal et al., 2017; Mager et al., 2018). Furthermore, the selection of stimulation sites is often dictated by experimental accessibility or ethical considerations (Rossi et al., 2009; Heinrichs, 2012). Based on these constraints, it is necessary to determine the most appropriate stimulation parameters to achieve optimal system identification.

Two directions warrant further investigation: extending the framework to partial observability, and validating it through stimulation experiments. In experimental neuroscience, recordings are often limited to a subset of neural populations, resulting in partial observability. A growing body of work has leveraged delay-embedding techniques, represented by Takens' embedding theorem (Takens, 1980), to reconstruct hidden dynamics from partial observations (Arbabi and Mezić, 2017; Brunton et al., 2017; Ostrow et al., 2024; Huang et al., 2025). Applying such techniques enables the estimation of the full connectivity matrix, thereby extending our framework to settings with partial observability. The second direction concerns experimental validation. Validating a theoretical framework through experimental design is essential for bridging the gap between theory and practice. Recent research involving some of the present authors has demonstrated that TMS can facilitate the discrimination of neural states (Shikauchi et al., 2025). Future experiments can build on this finding by incorporating passive, arbitrary, and designed optimal stimuli to estimate the connectivity matrix, and then comparing the ability of these matrices to distinguish different neural states. If these proposed evaluations demonstrate the practical effectiveness of our theory, it could then be applied to actual experiments. As shown in Figure 10, a preliminary connectivity matrix is obtained through preliminary experiments to design optimal perturbations. These perturbations would then be applied in the main experiments, improving the estimation of connectivity matrices and neural dynamics. Experiments using our approach will enable the more precise and comprehensive analysis of brain and neural function, and in turn facilitate valuable new insights into human cognition and behavior.

## Methods

### State vector simulation and model estimation

To investigate appropriate perturbation inputs for the identification of neural systems, we constructed neural dynamics using matrices ***A*** and ***B*** to generate the temporal evolution of state variables. From the generated dynamics, we estimated the model matrix ***A***, while assuming that the model matrix ***B*** was known.

Given an initial condition $\boldsymbol {x}(0)$, the neural dynamics were generated according to the true matrices ***A*** and ***B*** and the governing equation for $\boldsymbol {x}(t)$ (Equation 1), with a predefined noise time series $\boldsymbol {\xi }(t)$ added at each time step. For the discrete-time simulations, the sampling rate was set to an appropriate value for each simulation. The model matrix ***A*** was then estimated from the generated time-series states $\boldsymbol {x}(t)$ and applied perturbation inputs $\boldsymbol {u}(t)$ using the OLS.

Both the generation of neural dynamics and the model estimation were repeated for a predefined number of trials, with variations in initial conditions and noise sequences. The estimation errors of the matrices were quantified using the Frobenius norm. Details of the simulation parameters are provided in Appendix 2.1.

### Eigenvector alignment for eigenvalue-scaled ellipsoids

To examine the relationship between the eigenvalues $\mu _{i}$ of the state covariance matrix $\boldsymbol {\Sigma }_{\boldsymbol {X}}$ and the input frequencies, we plotted an eigenvalue-scaled ellipsoid, thereby providing an intuitive representation of optimal input design. However, the eigenvectors of $\boldsymbol {\Sigma }_{\boldsymbol {X}}$ are not fixed; they may vary depending on the applied input, which complicates direct comparison across conditions. To enable consistent comparison of eigenvalues across input conditions, we reordered eigenvalues according to the similarity of their associated eigenvectors to those obtained in the passive condition, which served as the reference basis. For each reference eigenvector $\boldsymbol {t}_{i}$, we identified the most similar eigenvector $\boldsymbol {u}_{j}$ from the input condition by maximizing the absolute inner product

(16) $$\displaystyle j^{\ast } = \arg \max _{j} \left | \boldsymbol {t}_{i}^{\top } \boldsymbol {u}_{j} \right | .$$

The eigenvalue $\mu _{j^\ast }$ corresponding to $\boldsymbol {u}_{j^\ast }$ was then assigned to the (i)-th position of the reordered list. Each $\boldsymbol {u}_{j}$ was used only once, ensuring a one-to-one correspondence between reference and input eigenvectors. This alignment resolves the permutation and sign ambiguities inherent in eigendecomposition, and allows eigenvalues to be compared across conditions along a common axis.

### Optimal control and network controllability

Accurately estimating dynamical models under perturbation not only facilitates precise inference of the neural dynamics but also contributes to the accurate estimation of control inputs for neural state transitions. Recently, the control theory which utilizes the controllability Gramian and control costs is applied for neuroscience, and provides insights into the efficiency and feasibility of inducing specific neural state transitions (Gu et al., 2015; Karrer et al., 2020) with some limitations (Tu et al., 2018; Suweis et al., 2019). For clarity, we refer to the input for system identification as the *perturbation input* and the input for state control in the optimal control theory as the *control input*.

The optimal control input is derived under the condition of assuming the linear system is controllable. We consider the sequence of control inputs that minimizes the following cost function (input energy minimization) while driving the state from the initial state $\boldsymbol {x}_{0}$ to the terminal state $\boldsymbol {x}_{T}$:

(17) $$\displaystyle  \mathcal{M}(\{\boldsymbol {u}(t)\}_{t=0}^{T-1}) = \sum _{t=0}^{T-1}\boldsymbol {u}(t)^{\top } \boldsymbol {u}(t),$$

under

(18) $$\displaystyle \boldsymbol {x}(0) = \boldsymbol {x}_{0}, \quad \boldsymbol {x}(T) = \boldsymbol {x}_{T}.$$

Here, the initial and terminal state conditions represent constraints on the optimization problem. This problem is expressed as follows:

(19) $$\displaystyle \boldsymbol {u}(t)^{*} = \underset {\boldsymbol {u}}{\text{arg min }}\mathcal{M}(\{\boldsymbol {u}(t)\}_{t=0}^{T-1}).$$

The constrained optimization problem can be solved using Lagrange multipliers. The optimal control input is given by

(20) $$\displaystyle \boldsymbol {u}(t)^{*} = \boldsymbol {B}^{\top } (\boldsymbol {A}^{\top })^{T-t-1}\boldsymbol {W}^{-1}(\boldsymbol {x}(T) - \boldsymbol {A}^{T} \boldsymbol {x}(0)),$$

where ***W*** is the controllability Gramian, defined as:

(21) $$\displaystyle  \boldsymbol {W}= \sum _{t=0}^{T-1}\boldsymbol {A}^{t} \boldsymbol {B}\boldsymbol {B}^{\top } (\boldsymbol {A}^{\top })^{t}.$$

The controllability Gramian plays a pivotal role in determining the optimal control and control input cost. It has been used to identify the functional roles of individual brain regions (Gu et al., 2015; Shikauchi et al., 2025).

### Average controllability for control cost

While the optimal control problem (Equation 19) calculates the specific input energy for a given state transition, a more general, state-independent metric is often used to characterize the system’s overall controllability. This metric, average controllability, is a state-independent metric used to quantify the system’s overall ease of control (Gu et al., 2015; Moradi Amani et al., 2024). It is defined as the trace of the controllability Gramian:

(22) $$\displaystyle  \text{Average Controllability} = \mathrm{tr}(\boldsymbol{W}) = \sum_{i=1}^{N}\lambda_i(\boldsymbol{W})$$

A larger trace indicates that the system is, on average, more controllable (i.e. can be moved to various states with less input energy). This metric is, therefore, used as a measure of control efficiency.

## Figures and Tables

**Appendix 2—figure 1.**
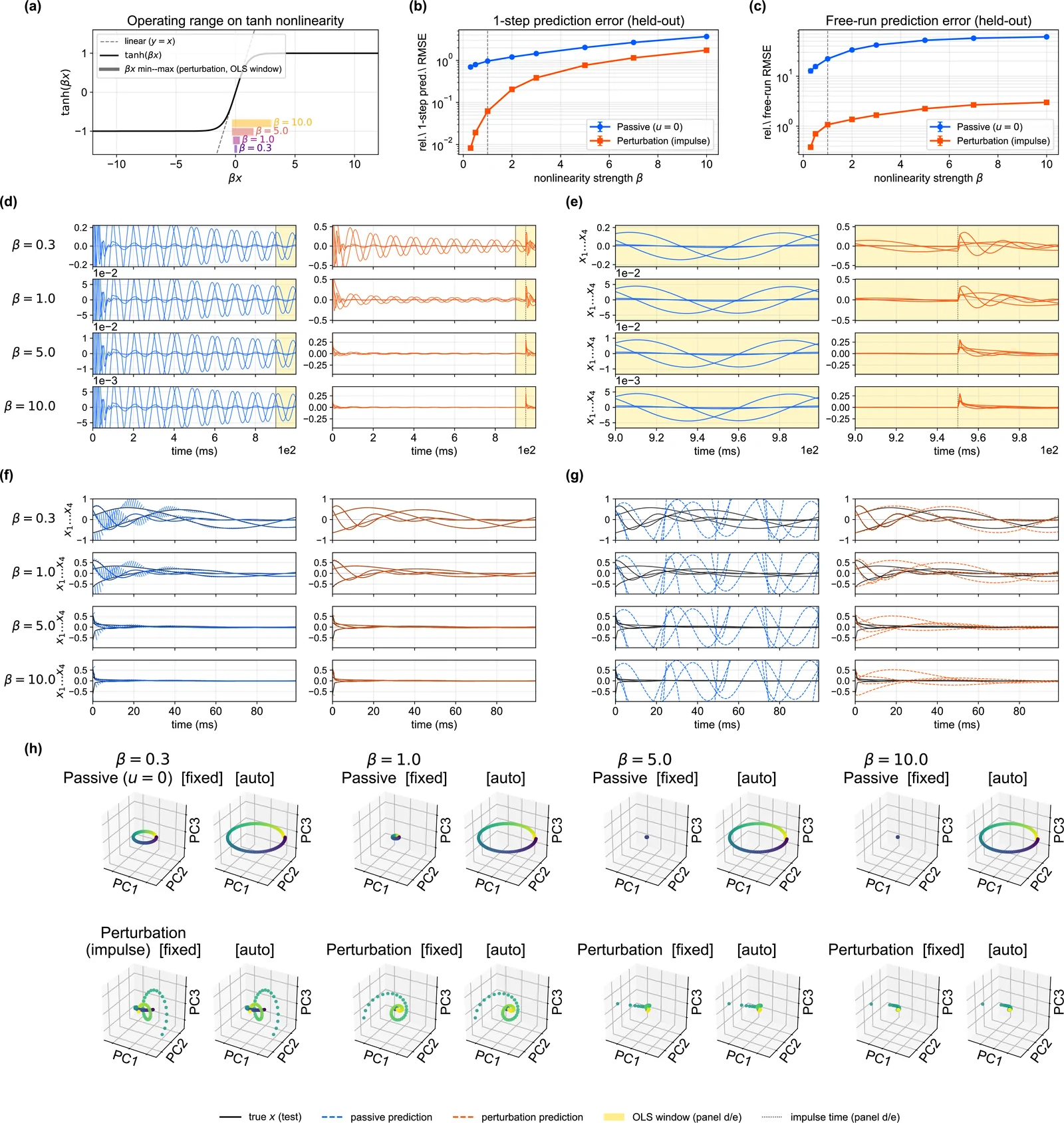
Robustness of the perturbation framework to nonlinear state dynamics. (**a**) The tanh nonlinearity with the min–max envelope of $\beta \boldsymbol {x}$ within the ordinary least squares (OLS) window shaded for $\beta \in \{0.3,1,5,10\}$. (**b**) Relative 1-step prediction RMSE vs. *β*. (**c**) Relative free-run (rollout) RMSE vs. *β*. (**d**) Training trajectories within the OLS estimation window for $\beta \in \{0.3,1,5,10\}$, under passive observation and impulse perturbation. (**e**) The same trajectories shown in full; the gold shade marks the OLS window and the dotted line marks the impulse time. (**f**) Held-out test trajectories (black) overlaid with the per-segment 1-step prediction from $\hat {\boldsymbol {A}},\hat {\boldsymbol {B}}$. (**g**) The same held-out test trajectories overlaid with the free-run (rollout) prediction. (**h**) The OLS-window data in the top three principal components (independent principal component analysis (PCA) per cell; viridis = sample index).

**Appendix 2—figure 2.**
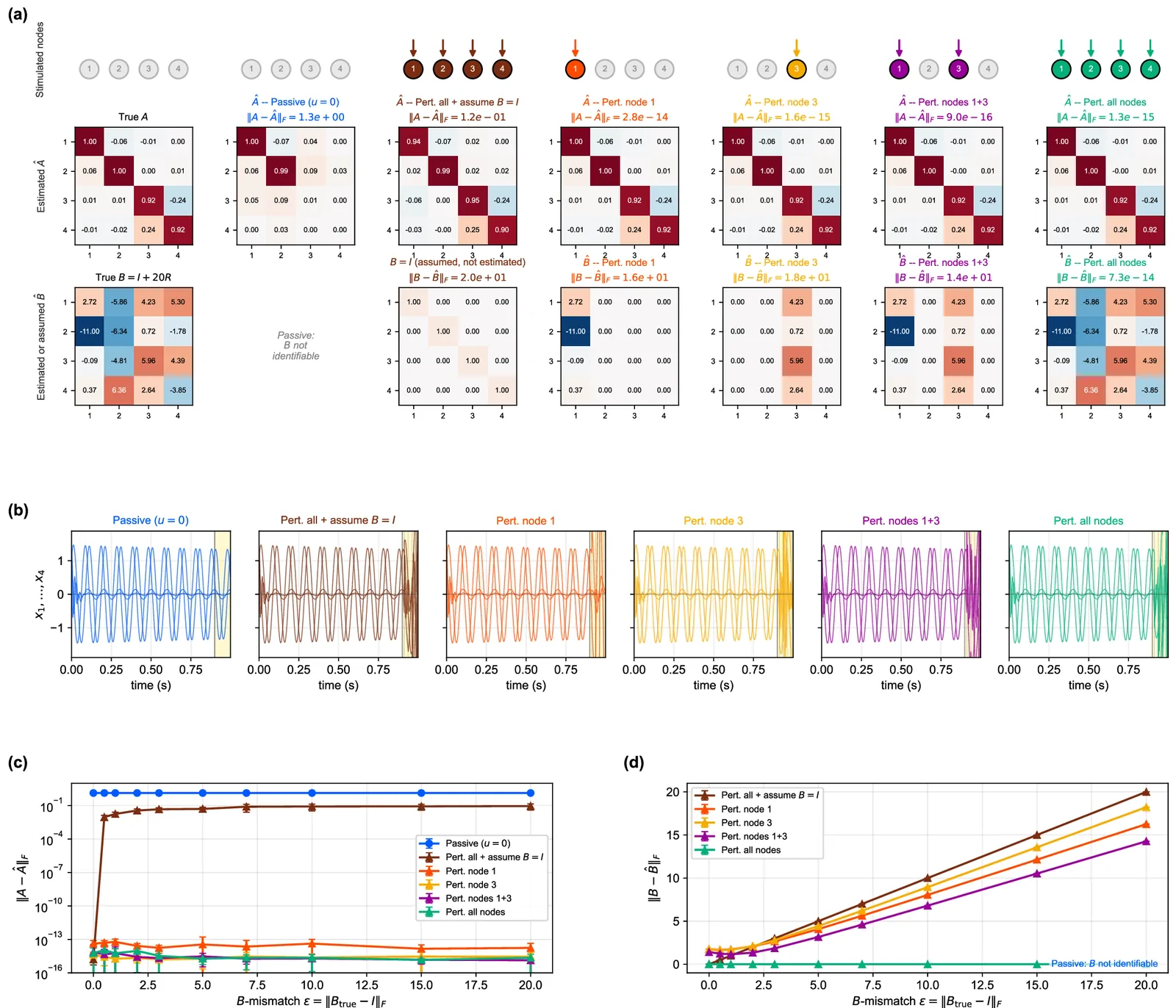
Perturbation framework can still improve the estimation of ***A*** even when ***B*** is unknown, provided that ***A*** and ***B*** are recovered jointly by ordinary least squares (OLS). (**a**) Top row: which channel(s) each condition stimulates (filled nodes are active; gray nodes are silent). Middle and bottom rows: recovered $\boldsymbol {A}$ and $\boldsymbol {B}$ at ε=20 from a single trial. Seven conditions are compared: ground truth, passive observation, a naive baseline that uses the all-nodes input but assumes $\hat {\boldsymbol {B}}=\boldsymbol {I}$, and joint estimation under four perturbation patterns (node 1 only, node 3 only, nodes 1+3, all nodes). (**b**) Corresponding sample state trajectories; the gold shade marks the OLS window and dotted lines mark the impulse times. (**c**) $\|\boldsymbol {A}-\hat {\boldsymbol {A}}\|_{F}$ as a function of *ε* (log scale). (**d**) $\|\boldsymbol {B}-\hat {\boldsymbol {B}}\|_{F}$ as a function of *ε*.

**Appendix 2—figure 3.**
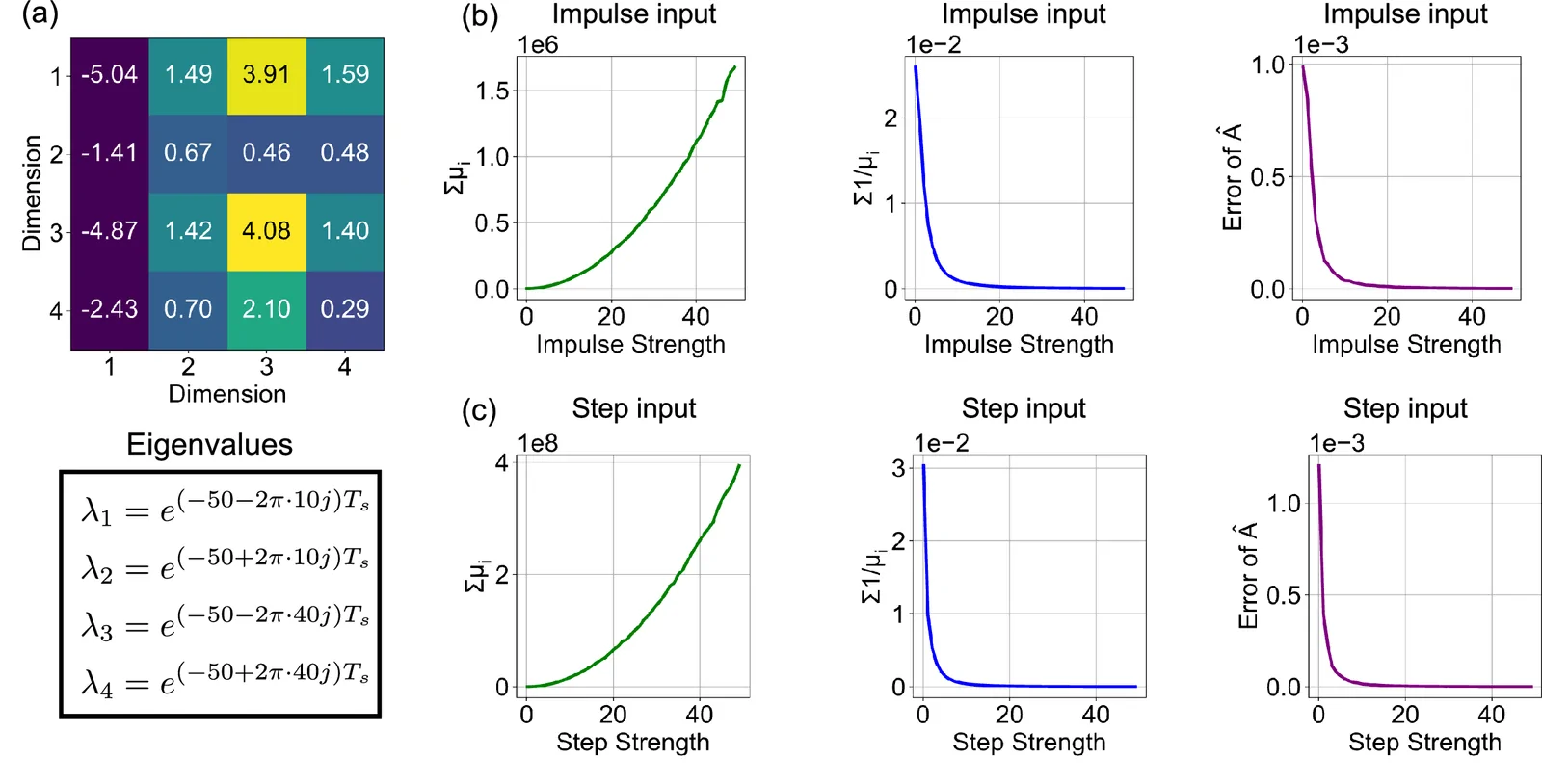
Variation of eigenvalues and system matrix error with impulse and step input strengths. (**a**) Assumed system matrix $\boldsymbol {A}$ and its eigenvalues. (**b**) Changes in the sum of eigenvalues, the sum of their reciprocals, and the estimation error of $\boldsymbol {A}$ with respect to impulse input strength. (**c**) Changes in the same quantities with respect to step input strength.

**Appendix 2—figure 4.**
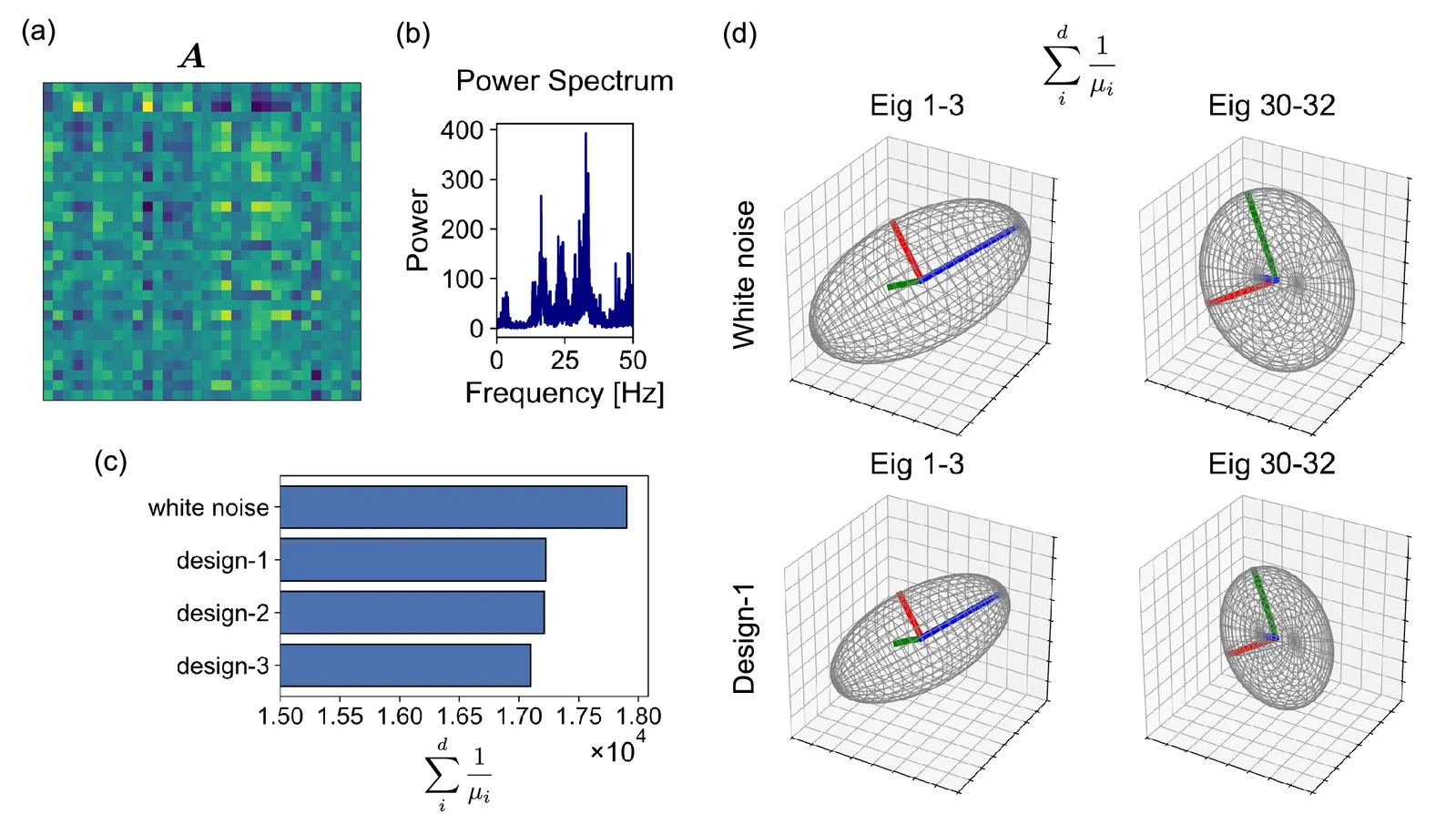
Comparison between white noise input and optimized frequency input. (**a**) Matrix A with 16 modes, assuming 32 channels. (**b**) Channel-averaged power spectrum in the passive state. (**c**) Comparison of the sum of reciprocal eigenvalues ($\sum _{i} (1/\mu _{i})$) of the covariance matrix of the state vector under white noise and optimized frequency input. (**d**) Comparison of eigenvalue-scaled ellipsoids obtained from white noise and optimized frequency input, visualizing the components corresponding to the 1^st^–3^rd^ and 30^th^–32^nd^ eigenvalues.

## Data Availability

The current manuscript is a computational study, so no biological data have been generated. The code for the simulations and analyses presented in this paper is openly accessible at https://github.com/mikito-ogino/NeuroPerturbID (copy archived at Ogino, 2026) released under a dual MIT or BSD 3-Clause licence.

## Additional files

MDAR checklist

## Appendix 1

### Theoretical details

#### 1.1 Asymptotic property of the estimator for *A*

This section derives the asymptotic estimation error of the OLS estimator $\hat {\boldsymbol {A}}$ for the system matrix ***A***. The primary goal is to obtain a first-order asymptotic approximation for the Mean Squared Error (MSE) of the estimation, quantified by the expected squared Frobenius norm, $\mathbb{E}[\|\hat {{A}}- {A}\|_{F}^{2}]$.

We consider the linear dynamical system

(A1.1) $$\displaystyle {\boldsymbol x}(t+1) = {\boldsymbol A} {\boldsymbol x}(t) + {\boldsymbol B} {\boldsymbol u}(t) + {\boldsymbol \xi }(t),$$

where the noise ${\boldsymbol \xi }(t)$ is i.i.d. over time with zero mean and covariance matrix $\boldsymbol {\Sigma }_{\xi } = \mathbb{E}[\boldsymbol {\xi }(t)\boldsymbol {\xi }(t)^{\!\top }] \in \mathbb{R}^{n \times n}\succ 0$.

We estimate ***A*** by OLS from the data matrices $\boldsymbol {X}\in \mathbb{R}^{n\times T}$, $\tilde {\boldsymbol {Y}}:= \boldsymbol {Y}- \boldsymbol {B}\boldsymbol {U}\in \mathbb{R}^{n\times T}$, giving $\hat {\boldsymbol {A}}= \tilde {\boldsymbol {Y}}\boldsymbol {X}^{\dagger }$.

The asymptotic distribution of the vectorized estimation error of the OLS estimator $\hat {\boldsymbol {A}}$ is given by Hamilton, 1994, Proposition 11.1 and Appendix 11.A:

(A1.2) $$\displaystyle \sqrt{T}\bigl (\mathrm{vec}(\hat {\boldsymbol {A}}) - \mathrm{vec}(\boldsymbol {A})\bigr) \xrightarrow {L}\mathcal{N}\bigl (\boldsymbol {0},\, \boldsymbol {\Sigma }_{\mathrm{Asym}}\bigr),$$

where *T* is the number of samples, $\xrightarrow {L}$ denotes convergence in distribution, and $\boldsymbol {\Sigma }_{\mathrm{Asym}}$ is the asymptotic covariance matrix. Following the notation in the original text (and its assumptions), this covariance is:

(A1.3) $$\displaystyle \boldsymbol {\Sigma }_{\mathrm{Asym}}= \boldsymbol {\Sigma }_{\xi } \otimes \boldsymbol {\Sigma }_{\boldsymbol {X}}^{-1},$$

where ⊗ denotes the Kronecker product [Hamilton, 1994, Appendix A.4, p.732], $\boldsymbol {\Sigma }_{\xi } \in \mathbb{R}^{n \times n}$ is the noise covariance, and $\boldsymbol {\Sigma }_{\boldsymbol {X}}= \mathbb{E}[\boldsymbol {x}(t)\boldsymbol {x}(t)^{\top }] \in \mathbb{R}^{n \times n}$ is the state covariance matrix, so that $\boldsymbol {\Sigma }_{\mathrm{Asym}}\in \mathbb{R}^{n^2 \times n^2}$.

We are interested in the estimation error measured by the squared Frobenius norm, $\|\hat {\boldsymbol {A}}- \boldsymbol {A}\|_{F}^{2}$. Let us define the normalized error vector $\boldsymbol {z}_{T}$ based on (Equation A1.2):

(A1.4) $$\displaystyle \boldsymbol {z}_{T} := \sqrt{T}\bigl (\mathrm{vec}(\hat {\boldsymbol {A}}) - \mathrm{vec}(\boldsymbol {A})\bigr).$$

We know that $\boldsymbol {z}_{T} \xrightarrow {L}\boldsymbol {z}$, where $\boldsymbol {z}\sim \mathcal{N}(\boldsymbol {0}, \boldsymbol {\Sigma }_{\mathrm{Asym}})$ is an $n^{2}$-dimensional Gaussian vector.

The squared Frobenius norm can be expressed as the squared Euclidean norm of the vectorized error:

(A1.5) $$\displaystyle \|\hat {\boldsymbol {A}}- \boldsymbol {A}\|_{F}^{2} = \|\mathrm{vec}(\hat {\boldsymbol {A}}- \boldsymbol {A})\|_{2}^{2}.$$

Multiplying by *T*, we can relate this quantity to $\boldsymbol {z}_{T}$:

(A1.6) $$\displaystyle T \cdot \|\hat {\boldsymbol {A}}- \boldsymbol {A}\|_{F}^{2} = \left (\sqrt{T}\cdot \|\mathrm{vec}(\hat {\boldsymbol {A}}- \boldsymbol {A})\|_{2}\right)^{2} = \boldsymbol {z}_{T}^{\top } \boldsymbol {z}_{T}.$$

By the continuous mapping theorem, since $g(\boldsymbol {z}) = \boldsymbol {z}^{\top } \boldsymbol {z}$ is a continuous function, the convergence in distribution of $\boldsymbol {z}_{T}$ implies the convergence in distribution of $\boldsymbol {z}_{T}^{\top } \boldsymbol {z}_{T}$:

(A1.7) $$\displaystyle T \cdot \|\hat {\boldsymbol {A}}- \boldsymbol {A}\|_{F}^{2} \xrightarrow {L}\boldsymbol {z}^{\top } \boldsymbol {z}, \quad \mathrm{where}\quad \boldsymbol {z}\sim \mathcal{N}(\boldsymbol {0}, \boldsymbol {\Sigma }_{\mathrm{Asym}}).$$

Its limit of the expected value equals the expectation of the limiting distribution $\boldsymbol {z}^{\top } \boldsymbol {z}$:

(A1.8) $$\displaystyle \lim _{T \to \infty }\mathbb{E}\!\left [T \cdot \|\hat {\boldsymbol {A}}- \boldsymbol {A}\|_{F}^{2}\right ] = \mathbb{E}\!\left [\boldsymbol {z}^{\top } \boldsymbol {z}\right ]$$

(A1.9) $$\displaystyle = \mathbb{E}\!\left [\mathrm{tr}(\boldsymbol {z}\boldsymbol {z}^{\top })\right ]$$

(A1.10) $$\displaystyle = \mathrm{tr}\!\left (\mathbb{E}[\boldsymbol {z}\boldsymbol {z}^{\top }]\right).$$

By definition, $\mathbb{E}[\boldsymbol {z}\boldsymbol {z}^{\top }]$ is the covariance matrix of the limiting distribution *z*, which is $\boldsymbol {\Sigma }_{\mathrm{Asym}}$.

(A1.11) $$\displaystyle \lim _{T \to \infty }\mathbb{E}\!\left [T \cdot \|\hat {\boldsymbol {A}}- \boldsymbol {A}\|_{F}^{2}\right ] = \mathrm{tr}(\boldsymbol {\Sigma }_{\mathrm{Asym}})$$

(A1.12) $$\displaystyle = \mathrm{tr}(\boldsymbol {\Sigma }_{\xi } \otimes \boldsymbol {\Sigma }_{\boldsymbol {X}}^{-1}).$$

Using the trace property $\mathrm{tr}(\boldsymbol {A}\otimes \boldsymbol {B}) = \mathrm{tr}(\boldsymbol {A})\,\mathrm{tr}(\boldsymbol {B})$, we obtain:

(A1.13) $$\displaystyle \lim _{T \to \infty }\mathbb{E}\!\left [T \cdot \|\hat {\boldsymbol {A}}- \boldsymbol {A}\|_{F}^{2}\right ] = \mathrm{tr}(\boldsymbol {\Sigma }_{\xi })\,\mathrm{tr}(\boldsymbol {\Sigma }_{\boldsymbol {X}}^{-1}).$$

This result implies that for a sufficiently large *T*, the expected estimation error can be approximated as:

(A1.14) $$\displaystyle \mathbb{E}\!\left [\|\hat {\boldsymbol {A}}- \boldsymbol {A}\|_{F}^{2}\right ] \approx \frac{1}{T}\,\mathrm{tr}(\boldsymbol {\Sigma }_{\xi })\,\mathrm{tr}(\boldsymbol {\Sigma }_{\boldsymbol {X}}^{-1}).$$

This expression provides a simple interpretation of the estimation error. It confirms that the error is inversely proportional to the sample size *T*, meaning the error decreases as more data is collected. The error is also directly proportional to the total noise power, $\mathrm{tr}(\boldsymbol {\Sigma }_{\xi })$, indicating that more noise leads to more error. Furthermore, the error is proportional to $\mathrm{tr}(\boldsymbol {\Sigma }_{\boldsymbol {X}}^{-1})$, which acts as an inverse measure of signal strength; a weaker signal $\boldsymbol {x}(t)$ (i.e. less variation) leads to a larger error. This relationship resembles the inverse of the Signal-to-Noise ratio, where the error is governed by the balance between noise, signal strength, and the amount of data.

#### 1.2 State vectors considering typical inputs used in neuroscience

##### 1.2.1 Sinusoidal Input

To determine which frequencies most effectively enhance system identification accuracy, we now analyze the time evolution of the state vector $\boldsymbol {x}(t)$ in response to a sinusoidal input. This analysis is the necessary first step towards the goal established in the main text (i.e. sufficiently exciting the system’s dynamics to increase the eigenvalues of the covariance matrix). By understanding how $\boldsymbol {x}(t)$ itself behaves under different frequencies, we can identify which inputs are most effective. We, therefore, examine frequency external inputs, represented in neuroscience by tACS, to derive the optimal input frequency based on the system’s dynamic response.

In the case where the perturbation consists of a composite sinusoidal input comprising multiple frequencies, the input can be written as

(A1.15) $$\displaystyle  \boldsymbol {u}(k) = \sum _{l=1}^{L}\cos (\omega _{l} k)\boldsymbol {u}_{0},$$

where $\omega _{l}$ are the angular frequencies.

The general solution which we denote as Equation 10 is:

(A1.16) $$\displaystyle  \boldsymbol {x}(t) = \boldsymbol {A}^{t} \boldsymbol {x}(0) + \sum _{k=0}^{t-1}\boldsymbol {A}^{t-1-k}\boldsymbol {B}\boldsymbol {u}(k) + \sum _{k=0}^{t-1}\boldsymbol {A}^{t-1-k}\boldsymbol {\xi }(k)$$

Inserting Equation A1.15 into Equation 10 yields:

(A1.17) $$\displaystyle  \boldsymbol {x}(t) = \boldsymbol {A}^{t} \boldsymbol {x}(0) + \sum _{k=0}^{t-1}\boldsymbol {A}^{t-1-k}\boldsymbol {B}\sum _{l=1}^{L}\cos (\omega _{l} k)\boldsymbol {u}_{0} + \sum _{k=0}^{t-1}\boldsymbol {A}^{t-1-k}\boldsymbol {\xi }(k)$$

Under the assumption that the system is stable (all eigenvalues $\lambda _{d}$ of ***A*** satisfy $|\lambda _{d}| < 1$), the second term of Equation A1.17 is the critical component to analyze.

By performing diagonalization of ***A*** as $\boldsymbol {A}= \boldsymbol {V}\boldsymbol {\Lambda }\boldsymbol {V}^{-1}$, where ***V*** is the matrix of eigenvectors, $\boldsymbol {\Lambda }$ is the diagonal matrix of eigenvalues $\lambda _{d}$,... The spectral decomposition of $\boldsymbol {A}^{m}$ is $\boldsymbol {A}^{m} = \sum _{d=1}^{n} \lambda _{d}^{m} \boldsymbol {v}_{d} \boldsymbol {w}_{d}^{\top }$, where $\boldsymbol {v}_{d}$ denotes the *d*-th column vector of ***V***, and $\boldsymbol {w}_{d}^{\top }$ denotes the *d*-th row vector of $\boldsymbol {V}^{-1}$. The second term of Equation A1.17 can be expressed as follows:

(A1.18) $$\displaystyle  \sum _{k=0}^{t-1}\boldsymbol {A}^{\,t-1-k}\boldsymbol {B}\,\sum _{l=1}^{L}\cos (\omega _{l} k)\boldsymbol {u}_{0}$$

(A1.19) $$\displaystyle  = \sum _{k=0}^{t-1}\boldsymbol {V}\boldsymbol {\Lambda }^{\,t-1-k}\boldsymbol {V}^{-1}\boldsymbol {B}\,\sum _{l=1}^{L}\cos (\omega _{l} k)\boldsymbol {u}_{0}$$

(A1.20) $$\displaystyle  = \sum _{k=0}^{t-1}\sum _{d=1}^{n}\lambda _{d}^{\,t-1-k}\,\boldsymbol {v}_{d} \boldsymbol {w}_{d}^{\top } \boldsymbol {B}\,\sum _{l=1}^{L}\cos (\omega _{l} k)\boldsymbol {u}_{0}$$

Using Euler’s formula ($\cos x = (e^{ix}+ e^{-ix})/2$) and swapping the order of summation (which is permissible by linearity), we get:

(A1.21) $$\displaystyle  \sum _{k=0}^{t-1}\sum _{d=1}^{n}\lambda _{d}^{\,t-1-k}\,\boldsymbol {v}_{d} \boldsymbol {w}_{d}^{\top } \boldsymbol {B}\,\sum _{l=1}^{L}\cos (\omega _{l} k)\boldsymbol {u}_{0}$$

(A1.22) $$\displaystyle  = \sum _{k=0}^{t-1}\sum _{d=1}^{n}\sum _{l=1}^{L}\frac{e^{i\omega _l k}+e^{-i \omega _l k}}{2}\,\lambda _{d}^{\,t-1-k}\,\boldsymbol {v}_{d} \boldsymbol {w}_{d}^{\top } \boldsymbol {B}\,\boldsymbol {u}_{0}$$

(A1.23) $$\displaystyle  = \frac{1}{2}\sum _{l=1}^{L}\sum _{d=1}^{n}\left (\sum _{k=0}^{t-1}\lambda _{d}^{\,t-1-k}e^{i\omega _l k}+\sum _{k=0}^{t-1}\lambda _{d}^{\,t-1-k}e^{-i \omega _l k}\right) \boldsymbol {v}_{d} \boldsymbol {w}_{d}^{\top } \boldsymbol {B}\,\boldsymbol {u}_{0}$$

We now introduce the polar form of the eigenvalues, $\lambda _{d} = r_{d} e^{i\theta _d}$. Given the stability assumption, we have $r_{d} = |\lambda _{d}| < 1$. This yields:

(A1.24) $$\displaystyle  = \frac{1}{2}\sum _{l=1}^{L}\sum _{d=1}^{n}\left (\frac{\lambda _{d}^{\,t}-e^{i\omega _l t}}{\lambda _{d}-e^{i\omega _l}}+\frac{\lambda _{d}^{\,t}-e^{-i \omega _l t}}{\lambda _{d}-e^{-i \omega _l}}\right)\boldsymbol {v}_{d} \boldsymbol {w}_{d}^{\top } \boldsymbol {B}\,\boldsymbol {u}_{0}$$

(A1.25) $$\displaystyle  = \frac{1}{2}\sum _{l=1}^{L}\sum _{d=1}^{n}\left (\frac{r_{d}^{\,t}e^{i\theta _dt}-e^{i\omega _l t}}{r_{d}e^{i\theta _d}-e^{i\omega _l}}+\frac{r_{d}^{\,t}e^{i\theta _dt}-e^{-i \omega _l t}}{r_{d}e^{i\theta _d}-e^{-i \omega _l}}\right) \boldsymbol {v}_{d} \boldsymbol {w}_{d}^{\top } \boldsymbol {B}\,\boldsymbol {u}_{0}$$

(A1.26) $$\displaystyle  = \frac{1}{2}\sum _{l=1}^{L}\sum _{d=1}^{n}\left (\frac{(r_{d}^{\,t}e^{i\theta _dt}-e^{i\omega _l t})(r_{d}e^{-i\theta _d}-e^{-i\omega _l})}{(r_{d}e^{i\theta _d}-e^{i\omega _l})(r_{d}e^{-i\theta _d}-e^{-i\omega _l})}+\frac{(r_{d}^{\,t}e^{i\theta _dt}-e^{-i \omega _l t})(r_{d}e^{-i\theta _d}-e^{i \omega _l})}{(r_{d}e^{i\theta _d}-e^{-i \omega _l})(r_{d}e^{-i\theta _d}-e^{i \omega _l})}\right) \boldsymbol {v}_{d} \boldsymbol {w}_{d}^{\top } \boldsymbol {B}\,\boldsymbol {u}_{0}$$

(A1.27) $$\displaystyle  \begin{array}{ll} =\frac{1}{2}\sum _{l=1}^{L}\sum _{d=1}^{n}\left (\frac{r_{d}^{t+1}e^{i\theta _d(t-1)}-r_{d}^{t}e^{i(\theta _dt-\omega _l)}-r_{d}e^{i(\omega _l t-\theta _d)}+e^{i\omega _l(t-1)}}{r_{d}^{2}-2r_{d}\cos (\theta _{d}-\omega _{l})+1}\right . \notag\\\hspace{12em}\left . +\frac{r_{d}^{t+1}e^{i\theta _d(t-1)}-r_{d}^{t}e^{i(\theta _dt+\omega _l)}-r_{d}e^{i(-\omega _l t-\theta _d)}+e^{-i\omega _l(t-1)}}{r_{d}^{2}-2r_{d}\cos (\theta _{d}+\omega _{l})+1}\right) \boldsymbol {v}_{d} \boldsymbol {w}_{d}^{\top } \boldsymbol {B}\,\boldsymbol {u}_{0},\end{array}$$

For brevity, we define the following terms:

(A1.28) $$\displaystyle Q(\omega _{l}, \theta _{d})= \frac{r_{d}^{t+1}e^{i\theta _d(t-1)}-r_{d}^{t}e^{i(\theta _dt-\omega _l)}-r_{d}e^{i(\omega _l t-\theta _d)}+e^{i\omega _l(t-1)}}{r_{d}^{2}-2r_{d}\cos (\theta _{d}-\omega _{l})+1}$$

(A1.29) $$\displaystyle \boldsymbol {F}_{d} = \boldsymbol {v}_{d}\boldsymbol {w}_{d}^{\top }\boldsymbol {B}\boldsymbol {u}_{0}$$

The second term is:

(A1.30) $$\displaystyle  \sum_{k=0}^{t-1}\boldsymbol{A}^{\,t-1-k}\boldsymbol{B}\,\sum_{l=1}^{L}\cos(\omega_l k)\boldsymbol{u}_0 = \frac{1}{2}\sum_{l=1}^{L}\sum_{d=1}^{n}\left(Q(\omega_l,\theta_d)+Q(-\omega_l,\theta_d)\right)\boldsymbol{F}_d$$

Since the matrix ***A*** is real, its complex eigenvalues and eigenvectors must appear in complex conjugate pairs. If $\lambda _{d} = r_{d} e^{i\theta _d}$ (with $\theta _{d} \neq 0$) is an eigenvalue, then $\lambda _{d'}= \overline {\lambda _d}= r_{d} e^{-i\theta _d}$ is also an eigenvalue for some index $d^{\prime }$. Thus, $\theta _{d'}= -\theta _{d}$. Furthermore, since ***B*** and $\boldsymbol {u}_{0}$ are also real, the vector $\boldsymbol {F}_{d} = \boldsymbol {v}_{d}\boldsymbol {w}_{d}^{\top }\boldsymbol {B}\boldsymbol {u}_{0}$ associated with $\lambda _{d}$ and the vector $\boldsymbol {F}_{d'}= \boldsymbol {v}_{d'}\boldsymbol {w}_{d'}^{\top }\boldsymbol {B}\boldsymbol {u}_{0}$ associated with $\lambda _{d'}$ are also complex conjugates: $\boldsymbol {F}_{d'}= \overline {\boldsymbol {F}_d}$. We can use this property to simplify the sum.

(A1.31) $$\displaystyle Q(-\omega _{l}, \theta _{d'}) \boldsymbol {F}_{d'} = Q(-\omega _{l},-\theta _{d})\overline {\boldsymbol {F}_d}$$

(A1.32) $$\displaystyle = \frac{r_{d}^{t+1}e^{-i\theta _d(t-1)}-r_{d}^{t}e^{i(-\theta _dt+\omega _l)}-r_{d}e^{i(-\omega _l t+\theta _d)}+e^{-i\omega _l(t-1)}}{r_{d}^{2}-2r_{d}\cos (-\theta _{d}+\omega _{l})+1}\overline {\boldsymbol {F}_d}$$

(A1.33) $$\displaystyle = \overline {\frac{r_{d}^{t+1}e^{i\theta _d(t-1)}-r_{d}^{t}e^{i(\theta _dt-\omega _l)}-r_{d}e^{i(\omega _l t-\theta _d)}+e^{i\omega _l(t-1)}}{r_{d}^{2}-2r_{d}\cos (\theta _{d}-\omega _{l})+1}\boldsymbol {F}_d}$$

(A1.34) $$\displaystyle = \overline {Q(\omega _l, \theta _{d})\boldsymbol {F}_d}$$

Similarly, $Q(-\omega _{l}, \theta _{d})\boldsymbol {F}_{d} = \overline {Q(\omega _l, \theta _{d'})\boldsymbol {F}_{d'}}$

For real eigenvalues ($\theta _{d}=0$), $\boldsymbol {F}_{d}$ is real, and we find:

(A1.35) $$\displaystyle Q(-\omega _{l}, 0) \boldsymbol {F}_{d} = \frac{r_{d}^{t+1}-r_{d}^{t}e^{i\omega _l}-r_{d}e^{-i\omega _l t}+e^{-i\omega _l(t-1)}}{r_{d}^{2}-2r_{d}\cos (\omega _{l})+1}\boldsymbol {F}_{d}$$

(A1.36) $$\displaystyle = \overline {\frac{r_{d}^{t+1}-r_{d}^{t}e^{-i\omega _l}-r_{d}e^{i\omega _l t}+e^{i\omega _l(t-1)}}{r_{d}^{2}-2r_{d}\cos (\omega _{l})+1}\boldsymbol {F}_{d}}$$

(A1.37) $$\displaystyle = \overline {Q(\omega _l, 0) \boldsymbol {F}_{d}}$$

We divide the set of indices of eigenvalues into three subsets as follows. Let $D_{R}$ denote the set of indices corresponding to real eigenvalues ($\lambda _{d} \in \mathbb{R}$). Among complex eigenvalues, let $D_{C}^{+}$ denote the set of indices whose imaginary part is positive, and let $D_{C}^{-}$ denote the set of indices whose imaginary part is negative.

(A1.38) $$\displaystyle  \frac{1}{2}\sum _{d=1}^{n}\left (Q(\omega _{l}, \theta _{d}) + Q(-\omega _{l}, \theta _{d})\right) \boldsymbol {F}_{d} = \frac{1}{2}\sum _{d \in D_R}\left (Q(\omega _{l}, 0)\boldsymbol {F}_{d} + \overline {Q(\omega _l, 0)\boldsymbol {F}_d}\right)$$

(A1.39) $$\displaystyle  + \frac{1}{2}\sum _{d \in D_C^+}\left (Q(\omega _{l}, \theta _{d})\boldsymbol {F}_{d} + Q(-\omega _{l}, \theta _{d})\boldsymbol {F}_{d} \right)$$

(A1.40) $$\displaystyle  + \frac{1}{2}\sum _{d' \in D_C^-}\left (Q(\omega _{l}, \theta _{d'})\boldsymbol {F}_{d'}+ Q(-\omega _{l}, \theta _{d'})\boldsymbol {F}_{d'}\right)$$

Now, we pair the terms from $D_{C}^{+}$ and $D_{C}^{-}$. For each $d \in D_{C}^{+}$, there is a corresponding $d' \in D_{C}^{-}$ such that $\theta _{d'}= -\theta _{d}$ and $\boldsymbol {F}_{d'}= \overline {\boldsymbol {F}_d}$. Using the properties $Q(-\omega _{l}, \theta _{d})\boldsymbol {F}_{d} = \overline {Q(\omega _l, \theta _{d'})\boldsymbol {F}_{d'}}$ and $Q(-\omega _{l}, \theta _{d'})\boldsymbol {F}_{d'}= \overline {Q(\omega _l, \theta _d)\boldsymbol {F}_d}$, the sum of the $D_{C}^{+}$ and $D_{C}^{-}$ terms becomes:

(A1.41) $$\displaystyle  \frac{1}{2}\sum _{d \in D_C^+}\left (Q(\omega _{l}, \theta _{d})\boldsymbol {F}_{d} + \overline {Q(\omega _l, \theta {d'})\boldsymbol {F}_{d'}}+ Q(\omega _{l}, \theta {d'})\boldsymbol {F}_{d'}+ \overline {Q(\omega _l, \theta _d)\boldsymbol {F}_d}\right)$$

(A1.42) $$\displaystyle  = \frac{1}{2}\sum _{d \in D_C^+}\left ((Q_{d}\boldsymbol {F}_{d} + \overline {Q_d\boldsymbol {F}_d}) + (Q{d'}\boldsymbol {F}_{d'}+ \overline {Q{d'}\boldsymbol {F}_{d'}}) \right)$$

(A1.43) $$\displaystyle  = \sum _{d \in D_C^+}\mathrm{Re}\left [Q(\omega _{l}, \theta _{d})\boldsymbol {F}_{d}\right ] + \sum _{d' \in D_C^-}\mathrm{Re}\left [Q(\omega _{l}, \theta _{d'})\boldsymbol {F}_{d'}\right ]$$

Combining this with the $D_{R}$ sum, which is $\sum _{d \in D_R}\mathrm{Re}[Q(\omega _{l}, 0)\boldsymbol {F}_{d}]$, we arrive at:

(A1.44) $$\displaystyle  \sum _{d=1}^{n}\mathrm{Re}\left [Q(\omega _{l}, \theta _{d})\boldsymbol {F}_{d}\right ]$$

This is the real part of the product of the terms $Q(\omega _{l}, \theta _{d})$ and $\boldsymbol {F}_{d}$ defined in Equations A1.28; A1.29. Therefore, the second term of the $\boldsymbol {x}(t)$ can be expressed as:

(A1.45) $$\displaystyle  \sum _{k=0}^{t-1}\boldsymbol {A}^{\,t-1-k}\boldsymbol {B}\,\sum _{l=1}^{L}\cos (\omega _{l} k)\boldsymbol {u}_{0}$$

(A1.46) $$\displaystyle  = \sum _{l=1}^{L}\sum _{d=1}^{n}\mathrm{Re}\left [\frac{r_{d}^{t+1}e^{i\theta _d(t-1)}-r_{d}^{t}e^{i(\theta _dt-\omega _l)}-r_{d}e^{i(\omega _l t-\theta _d)}+e^{i\omega _l(t-1)}}{r_{d}^{2}-2r_{d}\cos (\theta _{d}-\omega _{l})+1}\boldsymbol {v}_{d} \boldsymbol {w}_{d}^{\top } \boldsymbol {B}\,\boldsymbol {u}_{0}\right ],$$

Therefore, the state vector $\boldsymbol {x}(t)$ is decomposed into a passive and a difference term as follows,

(A1.47) $$\displaystyle \boldsymbol {x}(t) = \boldsymbol {x}_{\mathrm{passive}}(t)+\boldsymbol {x}_{\mathrm{diff}}(t),$$

(A1.48) $$\displaystyle  \boldsymbol {x}_{\mathrm{passive}}(t) = \boldsymbol {A}^{t} \boldsymbol {x}(0) + \sum _{k=0}^{t-1}\boldsymbol {A}^{t-1-k}\boldsymbol {\xi }(k)$$

(A1.49) $$\displaystyle  \boldsymbol {x}_{\mathrm{diff}}(t) = \sum _{l=1}^{L}\sum _{d=1}^{n}\mathrm{Re}\left [\frac{r_{d}^{t+1}e^{i\theta _d(t-1)}-r_{d}^{t}e^{i(\theta _dt-\omega _l)}-r_{d}e^{i(\omega _l t-\theta _d)}+e^{i\omega _l(t-1)}}{r_{d}^{2}-2r_{d}\cos (\theta _{d}-\omega _{l})+1}\boldsymbol {v}_{d} \boldsymbol {w}_{d}^{\top } \boldsymbol {B}\,\boldsymbol {u}_{0}\right ]$$

where $\boldsymbol {x}_{\mathrm{passive}}(t)$ represents the state *x* that would be realized if no perturbation were applied, i.e., the state that would evolve according to Equation 10 with $\boldsymbol {u}=0$ in the main text. $\boldsymbol {x}_{\mathrm{diff}}(t)$ represents the change in the state trajectory induced by the specific perturbation input $\boldsymbol {u}(k) = \sum _{l=1}^{L}\cos (\omega _{l} k)\boldsymbol {u}_{0}$. As defined in the previous section, $\lambda _{d} = r_{d} e^{i\theta _d}$ is the polar form of the *d*-th eigenvalue, the vector $\boldsymbol {v}_{d}$ denotes the right eigenvector of ***A*** associated with $\lambda _{d}$, and $\boldsymbol {w}_{d}^{\top }$ denotes the corresponding left eigenvector. The numerator of the fraction in Equation A1.46 consists of four distinct oscillatory terms. The resonant behavior of this expression is primarily governed by the denominator, $D(\omega _{l}, \theta _{d}) = r_{d}^{2}-2r_{d}\cos (\theta _{d}-\omega _{l})+1$. The gain term $1/D(\omega _{l}, \theta _{d})$ has a single extremum (a maximum) with respect to $\omega _{l}$, which occurs when $\cos (\theta _{d}-\omega _{l})=1$, i.e., when the input frequency $\omega _{l}$ matches the eigenfrequency (argument) $\theta _{d}$ of the system.

The restriction to a common spatial pattern $\boldsymbol {u}_{0}$ in the input $\boldsymbol {u}(k) = \sum _{l=1}^{L} \cos (\omega _{l} k)\,\boldsymbol {u}_{0}$ is a simplifying assumption made to present the theory of this paper clearly, and it can be naturally replaced by frequency-specific patterns $\boldsymbol {u}_{l}$. The simplification keeps the formulation consistent with the impulse and step inputs, and it isolates the effect of the input frequencies from that of the stimulation location, thereby keeping the presentation in the main text intuitive. Substituting $\boldsymbol {u}_{0} \to \boldsymbol {u}_{l}$ gives a more general expression, but it leaves the denominator $D(\omega _{l}, \theta _{d})$ untouched, so the conclusion that the input frequencies should match the mode frequencies is unchanged. Optimization that also includes the stimulation location can be carried out by numerically minimizing Equation A1.14, so the practical estimation procedure and design strategy remain unchanged.

##### Appendix 1.2.2 Impulse and step input

Impulse and step inputs follow simple rules. Let *α* represent the scaling factor for impulse input. Then, input $\boldsymbol {u}(k)$ is defined as:

(A1.50) $$\displaystyle \boldsymbol {u}(k) = \begin{cases} \alpha \boldsymbol {u}_{0}&\text{if }k = 0, \\ 0&\text{if }k > 0. \end{cases}$$

Here, $\boldsymbol {u}_{0} \in \mathbb{R}^{m}$ represents the fundamental input direction applied to the system, determining which input channel receives the impulse. We obtain the decomposition:

(A1.51) $$\displaystyle  \boldsymbol {x}_{\mathrm{passive}}(t) = \boldsymbol {A}^{t}\boldsymbol {x}(0)+\sum _{k=0}^{t-1}\boldsymbol {A}^{t-1-k}\boldsymbol {\xi }(k),\quad \boldsymbol {x}_{\mathrm{diff}}(t) = \alpha \boldsymbol {A}^{t-1}\boldsymbol {B}\boldsymbol {u}_{0}.$$

For a scaled step input, $\boldsymbol {u}(k)$ is defined as

(A1.52) $$\displaystyle \boldsymbol {u}(k) = \beta \boldsymbol {u}_{0}, \quad \forall k \geq 0,$$

where *β* is the input strength. We then get the decomposition:

(A1.53) $$\displaystyle  \boldsymbol {x}_{\mathrm{passive}}(t) = \boldsymbol {A}^{t}\boldsymbol {x}(0)+\sum _{k=0}^{t-1}\boldsymbol {A}^{t-1-k}\boldsymbol {\xi }(k),\quad \boldsymbol {x}_{\mathrm{diff}}(t) = \beta \sum _{k=0}^{t-1}\boldsymbol {A}^{t-1-k}\boldsymbol {B}\boldsymbol {u}_{0}.$$

From these solutions, we can infer that the input-driven terms ($\boldsymbol {x}_{\mathrm{diff}}(t)$) are linear in the input strengths *α* and *β*. Consequently, the contribution of these inputs to the state covariance matrix (i.e. the outer product of the state vector) will scale quadratically with $\alpha ^{2}$ and $\beta ^{2}$. This analysis, which suggests that such inputs can increase the rank or magnitude of the covariance matrix eigenvalues, is explored further in the main text.

## Appendix 2

### Experimental conditions and results

#### 2.1 Simulation details

The specific conditions used for generating neural dynamics in each section are summarized in Appendix 2—table 1. These include factors, such as sampling frequency, trial length, and perturbation input strength, which are crucial for designing reproducible simulations and evaluations. These parameters are chosen to illustrate the theoretical insights as clearly as possible, rather than to characterise the framework’s behaviour across generic regimes.

**Appendix 2—table 1.** Conditions for generating neural dynamics.

Section	Sampling frequency	Time length	Data length *T*	Number of trials	Noise	Input strength
Figure 2 (Failure of passive estimation)	1000	10	10,000	100	10^−32^	1
Figures 4 and 5 (Sinusoidal inputs)	100	50	5000	10,000	10^−2^	1
Figure 6 (Location tuning)	1000	1	1000	200	10^−2^	10
Figure 7 (Neural state classification)	10	2	20	100	10^−2^	10^2^
Figure 9 (Optimal control)	50	1	50	100	10^−2^	10^3^
Figure 10 (Practical designing procedure)	100	25	2500	500	10^−2^	0.1
Appendix 2—figure 1 (Nonlinear dynamics)	1000	1	1000	30	10^−32^	0.15–0.3
Appendix 2—figure 2(***B***-Matrix uncertainty)	1000	1	1000	30	10^−32^	0.15–0.3
Appendix 2—figure 3 (Impulse/step)	100	50	5000	1	10^−2^	0.5–5
Appendix 2—figure 4 (White noise/designed)	100	100	10,000	1	10^−2^	0.1

#### 2.2 Robustness to nonlinear state dynamics

We introduce nonlinearity into the state update by replacing the linear update with a nonlinear function of the current state,

(A2.1) $$\displaystyle \boldsymbol {x}(t{+}1)=\frac{\boldsymbol {A}}{\beta }\,\tanh \!\bigl (\beta \,\boldsymbol {x}(t)\bigr)+\boldsymbol {B}\boldsymbol {u}(t)+\boldsymbol {\xi }(t),$$

on a 4-dimensional two-mode network, which is the extended setup of Figure 2. The detailed parameters are given in Appendix 2—table 1. The 1/β prefactor normalises the dynamics. The tanh function is a common choice for introducing nonlinearity (Khalil, 2002) while maintaining bounded outputs, and the parameter *β* controls the degree of nonlinearity (Appendix 2—figure 1a). Because the true map is now nonlinear in *x*, a direct comparison of $\hat {\boldsymbol {A}}$ against the true ***A*** is no longer well-defined; we, therefore, evaluate the misspecified linear OLS estimator by the relative one-step prediction RMSE and the relative free-run (rollout) RMSE.

Under both metrics, the impulse condition tracks the true dynamics more accurately than passive observation across the swept range, with the gap shrinking when *β* grows large enough that the nonlinearity itself re-excites the 40 Hz hidden mode strongly (Appendix 2—figure 1b and c). Appendix 2—figure 1d and e show the time series for the passive and impulse conditions across the range of *β*, illustrating that the impulse condition strongly excites the mode dynamics. Appendix 2—figure 1f and g compare the true and predicted time series; under both error metrics, the impulse condition tracks the true dynamics more accurately than passive observation. Appendix 2—figure 1h shows the top three principal components of the data. Even when nonlinearity is present in the dynamics, the impulse condition still re-excites the hidden mode (third PC) and yields better predictions than passive observation.

#### 2.3 Joint estimation of *A* and *B*

The naive estimator that assumes $\boldsymbol {B}=\boldsymbol {I}$ absorbs the unmodelled term $(\boldsymbol {B}_{\mathrm{true}}-\boldsymbol {I})\boldsymbol {u}$ into $\hat {\boldsymbol {A}}$, so its error in *A* grows with the mismatch level $\varepsilon =\|\boldsymbol {B}_{\mathrm{true}}-\boldsymbol {I}\|_{F}$. A natural remedy is to estimate *A* and *B* jointly by regressing the next state on the stacked $[\boldsymbol {X};\boldsymbol {U}]$ block,

(A2.2) $$\displaystyle [\hat {\boldsymbol {A}},\,\hat {\boldsymbol {B}}] \;=\; \boldsymbol {X}_{+}\;[\boldsymbol {X}_{-};\,\boldsymbol {U}]^{\dagger },$$

which is unbiased whenever the input excites every state coordinate. We compare seven conditions on a 4-dimensional, 2-mode discrete-time system: the ground truth; passive observation; joint estimation under four perturbation patterns, with input on (i) node 1 alone, (ii) node 3 alone, (iii) nodes 1 and 3, and (iv) all four nodes; and a baseline that uses the all-nodes input but estimates ***A*** under the naive assumption $\hat {\boldsymbol {B}}=\boldsymbol {I}$. The true matrix uses the same setup as Figure 2. The detailed parameters are given in Appendix 2—table 1. Every stimulated trial uses 4 impulses whose amplitudes on the active channels are drawn independently from $\mathcal{N}(0,\boldsymbol {I})$ per pulse, so that the rank of the ***U***-block of the regressor equals $\min (n_{\mathrm{active}}, 4)$. We parametrise the mismatch as $\boldsymbol {B}_{\mathrm{true}}=\boldsymbol {I}+\varepsilon \boldsymbol {R}$ with a fixed random matrix ***R*** normalised so that $\|\boldsymbol {R}\|_{F}=1$, which makes *ε* the scalar control parameter for the sweep.

The results show that the perturbation can still improve the estimation of ***A*** even when ***B*** is unknown, provided that ***A*** and ***B*** are recovered jointly by OLS. In the joint-estimation results of Appendix 2—figure 2a, only the columns of ***B*** corresponding to the stimulated channels are recovered: the (1, 3) pattern fills two columns, and the all-nodes pattern recovers ***B*** to the accuracy attainable when ***B*** is known. Nevertheless, ***A*** can be estimated correctly, including its hidden dynamical mode. The third column of the same panel shows that the naive estimator under the assumption $\hat {\boldsymbol {B}}=\boldsymbol {I}$ recovers ***A*** only approximately. Appendix 2—figure 2b shows the corresponding state trajectories. Appendix 2—figure 2c shows $\|\boldsymbol {A}-\hat {\boldsymbol {A}}\|_{F}$ as *ε* is swept on a log scale. Passive observation is pinned at the hidden-mode bias, and the naive $\hat {\boldsymbol {B}}=\boldsymbol {I}$ baseline grows with *ε* as the unmodelled $(\boldsymbol {B}_{\mathrm{true}}-\boldsymbol {I})\boldsymbol {u}_{t}$ term inflates the residual variance. Among the joint estimators, stimulating node 1 alone yields an intermediate error because node 1 is not directly coupled to the hidden mode; stimulating node 3 yields a markedly lower error because node 3 excites the hidden mode and thereby renders it identifiable; and stimulating all nodes is better still, matching the error achievable when ***B*** is known. The (1, 3) pattern lies between the node 3 and all-nodes cases. The error $\|\boldsymbol {B}-\hat {\boldsymbol {B}}\|_{F}$ grows linearly with *ε* for the rank-deficient stimulation patterns and the naive baseline, and matches the known-***B*** baseline only for the all-nodes pattern under joint OLS. Although these results provide only a preliminary demonstration of the robustness of the proposed framework, joint estimation of ***A*** and ***B*** is a promising direction for future work to relax the assumption of known ***B***.

#### 2.4 Demonstration of impulse and step input

Impulse- and step-like inputs, such as those applied in TMS or tDCS, can substantially improve system identification accuracy when applied at high intensities. By perturbing the system state further from equilibrium, the resulting decay dynamics provide richer trajectory information. This richer information increases the eigenvalues of the state covariance matrix and, in turn, reduces estimation error.

The relationship between input intensity and system identification accuracy was examined through simulations using matrix ***A***, as illustrated in Appendix 2—figure 3a. For the dynamics generated by matrix ***A***, system identification was performed using impulse and step inputs of varying intensities. As shown in Appendix 2—figure 3b and c, the identification error decreases exponentially as the input intensity increases. This error was observed to be inversely correlated with the sum of the inverses of the eigenvalues of the state covariance matrix. In practice, the strength of perturbation input must be constrained within the limits allowed by experimental conditions. However, our findings indicate that perturbation inputs should be designed to be as strong as possible within biologically acceptable ranges.

#### 2.5 White noise vs. concatenated frequency input

We demonstrate both the effectiveness and suboptimality of white noise input for neural system identification, a standard approach widely used in control engineering (Goodwin and Payne, 1977; Ljung, 1999). White noise contains a broad and nearly uniform range of frequencies, making it a convenient and practical choice for identifying system models when no prior information is available. However, as discussed in the main text, the input should ideally be designed to match the dynamical modes of the system. Thus, we consider white noise input as a practical yet potentially suboptimal method for neural system identification.

To assess the feasibility of the white noise perturbation input, we assume that a neural perturbation system is capable of generating random noise-like signals and that these input signals can be recorded simultaneously. We generated 32-channel time-series data using the matrix ***A*** shown in Appendix 2—figure 4a. Data were generated at sampling rate $f_{s} = 100$ Hz, with total length T=10,000 samples. The system matrix $\boldsymbol {A}\in \mathbb{R}^{32\times 32}$ was constructed as the block diagonal of 16 discrete-time 2×2 rotation blocks whose eigenvalue radii were drawn from $\mathrm{Unif}(0.9,\,0.95)$ and modal frequencies from $\mathrm{Unif}(1,\,100)$ Hz. The system dynamics exhibit multiple modes, as shown in Appendix 2—figure 4b. The input matrix was $\boldsymbol {B}= \boldsymbol {I}_{32}$ with single-node stimulation applied through its first column. Process noise was i.i.d. Gaussian with per-component standard deviation $\sigma _{\xi } = 10^{-2}$. The designed input was a sinusoidal input with frequencies matching the modal frequencies of the system, which were extracted from the eigenvalues of ***A***. Starting from white noise, the modal frequencies $\{\omega _{k}\}$ extracted from the eigenvalues of $\hat {\boldsymbol {A}}$ were used as the component frequencies of the next input (design-1), and this redesign was iterated twice more (design-2, design-3). To ensure a fair comparison, both the white noise and the designed inputs were normalized to have the same total input power. This setup allows for a controlled comparison between white noise input and frequency-optimized input.

The system identification error decreased when using frequency-designed inputs rather than white noise. Appendix 2—figure 4c shows the sum of reciprocal eigenvalues ($\sum _{i} (1/\mu _{i})$) of the covariance matrix of the state vectors. This value decreased when using frequency-designed inputs rather than white noise, indicating improved accuracy in system identification. Moreover, iterative redesigns progressively reduced the sum of reciprocal eigenvalues (designs 2 and 3), leading to more accurate identification. As shown in Appendix 2—figure 4d, both white noise and designed concatenated frequency inputs excited all dynamical modes without collapsing the smaller axes. The ellipsoids obtained with the designed input had smaller volumes, indicating higher identification accuracy. Therefore, this simulation demonstrates that iterative optimal design can yield more accurate system identification than conventional white noise stimulation.

In summary, white noise input offers a simple and practical strategy for neural system identification when no prior dynamical information is available. Nevertheless, it remains suboptimal compared with concatenated frequency input, which provides higher accuracy and stability through iterative refinement. These findings also highlight the importance of input optimization in achieving precise system identification in complex neural systems.
