# Supplementary material for: Designing optimal perturbation inputs for system identification in neuroscience
Source: eLife. 2026 Oct 13;15:RP110030. doi: 10.7554/eLife.110030 (PMC13719165; doi:10.7554/eLife.110030)
Supplement: Supplementary file 1 [file elife-110030-mdarchecklist1.docx]

**Materials Design Analysis Reporting (MDAR)**

**Checklist for Authors**

The [MDAR framework](https://osf.io/xfpn4/) establishes a minimum set of requirements in transparent reporting mainly applicable to studies in the life sciences.

*eLife* asks authors to **provide detailed information within their article** to facilitate the interpretation and replication of their work. Authors can also upload supporting materials to comply with relevant reporting guidelines for health-related research (see [EQUATOR Network](http://www.equator-network.org/%20)), life science research (see the [BioSharing Information Resource](http://biosharing.org/)), or animal research (see the [ARRIVE Guidelines](http://www.plosbiology.org/article/info:doi/10.1371/journal.pbio.1000412) and the [STRANGE Framework](https://doi.org/10.1038/d41586-020-01751-5); for details, see *eLife*’s [Journal Policies](https://reviewer.elifesciences.org/author-guide/journal-policies)). Where applicable, authors should refer to any relevant reporting standards materials in this form.

For all that apply, please note **where in the article** the information is provided. Please note that we also collect information about data availability and ethics in the submission form.

**Materials:**

| **Newly created materials** | **Indicate where provided: section/figure legend** | **N/A** |
| --- | --- | --- |
| The manuscript includes a dedicated "materials availability statement" providing transparent disclosure about availability of newly created materials including details on how materials can be accessed and describing any restrictions on access. | This is an entirely computational (numerical simulation) study. No new physical materials - reagents, constructs, cell lines, or organisms - were created, so no materials availability statement is required. Availability of the simulation code is stated in the Data Availability section. | **X** |
|  |  |  |
| **Antibodies** | **Indicate where provided: section/figure legend** | **N/A** |
| For commercial reagents, provide supplier name, catalogue number and [RRID](https://scicrunch.org/resources), if available. | No antibodies or other commercial reagents were used. | **X** |
|  |  |  |
| **DNA and RNA sequences** | **Indicate where provided: section/figure legend** | **N/A** |
| Short novel DNA or RNA including primers, probes: Sequences should be included or deposited in a public repository. | No DNA or RNA sequences were generated or used. | **X** |
|  |  |  |
| **Cell materials** | **Indicate where provided: section/figure legend** | **N/A** |
| Cell lines: Provide species information, strain. Provide accession number in repository OR supplier name, catalog number, clone number, OR RRID. | No cell lines were used. | **X** |
| Primary cultures: Provide species, strain, sex of origin, genetic modification status. | No primary cultures were used. | **X** |
|  |  |  |
| **Experimental animals** | **Indicate where provided: section/figure legend** | **N/A** |
| Laboratory animals or Model organisms: Provide species, strain, sex, age, genetic modification status. Provide accession number in repository OR supplier name, catalog number, clone number, OR RRID. | No laboratory animals or model organisms were used. | **X** |
| Animal observed in or captured from the field: Provide species, sex, and age where possible. | No animals were observed in or captured from the field. | **X** |
|  |  |  |
| **Plants and microbes** | **Indicate where provided: section/figure legend** | **N/A** |
| Plants: provide species and strain, ecotype and cultivar where relevant, unique accession number if available, and source (including location for collected wild specimens). | No plants were used. | **X** |
| Microbes: provide species and strain, unique accession number if available, and source. | No microbes were used. | **X** |
|  |  |  |
| **Human research participants** | **Indicate where provided: section/figure legend) or state if these demographics were not collected** | **N/A** |
| If collected and within the bounds of privacy constraints report on age, sex, gender and ethnicity for all study participants. | No human research participants were involved; no demographic data were collected. | **X** |

**Design:**

| **Study protocol** | **Indicate where provided: section/figure legend** | **N/A** |
| --- | --- | --- |
| If the study protocol has been pre-registered, provide DOI. For clinical trials, provide the trial registration number OR cite DOI. | The study was not pre-registered and is not a clinical trial. | **X** |
|  |  |  |
| **Laboratory protocol** | **Indicate where provided: section/figure legend** | **N/A** |
| Provide DOI OR other citation details if detailed step-by-step protocols are available. | No wet-laboratory protocol applies. The complete simulation and estimation procedure is described in Methods, 'State Vector Simulation and Model Estimation', and every simulation parameter (sampling frequency, time length, data length, number of trials, noise level, input strength) is tabulated in Appendix 1, 'Simulation Details' (Table: Conditions for generating neural dynamics). | **X** |
|  |  |  |
| **Experimental study design (statistics details) *** | | |
| **For in vivo studies: State whether and how the following have been done** | **Indicate where provided: section/figure legend. If it could have been done, but was not, write “not done”** | **N/A** |
| Sample size determination | Not an in vivo study. The number of independent simulation trials for each figure is given in Appendix 1, 'Simulation Details' (Table: Conditions for generating neural dynamics) ('Number of Trials' column; 100 for Figure 2, 10,000 for Figures 4-5, 200 for Figure 6, 100 for Figures 7 and 9, 500 for Figure 10). Trial counts were chosen to give stable Monte Carlo averages, not by statistical power analysis. | **X** |
| Randomisation | Not an in vivo study; there are no experimental units to allocate to groups. Every trial independently draws a random initial condition x(0) and an independent process-noise realisation (Methods, 'State Vector Simulation and Model Estimation'). | **X** |
| Blinding | Not an in vivo study. Blinding is not applicable: outcomes are computed deterministically from simulated data by fixed estimation code, with no experimenter-dependent measurement or subjective scoring. | **X** |
| Inclusion/exclusion criteria | Not an in vivo study. No inclusion or exclusion criteria were applied; every simulated trial was included in the reported analyses. | **X** |
|  |  |  |
| **Sample definition and in-laboratory replication** | **Indicate where provided: section/figure legend** | **N/A** |
| State number of times the experiment was replicated in the laboratory. | Methods, 'State Vector Simulation and Model Estimation'; Appendix 1, 'Simulation Details' (Table: Conditions for generating neural dynamics). Each reported quantity is an average over the stated number of independent simulation trials, with variations in initial conditions and noise sequences. |  |
| Define whether data describe technical or biological replicates. | The technical/biological replicate distinction does not apply to numerical simulation. All replicates are independent simulation trials, each using a different random initial condition and a different process-noise realisation (Methods, 'State Vector Simulation and Model Estimation'). | **X** |
|  |  |  |
| **Ethics** | **Indicate where provided: section/submission form** | **N/A** |
| Studies involving human participants: State details of authority granting ethics approval (IRB or equivalent committee(s), provide reference number for approval. | No human participants were involved; no ethics approval was required. | **X** |
| Studies involving experimental animals: State details of authority granting ethics approval (IRB or equivalent committee(s), provide reference number for approval. | No experimental animals were involved; no ethics approval was required. | **X** |
| Studies involving specimen and field samples: State if relevant permits obtained, provide details of authority approving study; if none were required, explain why. | No specimens or field samples were collected; no permits were required. | **X** |
|  |  |  |
| **Dual Use Research of Concern (DURC)** | **Indicate where provided: section/submission form** | **N/A** |
| If study is subject to dual use research of concern regulations, state the authority granting approval and reference number for the regulatory approval. | The study is not subject to dual use research of concern regulations. | **X** |

**Analysis:**

| **Attrition** | **Indicate where provided: section/figure legend** | **N/A** |
| --- | --- | --- |
| Describe whether exclusion criteria were pre-established. Report if sample or data points were omitted from analysis. If yes, report if this was due to attrition or intentional exclusion and provide justification. | Methods, 'State Vector Simulation and Model Estimation'. No exclusion criteria were pre-established and none were applied: no simulated trials or data points were omitted from any analysis, so there is no attrition to report. |  |
|  |  |  |
| **Statistics** | **Indicate where provided: section/figure legend** | **N/A** |
| Describe statistical tests used and justify choice of tests. | Methods, 'State Vector Simulation and Model Estimation' - estimation error is quantified by the Frobenius norm of the difference between the estimated and the true system matrix, averaged across independent trials; the spread shown in the figures is computed across those same trials. Results, 'Neural State Classification', and Figure 7c-f - classification uses linear discriminant analysis with 10-fold cross-validation, reported as mean accuracy and confusion matrices, together with ROC analysis. No inferential hypothesis tests (t-test, ANOVA or equivalent) were performed. All comparisons between input conditions are descriptive Monte Carlo summaries over large numbers of independent simulation trials, where the number of trials is set by the analyst; p-values would therefore be arbitrarily small and are not informative. |  |
|  |  |  |
| **Data availability** | **Indicate where provided: section/submission form** | **N/A** |
| For newly created and reused datasets, the manuscript includes a data availability statement that provides details for access (or notes restrictions on access). | Data Availability section (main text) and the submission form: the study is based entirely on numerical simulation and did not generate new experimental data. There are no restrictions on access. |  |
| When newly created datasets are publicly available, provide accession number in repository OR DOI and licensing details where available. | No new experimental datasets were created. All analysed data are simulation outputs that are fully reproducible from the publicly available source code (see Code availability below). | **X** |
| If reused data is publicly available provide accession number in repository OR DOI, OR URL, OR citation. | No previously published or third-party datasets were reused. | **X** |
|  |  |  |
| **Code availability** | **Indicate where provided: section/figure legend** | **N/A** |
| For any computer code/software/mathematical algorithms essential for replicating the main findings of the study, whether newly generated or re-used, the manuscript includes a data availability statement that provides details for access or notes restrictions. | Data Availability section (main text): 'All source code used to generate the simulations and to produce every figure in this paper is publicly available at https://github.com/mikito-ogino/NeuroPerturbID'. |  |
| Where newly generated code is publicly available, provide accession number in repository, OR DOI OR URL and licensing details where available. State any restrictions on code availability or accessibility. | https://github.com/mikito-ogino/NeuroPerturbID - a public repository containing all simulation and figure-generation scripts. Licence: dual-licensed under the MIT License or the BSD 3-Clause License at the user's option (SPDX-License-Identifier: MIT OR BSD-3-Clause; see LICENSE, LICENSE-MIT and LICENSE-BSD-3-CLAUSE in the repository). There are no restrictions on access, accessibility or reuse. |  |
| If reused code is publicly available provide accession number in repository OR DOI OR URL, OR citation. | No previously published third-party code was reused. The analyses rely only on standard open-source Python libraries (NumPy, SciPy, scikit-learn, NetworkX, Matplotlib, pandas, joblib, tqdm). |  |

**Reporting:**

The MDAR framework recommends adoption of discipline-specific guidelines, established and endorsed through community initiatives.

| **Adherence to community standards** | **Indicate where provided: section/figure legend** | **N/A** |
| --- | --- | --- |
| State if relevant guidelines (e.g., ICMJE, MIBBI, ARRIVE, STRANGE) have been followed, and whether a checklist (e.g., CONSORT, PRISMA, ARRIVE) is provided with the manuscript. | No discipline-specific reporting guideline (ICMJE, MIBBI, ARRIVE, STRANGE, CONSORT or PRISMA) applies to this purely computational modelling study, and no such checklist is provided. This completed MDAR checklist is submitted with the manuscript. | **X** |

* We provide the following guidance regarding transparent reporting and statistics; we also refer authors to [Ten common statistical mistakes to watch out for when writing or reviewing a manuscript](https://doi.org/10.7554/eLife.48175).

**Sample-size estimation**

- You should state whether an appropriate sample size was computed when the study was being designed
- You should state the statistical method of sample size computation and any required assumptions
- If no explicit power analysis was used, you should describe how you decided what sample (replicate) size (number) to use

**Replicates**

- You should report how often each experiment was performed
- You should include a definition of biological versus technical replication
- The data obtained should be provided and sufficient information should be provided to indicate the number of independent biological and/or technical replicates
- If you encountered any outliers, you should describe how these were handled
- Criteria for exclusion/inclusion of data should be clearly stated
- High-throughput sequence data should be uploaded before submission, with a private link for reviewers provided (these are available from both GEO and ArrayExpress)

**Statistical reporting**

- Statistical analysis methods should be described and justified
- Raw data should be presented in figures whenever informative to do so (typically when N per group is less than 10)
- For each experiment, you should identify the statistical tests used, exact values of N, definitions of center, methods of multiple test correction, and dispersion and precision measures (e.g., mean, median, SD, SEM, confidence intervals; and, for the major substantive results, a measure of effect size (e.g., Pearson's r, Cohen's d)
- Report exact p-values wherever possible alongside the summary statistics and 95% confidence intervals. These should be reported for all key questions and not only when the p-value is less than 0.05.

**Group allocation**

- Indicate how samples were allocated into experimental groups (in the case of clinical studies, please specify allocation to treatment method); if randomization was used, please also state if restricted randomization was applied
- Indicate if masking was used during group allocation, data collection and/or data analysis
