# Supplementary material for: Designing optimal perturbation inputs for system identification in neuroscience
Source: eLife. 2026 Oct 13;15:RP110030. doi: 10.7554/eLife.110030 (PMC13719165; doi:10.7554/eLife.110030)
Supplement: Peer review history

# Editor report: eLife Assessment

**DOI:** 10.7554/eLife.110030.3.sa0

**Authors:** Peter Latham

**Affiliations:** University College London United Kingdom

**Keywords:** Compelling, Important

This **important** study introduces a method, based on active control, for determining the functional connectivity between neurons or groups of neurons. While the theory is derived for a linear model with a perfectly observed control signal, the authors present **compelling** evidence that their method will work even when the system is nonlinear and the control signal is not fully observed. Furthermore, they provide a practical recipe for doing so.

# Referee report: Joint Public Review

**DOI:** 10.7554/eLife.110030.3.sa1

Summary:

Inferring so-called "functional connectivity" between neurons or groups of neurons is important both for validating models and for inferring brain state, including in human patients. This study aims to enhance this inference process by using closed-loop perturbation-based approaches. To this end, the authors develop a framework based on linear dynamical models that minimizes the estimation error. Based on this framework, the authors provide a practical guide for applying it in realistic experiments. Modalities include non-invasive ones, such as fMRI, iEEG, and invasive ones, such as optogenetic perturbations combined with neuropixel probes or calcium imaging.

Strengths:

A main strength of this paper is the application and adaptation of an explicit error expression to system dynamics estimation from evoked neural responses, bringing a useful theoretical tool into computational neuroscience for, as far as we know, the first time. Importantly, while the analytical derivation assumes the neural dynamics is linear and the control signal is known, these assumptions do not appear to be essential: their method outperforms passive observation even when the true dynamics is nonlinear or the control input is not known perfectly. Moreover, the relative simplicity of the method makes its practical applications straightforward, as the authors illustrate in the context of brain state classification and neural control.

Besides being of practical importance, simply pointing out that passive observation can lead to large mis-estimation of functional connectivity should serve as a wakeup call to anybody engaged in this endeavor.

Weaknesses:

None.

# Author comment: Author response

**DOI:** 10.7554/eLife.110030.3.sa2

**Authors:** Mikito Ogino, Daiki Sekizawa, Jun Kitazono, Masafumi Oizumi

**Affiliations:** The University of Tokyo Tokyo Japan; The University of Tokyo Tokyo Japan; Yokohama City University Yokohama Japan; The University of Tokyo Tokyo Japan

The following is the authors’ response to the original reviews.

**Joint Public Review:**

Weaknesses:

(1) The derivation of the main error term misses some important steps, which complicates peer review at this stage. In particular, factorisation of the covariance into noise and the inverse of the observation covariance matrix needs a more thorough justification. The cited sources do not contain the derivation for a noise term with full covariance, which is essential for deriving this error term.

The derivation of the main error term misses some important steps, which complicates peer review at this stage

We thank the reviewers for this careful observation. This concern is associated with the error term. Thus, we first clarified the noise assumption explicitly. We assume that *ξ*(*t*) is i.i.d. over time with zero mean and an arbitrary (not necessarily diagonal) positive definite covariance matrix $\boldsymbol{\Sigma}_{\xi}=\mathbb{E}\left[\boldsymbol{\xi}(t) \boldsymbol{\xi}(t)^{\top}\right] \in \mathbb{R}^{n \times n} \succ 0$.

In particular, factorisation of the covariance into noise and the inverse of the observation covariance matrix needs a more thorough justification. The cited sources do not contain the derivation for a noise term with full covariance, which is essential for deriving this error term.

The cited sources do contain the derivation for a noise term with full covariance. We have updated the citation that directly supports Eq. (S.2): Proposition 11.1 of Hamilton (1994, TimeSeries Analysis), which establishes the asymptotic distribution $\sqrt{T} \operatorname{vec}\left(\hat{\mathbf{\Pi}}_{T}-\pi\right) \xrightarrow{L} \mathcal{N}\left(0, \Omega \otimes Q^{-1}\right)$ The proof, given in Appendix 11.A of Hamilton (1994), proceeds via a CLT for martingale difference sequences. See Theoretical Details of the Supplementary Materials.

(2) The practical recommendation at the end of the paper also requires clearer guidance on how the design perturbations are constructed, and how many times and for how long the system is stimulated in each iteration of the experiment.

Thank you for this helpful suggestion. We agree that the practical implementation of the experimental design should be explained more clearly. We have addressed this concern in two ways. First, we have revised the manuscript to explicitly describe the parameter design procedure. Second, we have revised the manuscript to clearly provide a reference to the detailed experimental condition table in the supplementary material. See Results - Main Manuscript.

(3) Finally, there is no analysis of model mis-specification. In particular, the true dynamics are unlikely to be linear; the noise is unlikely to be either Gaussian or uncorrelated across time; and the B matrix is unlikely to be known perfectly. We’re not suggesting that the authors consider a more complex model, but it’s important to know how sensitive their method is to model mismatch. If nothing can be done analytically, then simulations would at least provide some kind of guide.

We thank the reviewer for raising this important point regarding model mis-specification. We agree that it is important to run simulations to assess the impact of these model mismatches, therefore we conducted preliminary simulations to assess the sensitivity when two primary assumptions are violated: linear state dynamics and a perfectly known input matrix *B*. We added these preliminary results in the Supplementary Material, and revised the main manuscript to include a mention of these results. In summary, the simulations showed:

The model estimation error increases with the strength of the nonlinearity; however, perturbation can increase the information and lead to accurate estimation of the hidden mode even under nonlinearity.

The matrix *A* can be estimated roughly even if the assumed input matrix *B* differs from the true matrix in some cases.

The matrices *A* and *B* can be estimated jointly without bias, provided the stimulation pattern excites the full state space.

In such joint estimation, preferentially exciting the hidden modes directly leads to more accurate estimation of *A* than stimulating other modes. See Background and Results - Main Manuscript, Experimental Conditions and Results - Supplementary Material.

**Recommendations for the authors:**

(1) Please tell us what tACS, tDCS, and TMS are, and how much control the experimenter has over them. That’s important, because they are going to be used as control signals, so we need to know how accurately u(t) can be specified, and what its range is.

Please tell us what tACS, tDCS, and TMS are, and how much control the experimenter has over them.

We appreciate the reviewer’s helpful comment. We agree that it is important to describe these stimulation methods with appropriate references. We have added a new section titled “Neural Stimulation as Control Inputs” to the background, which connects our theoretical framework to practical experimental settings.

We need to know how accurately u(t) can be specified, and what its range is.

The accuracy and range of control inputs vary substantially depending on the specific stimulation technique and experimental setup, and a thorough discussion would require a dedicated review beyond the scope of this manuscript. Instead, we have added a sentence acknowledging the gap between practical experimental implementations and the theoretical formulation, and cited relevant references for readers interested in further details.

Background - Main Manuscript

“Neural Stimulation as Control Inputs

This section describes how commonly used neural stimulation techniques can be related to input signals in control theory. Their adjustable parameters vary depending on how the stimulation inputs are modulated.

Three non-invasive electrical stimulation methods illustrate how stimulation paradigms map onto basic control inputs. Transcranial magnetic stimulation (TMS) induces brief and transient perturbations via electromagnetic pulses [19], which are naturally represented as a sequence of impulse-like inputs, where the timing and intensity of each pulse are the primary controllable parameters. Transcranial direct current stimulation (tDCS) primarily modulates neural activity through approximately constant inputs [26], which can be viewed as a step-like signal whose main controllable parameter is the amplitude of the applied current. Transcranial alternating current stimulation (tACS) delivers oscillatory inputs [7], corresponding to sinusoidal signals characterized by amplitude, frequency, and phase. In control theory, impulse, step, and sinusoidal inputs are the basic components used to characterize system responses and dynamics [22, 21].

The control input framework extends beyond non-invasive techniques to invasive and optogenetic stimulation. Invasive electrical stimulation, including intracranial microstimulation and deep brain stimulation (DBS), enables direct delivery of electrical inputs to neural tissue [17], providing flexible control over amplitude and timing through pulse trains or temporally structured waveforms. Optogenetic stimulation allows genetically targeted activation or inhibition of specific neurons using light [5], providing fine-grained control over multiple input dimensions, including amplitude (light intensity), temporal pattern, and cell-type specificity. In particular, recent developments enable stimulation at the level of individual neurons with high temporal precision [27, 16], allowing flexible construction of spatiotemporal input patterns.

These stimulation examples demonstrate that the theoretical framework developed in this paper connects to practical experimental settings. While a substantial gap remains between idealized control inputs in theory and experimentally realizable stimulation, the core principles established in the following sections provide a foundation that naturally extends to these practical stimulation paradigms.”

(2) Is the solid curve in the last panel of Figure 4b the prediction? If not, are the data points consistent with the prediction in Equation 8? This should be clear.

Thank you for this question. The solid curve shows the empirical eigenvalues of the state covariance matrix, not the eigenvalues of matrix *A*. As shown in Equation 9, the estimation error is proportional to the inverse of the sum of the eigenvalues of the state covariance matrix. We have clarified these points by revising the main text and the figure captions. See Results - Main Manuscript.

(3) Page 10, "Nodes 7 and 8 have only outgoing edges". It looks like node 7 has an incoming edge from node 8 (Fig. 6b). Or are we misinterpreting something?

Thank you for pointing out this inconsistency. You are correct. Node 7 did have an incoming edge from Node 8 and contradicted the statement in the text. Moreover, we now think that two hub nodes (Node 7 and 8) are not necessary for demonstrating our primary theory. To resolve these issues, we have revised the simulation so that the network now contains a single hub node with only outgoing edges. Please refer to Fig. 6.

(4) Figure 6f, g: why isn't the estimation error proportional to tr[Sig_x^{-1}]?

We thank the reviewer for this observation. In the original manuscript, the estimation error in Figure 6f, g was plotted on a logarithmic scale, which obscured the proportional relationship with $\operatorname{tr}\left[\boldsymbol{\Sigma}_{\boldsymbol{X}}^{-1}\right]$. The underlying values are indeed proportional, consistent with our theoretical prediction.

In the revised manuscript, we have substantially reorganized Figure 6 to make the theoretical reasoning more transparent by using 1/*µ* instead of $\operatorname{tr}\left[\boldsymbol{\Sigma}_{\boldsymbol{X}}^{-1}\right]$ following Eq. 9. The sum across the column in panel (g) is proportional to the estimation error shown in panel (h).

(5) Page 11, "The simulation was conducted with a single node receiving an impulse-shaped perturbation input." What’s an "impulse-shaped perturbation input"? A delta function? Please make this clear.

Thank you for this clarifying question. We clarified the explanation as follows.

Results - Main Manuscript

“Each node was individually perturbed by an impulse input. Here, an impulse input is defined as a Kronecker delta at t = 0 with fixed amplitude *α* = 10, with no external input applied at any subsequent time step.”

(6) Page 11, "Fig. 6d shows that the system possesses modes with small absolute eigenvalues." According to Figure 6d, all the absolute eigenvalues are between 9.9 and 9.98. So this statement appears not to be correct. Are we missing something?

Thank you for pointing this out. You are correct. The previous statement was inconsistent with the figure. In the revised simulation, we have redesigned the network so that it clearly contains modes with distinct damping characteristics: heavily damped modes with absolute eigenvalues below 0.8 and lightly damped modes with absolute eigenvalues close to 1.0 (see revised Fig. 6c). This makes the relationship between mode damping and perturbation effectiveness much more transparent.

Results - Main Manuscript

“Figs. 6c shows the damping rates |*λA*| of the eigenvalues of *A*: the mode formed by Nodes 1 and 2 (15Hz) is heavily damped, while those formed by Nodes 3–6 are moderately damped. Node 7 serves as a hub with only outgoing edges.”

(7) Page 11, "Crucially, the eigenvectors corresponding to these rapidly decaying modes (e.g., evec 7, evec 8) have their largest components concentrated at Nodes 7 and 8." If "nodes" are the same as "eigenvalue index", then the components on node 8 are zero (Figure 6e, bottom). In any case, it should be clear what you mean.

Thank you for this comment. We agree that the previous description regarding eigenvectors was unclear and inconsistent with the figure. We now think that explaining with eigenvectors is not necessary for demonstrating our primary theory. We have therefore replaced the plots of eigenvectors with the reciprocals of the eigenvalues of Σ_*X*_, which are directly and rigorously explained by Equation (9). These reciprocals clearly show that the modes associated with Nodes 1 and 2 are heavily damped and applying perturbations to these nodes contribute to the estimation error and the perturbations to hub node (Node 7) broadly increases all eigenvalues of Σ_*X*_, thereby reducing the estimation error across all modes. This change ensures that all simulation results are grounded in the theory presented in the paper. Please refer to the revised Fig. 6d–g for details.

(8) It’s not clear to us what’s plotted in Figure 6e. The real part of the eigenvectors? Which would explain why some of the eigenvectors are the same (e.g., 1 and 2). But that does not seem like a good idea, since the eigenvectors can be rotated by an arbitrary complex phase. Also, eigenvectors 7 and 8 seem totally opposite, and there’s no weight at all on index 6 and index 8. Could there be a mistake in the figure? In addition, Figure 6e is explained and interpreted in two different paragraphs, discussing the same observation. It would be easier to understand if they were moved to the same paragraph. Also, in the second paragraph where plot 6e is referenced (page 11, line 19), what does ’concentrated’ mean?

Thank you for this comment. As described in our response to Comment (7), we have removed the eigenvector-based analysis from the revised simulation to prevent confusing readers. The revised results focus on quantities that are directly explained by Equation (9). Please refer to the revised Fig. 6 for the updated results.

(9) Page 11, "This simulation demonstrates that, given a tentative connectivity matrix, an effective perturbation input (such as TMS or tDCS) can be designed by targeting the node with the highest weighted out-degree." "Demonstrates" seems strong. There is only one simulation, and that wasn’t totally convincing: a perturbation applied to node 7 did almost the same as a perturbation applied to nodes 1-6, even though it had a much higher outdegree than those nodes. It would be very helpful if you provided theoretical reasoning for why perturbing nodes with high-weight connections (Figure 6) minimises the prediction error. In particular, which properties of a hub node make it effective as a stimulation target? Why is it just its total output weights and not also its number of edges/centrality? How is the high output weight of a node related to its alignment with other eigenvectors, and is this always the case or just in this example?

Demonstrates seems strong.

Thank you for this important comment. We agree that "demonstrates" was too strong and have replaced it with "illustrates."

It would be very helpful if you provided theoretical reasoning for why perturbing nodes with high-weight connections (Figure 6) minimises the prediction error.

We agree with this comment. We have revised the simulation and accompanying text to connect the results directly to the main theory based on $\operatorname{tr}\left[\boldsymbol{\Sigma}_{\boldsymbol{X}}^{-1}\right]$. As responded to Comments (7) and (8), we have removed the eigenvector-based analysis and instead plotted the reciprocals of the eigenvalues of Σ_*X*_, which are directly related to the estimation error via Equation (9). This change allows us to provide a clear theoretical explanation for why perturbing certain nodes minimizes the prediction error.

Is this always the case or just in this example?

We have also explicitly stated the limitations: whether a hub node or a specific subnetwork node is more effective depends on factors such as the outgoing edge weights from the hub and the individual damping rates of each mode. The revised text emphasizes that this simulation presents one example of perturbation location design, and the optimal strategy must be evaluated case by case using the theoretical framework of Equation (9).

Results - Main Manuscript

“As this simulation represents only one example of location design, its limitations and the corresponding countermeasures should be stated. In actual experiments, the most effective perturbation location depends on factors such as hub-node connectivity and modal damping rates, and *B* itself may not always be known a priori. In such cases, approaches such as iterative optimization of $\operatorname{tr}\left[\boldsymbol{\Sigma}_{\boldsymbol{X}}^{-1}\right]$ (described in a later section) and joint estimation of *A* and *B* (Supplementary Material B.3) provide systematic alternatives. Nevertheless, the results presented here provide an intuitive guideline: stimulation directed at hub nodes or at nodes driving heavily damped modes effectively excites the full set of dynamical modes and minimizes estimation error.”

(10) What are the physical units for the time scales and stimulation amplitudes? For instance, on page 13, there is an argument that "In practical experiments, such long windows are unrealistic because neural states change rapidly over time." However, it is unclear whether T=100 a.u. or T=1000 a.u. etc. is realistic. By relating it to the eigenspectrum of A, which is supposed to be physiologically realistic, one can estimate the length of the stimulation window and support the above statement. Similarly, the impulse amplitude on page 13 is alpha=10^20^ (a.u.). Also, Table 1 in the Supplementary has an extremely wide range of stimulation amplitudes. Is 10^20^ a.u. a feasible amplitude in practice? And finally, please tell us which nodes the input was applied to.

Thank you for your incisive comments. We have addressed each comment as follows.

What are the physical units for the time scales and stimulation amplitudes?

They don’t have physical units. This study is a theoretical investigation that focuses on the relative differences between passive observation and perturbation-based approaches, rather than providing precise predictions for specific experimental settings. The time scales and stimulation amplitudes are therefore expressed in arbitrary units.

For instance, on page 13, there is an argument that "In practical experiments, such long windows are unrealistic because neural states change rapidly over time." However, it is unclear whether T=100 a.u. or T=1000 a.u. etc. is realistic.

We agree that the original expression “unrealistic” was not appropriate given the arbitrary units. We have revised the text to clarify that the time scales are in arbitrary units and that the main point is about the relative difference in required data length between passive observation and perturbation-based approaches, rather than making an absolute claim about feasibility.

Results - Main Manuscript

“To obtain estimates under the passive condition that are comparable to those derived under perturbation, it is necessary to experimentally observe extensive time-series data. Figure 7f illustrates the LDA projection and classification accuracy for different time-series lengths. The leftmost LDA plot (*T* = 20) corresponds to the passive condition shown in Fig. 7c, indicating that the estimation performance in the passive condition becomes comparable to that in the perturbation condition only when the time window reaches approximately *T* = 200, a 10-fold increase compared to *T* = 20. While the absolute duration depends on the interpretation of the time unit, such time windows may not be prohibitive in some experimental settings. Nevertheless, our results consistently show that passive observation requires substantially longer recordings to achieve comparable performance, highlighting the efficiency of the perturbation-based approach when the available data length is limited.”

Is 10^20^ a.u. a feasible amplitude in practice?

Although we have already stated that the stimulation amplitudes are in arbitrary units, we agree that the original value of 10^20^ was excessively large and could be misleading. We have revised the impulse amplitude from 10^20^ to 10^2^, as 10^20^ is physically unrealistic—it would imply a stimulus intensity many orders of magnitude beyond any conceivable experimental setting. The revised value of 10^2^ is more plausible: for reference, TMS stimulation voltages exceed typical EEG amplitudes by roughly 4–6 orders of magnitude. The classification accuracy decreased slightly; however, our main conclusion regarding the efficiency of the perturbation-based approach under limited data remains unchanged. See Table B.1.

Finally, please tell us which nodes the input was applied to.

The stimulus location was optimized to minimize the $\operatorname{tr}\left[\boldsymbol{\Sigma}_{\boldsymbol{X}}^{-1}\right]$. We have clarified this procedure in the main text and added a visual indication of the selected stimulation site (red circles) in Fig.7.

Results - Main Manuscript

“The neural signals were simulated under five different task conditions and two stimulation conditions: passive observation and external perturbation. Perturbation was applied as impulse-type inputs, such as TMS. The stimulus location was determined for each task condition by applying an impulse to each node and selecting the one that minimized $\operatorname{tr}\left[\boldsymbol{\Sigma}_{\boldsymbol{X}}^{-1}\right]$. The resulting time-series data are shown in Fig. 7b. Using this data, we estimated the underlying dynamical system via a control-based identification approach presented in Eq. 5, which corresponds to an estimation of functional connectivity.

(11) Page 13: "The controlled transition test was run with T = 1." Previously, T referred to the number of time steps. Is that the case here? If so, that seems hard to justify. If not, please tell us what T is (and, ideally, use a different symbol).

Is that the case here?

No. In this context, T does not denote the number of time steps.

If not, please tell us what T is (and, ideally, use a different symbol).

Thank you for your helpful comment. We have standardized the notation throughout the manuscript. In this paper, T consistently denotes the number of time steps (i.e., data length). In the sections “Neural State Classification” and “Neural State Transitions,” we had mistakenly used T to refer to time length. To resolve this inconsistency, we have added a separate column labeled “Data Length (T)” to Table B.1 for clarification and replaced the previous usage of T with “data length” where appropriate.

Results - Main Manuscript

“To obtain estimates under the passive condition that are comparable to those derived under perturbation, it is necessary to experimentally observe extensive time-series data. Figure 7f illustrates the LDA projection and classification accuracy for different time-series lengths. The leftmost LDA plot (*T* = 20) corresponds to the passive condition shown in Fig. 7c, indicating that the estimation performance in the passive condition becomes comparable to that in the perturbation condition only when the time window reaches approximately *T* = 200, a 10-fold increase compared to *T* = 20. While the absolute duration depends on the interpretation of the time unit, such time windows may not be prohibitive in some experimental settings. Nevertheless, our results consistently show that passive observation requires substantially longer recordings to achieve comparable performance, highlighting the efficiency of the perturbation-based approach when the available data length is limited.”

Results - Main Manuscript

“The controlled transition test was run with *T* = 50. The control objective was to set nodes3 and 4 to 25 while keeping all other nodes at 0 without any movement. See Table B.1.”

(12) Page 14: "where the matrix A (Fig. 10a) is designed to have 16 modes." What do you mean by has "16 nodes"?

Thank you for pointing this out. By “16 modes,” we refer to 16 dynamical eigenmodes (i.e., 16 eigenvalue pairs). In the real-valued state-space representation used in the simulations, each complex conjugate pair corresponds to a 2-dimensional real block, resulting in a 32-dimensional system (32 nodes). We revised the wording to clearly distinguish between the number of dynamical modes and the dimensionality (number of nodes) of the state vector, to avoid confusion.

Results - Main Manuscript

“Iterative refinement of both the perturbation design and the estimation process progressively improves the accuracy of *A*. The time-series data is collected from 32 points, where the matrix *A* (Fig. 10a) is designed to have 16 oscillatory modes (i.e., 16 complex-conjugate eigenvalue pairs, yielding 32 eigenvalues in total).”

(13) In Figure 10b, the y-axis should start at zero; otherwise, it’s a bit misleading how much the active perturbation helps. This will make it clear that the estimation error drops by about 33%. It would be worth commenting on whether this is typical; after all, potential users of this method would want to know how much improvement they’re likely to see.

It would be worth commenting on whether this is typical; after all, potential users of this method would want to know how much improvement they’re likely to see.

Thank you for this insightful comment. We agree with the reviewer that quantifying the expected improvement would be valuable for experimental practice. However, this simulation is a theoretical demonstration. Its primary purpose was to show that an iterative active perturbation approach can progressively converge to an optimal perturbation design even without prior knowledge of the true system, rather than to quantify a universally expected improvement rate.

The y-axis should start at zero; otherwise, it’s a bit misleading how much the active perturbation helps.

We believe that the y-axis should start at the accuracy with optimal perturbation because the primary purpose of this simulation was to demonstrate that an iterative active perturbation approach can progressively converge. If we had started the y-axis at zero, the message would be visually obscured.

Potential users of this method would want to know how much improvement they’re likely to see.

We acknowledge that this is one example of the application of our method, and the magnitude of improvement depends on various factors such as network structure, noise level, and stimulation design. We should not mislead the readers. We have therefore maintained the y-axis starting point and added a clarifying statement in the revised manuscript to indicate that the simulation is case-specific rather than universal.

Results - Main Manuscript

“These results should be interpreted as a case-specific illustration rather than a universal gain, as the magnitude of improvement depends on factors such as network structure, recording duration, noise level, and stimulation design. This simulation demonstrates that our perturbation design framework enables the step-by-step refinement of system identification even without prior knowledge of the system.”

(14) Please provide a derivation for the factorised covariance in Equation S.2. This is the equation that underpins the main result of the paper - the error in dynamical system estimation. Currently, it appears to be taken from [Hamilton, J. D. Time Series Analysis], yet we were unable to find this result in the book. Most of the derivations in Chapters 8.1 and 8.2 assume diagonal noise covariance, and even isotropic noise (cov = sigma*2 * I), which simplifies the particular case of the derivations. Could the authors provide a reference or the derivation for the case with full covariance?

As described in our response to the Weakness above, the relevant result is Proposition 11.1 of Hamilton (1994), not Chapters 8.1–8.2. Proposition 11.1 states the asymptotic distribution of the vectorised OLS estimator in a VAR model with i.i.d. innovations whose covariance matrix Ω is an arbitrary positive definite matrix. The factorisation $\boldsymbol{\Sigma}_{\mathrm{Asym}}=\boldsymbol{\Sigma}_{\xi} \otimes \boldsymbol{\Sigma}_{\boldsymbol{X}}^{-1}$ in our Eq. (S.2) corresponds directly to the revised manuscript we explicitly cite “Hamilton (1994), Proposition 11.1” at Eq. (S.1) and $\Omega \otimes Q^{-1}$ in Hamilton’s Proposition 11.1, with $\boldsymbol{\Sigma}_{\xi} \leftrightarrow \Omega$ and *ξ*(*t*) in the preceding paragraph, so that the connection to this result is unambiguous. See Theoretical Details Supplementary.$\boldsymbol{\Sigma}_{\boldsymbol{X}} \leftrightarrow Q$. In state the i.i.d. assumption on

(15) Strongly perturbing/Exciting fast-decaying modes to give them more ’runway’ and increase observed variability makes intuitive sense for a normal system. But what would happen in the case of non-normal dynamics, where stimulating one dimension only transiently amplifies it, but then excites other dimensions? Non-normality breaks the alignment between PC components and dynamic modes (see Kumar, Ankit, Loren M. Frank, and Kristofer E. Bouchard. "Identifying feedforward and feedback controllable subspaces of neural population dynamics." arXiv preprint arXiv:2408.05875 (2024)), so eigenvectors of A and Σ_*X*_ won’t align for non-normal dynamics. This alignment appears to be a hidden assumption of this paper. Should the normal dynamics then be stated as an assumption/limitation of the framework? Is the example in Figure 6a highly non-normal? Does considering out-degree provide an empirical approach, an alternative to ’enlargement’, to dealing with non-normality?

We thank the reviewer for this insightful comment regarding non-normal dynamics.

Should normal dynamics be stated as an assumption/limitation of the framework?

No. Our framework does not assume normal dynamics. For example, Figure 5 and the revised Figure 6 illustrate non-normal cases. In Figure 5, the row and column norms of *A* differ substantially (row norms ≈ [1.96, 4.05, 1.33, 4.70]; column norms ≈ [6.14, 1.94, 1.39, 0.87]). In Figure 6, Node 7 is a hub with only outgoing edges, so row 7 of *A* is zero while column 7 has nonzero entries. In addition, the Nodes 5–6 and Nodes 3–4 are strictly one-way, leaving the corresponding off-diagonal block upper-triangular. Both features break the symmetry required for normality.

We additionally computed a commutator-based non-normality index $\left\|\boldsymbol{A} \boldsymbol{A}^{\top}-\boldsymbol{A}^{\top} \boldsymbol{A}\right\|_{F} /\|\boldsymbol{A}\|_{F}^{2}$ which equals 0 for any normal matrix and approaches $\sqrt{2} \approx 1.41$ for the canonical maximally non-normal example (the 2×2 nilpotent Jordan block). The non-normality index is 1.34 for Figure 5 and 0.41 for Figure 6. These values confirm that both are clearly non-normal.

Is the example in Figure 6a highly non-normal?

Yes. As stated above, the network structure of Figure 6a clearly indicates non-normality.

Does considering out-degree provide an empirical approach, an alternative to ‘enlargement’, to dealing with non-normality?

No. In the previous manuscript, the results of out-degree and eigenvector structure were provided as supplementary intuition. However, the central contribution of our framework lies in maximizing the minimum eigenvalue *µ* of the observed state covariance (Equation 9), and for systems where non-normality is strong and subnetwork structure is less modular, the iterative optimization of $\operatorname{tr}\left[\boldsymbol{\Sigma}_{\boldsymbol{X}}^{-1}\right]$ provides a principled, assumption-free method. We clearly mentioned this point in the revised manuscript as follows. See Results in the main manuscript.

(16) In the iterative experiment at the very end of the paper (Figure 10), what was the strategy for designing ‘u_design_´? How many stimulations were applied in an iteration? Are you stimulating along eigenvectors? Do you sample from components randomly, or perturb each of them individually, with the amplitude proportional to reciprocals?

Thank you for this important question. We have clarified the design rule and stimulation protocol in the revised manuscript, and address each sub-question below.

How many stimulations were applied in an iteration?

One stimulation session was applied in an iteration.

Are you stimulating along eigenvectors?

The optimal stimulation is designed as the target node of the perturbation is determined through numerical optimization that minimizes $\operatorname{tr}\left[\boldsymbol{\Sigma}_{\boldsymbol{X}}^{-1}\right]$

Do you sample from components randomly, or perturb each of them individually, with the amplitude proportional to reciprocals?

The optimal stimulation is designed as a composite-frequency sinusoidal input encompassing all modes of the estimated Â, and the target node of the perturbation is determined through numerical optimization that minimizes $\operatorname{tr}\left[\boldsymbol{\Sigma}_{\boldsymbol{X}}^{-1}\right]$. See Results - Main Manuscript.

(17) While it is clear that the proposed active method performs better than passive observation, some results lack a comparison with stimulating random directions/nodes with a comparable control energy (Figure 7e & Figure 10b).

Thank you for your helpful comment. Although the optimally designed stimulation outperforms random stimulation, the previous stimulation settings were not configured to explicitly demonstrate this difference. Therefore, we modified the network structure, recording length, and stimulation intensity so that both the main messages and the difference from random stimulation can be shown simultaneously. Accordingly, we have added a comparison with random stimulation in both Figure 7e and Figure 10b.

In Figure 7e, we included a random stimulation condition where the target node is selected randomly, and the results show that the optimized stimulation outperforms random stimulation.

These additions strengthen the evidence for the effectiveness of our proposed method compared to non-optimized approaches.

(18) Is it reasonable to assume full observability of the system? It would be interesting to consider biases arising from the partial observability of the system, in the spirit of Figure 9, which looked at partial controllability.

We thank the reviewer for this suggestion. We agree that partial observability is an important consideration, however it is out of scope for the current work. Thus, we have added a future direction in the Discussion addressing partial observability. We note that Takens’ embedding theorem and Hankel DMD enable recovery of a system’s eigenvalues from partial observations, and since our framework relies on the eigenvalue structure of A, the proposed perturbation design remains applicable under partial observability.

Discussion - Main Manuscript

“Two directions warrant further investigation: extending the framework to partial observability, and validating it through stimulation experiments. In experimental neuroscience, recordings are often limited to a subset of neural populations, resulting in partial observability. A growing body of work has leveraged delay-embedding techniques, represented by Takens’ embedding theorem [25], to reconstruct hidden dynamics from partial observations [2, 3, 23, 11]. Applying such techniques enables the estimation of the full connectivity matrix, thereby extending our framework to settings with partial observability. The second direction concerns experimental validation. Validating a theoretical framework through experimental design is an essential in bridging the gap between theory and practice.”

Recommendations for improving the writing and presentation.

(1) The word ’state’ is overloaded in Figure 7. When talking about neural state classification, the ’state’ refers to a regime guided by a distinct dynamics A (should it be *A_i_*? Figure 7A bottom). However, each dynamical system also has a ’state’. A different word should be used in Figure 7A and the corresponding text.

We agree that the terminology is potentially confusing. To avoid the confusion, we replaced the term “state” with “task condition” and “neural signal” in the main text, and revised Fig. 7.

Results - Main Manuscript

“We designed a neural network with clearly distinct task conditions and considered a simulation setting in which these conditions are classified using signals of a fixed duration. These distinct task conditions consist of five types, each defined by a unique linear dynamical system characterized by differing eigenvalue spectra and connectivity topologies of matrix *A* (Fig. 7a). These task conditions are intended to mimic different cognitive or behavioral contexts. For example, in a typical motor task experiment, such conditions could correspond to motor execution or imagery involving the left or right hand, or resting state [1, 24]. The neural signal was simulated under five different task conditions and two stimulation conditions: passive observation and external perturbation. Perturbation was applied as impulse-type inputs, such as TMS. The stimulus location was determined for each task condition by applying an impulse to each node and selecting the one that minimized $\operatorname{tr}\left[\boldsymbol{\Sigma}_{\boldsymbol{X}}^{-1}\right]$. The resulting time-series data are shown in Fig. 7b. Using this data, we estimated the underlying dynamical system via a control-based identification approach presented in Eq. 5, which corresponds to an estimation of functional connectivity.”

(2) It would be helpful to provide dimensions of matrices around Equation S.2, since vectorization makes dimensions hard to track.

We thank the reviewer for this helpful suggestion. We agree that explicitly stating the matrix dimensions improves readability, particularly around the Kronecker product where vectorization can obscure the size of the resulting covariance matrix. We have revised the text as follows (the equation S.2 is 3 now). See Theoretical Details of the Supplementary Materials.

(3) The background sections of the paper would benefit from referring to similar active perturbation methods: Wagenmaker, Andrew, et al. "Active learning of neural population dynamics using two-photon holographic optogenetics." Advances in Neural Information Processing Systems 37 (2024): 31659-31687. Minai, Yuki, et al. "MiSO: Optimizing brain stimulation to create neural activity states." Advances in Neural Information Processing Systems 37 (2024): 24126-24149.

We thank the reviewer for these helpful suggestions. We have incorporated Wagenmaker et al. (2024) and Minai et al. (2024) into the Introduction. Their works focus on developing algorithmic approaches to active stimulation design for specific experimental platforms, while our work aims to establish a general theoretical framework for why and which perturbation inputs are effective has yet to be established. We cited these works and have clarified this distinction in the revised manuscript as follows.

Introduction - Main Manuscript

“In this paper, we propose a framework for designing the optimal perturbation input through control theory in neuroscience. We interpret neural dynamics as a control system [8, 6, 14, 12, 20, 24], and treat external perturbations as control inputs to design properties of neural stimulation (Fig. 1d). If the optimal perturbation input can be systematically designed, it becomes possible to steer the neural system toward states that are maximally informative (Fig. 1e), thereby enhancing the accuracy of the inferred connectivity (Fig. 1f). While recent studies have begun to develop algorithmic approaches to active stimulation design for specific experimental platforms [18, 28], a general theoretical framework for why and which perturbation inputs are effective has yet to be established. We first describe how to formulate neural dynamics as a control system and how to estimate the model parameters from observed data. Building upon this formulation, we derive a theoretical basis that enables us to design the optimal perturbation inputs for the neural system identification. We demonstrate the validity and utility of this theoretical basis by exploring its implications for optimizing parameters of common neurostimulation techniques and by applying it to practical examples, including neural state classification [4, 9, 1, 24] and control of neural states [8, 13, 14, 12]. In these demonstrations, we define concrete problems and apply the theory to validate its practical utility.”

Minor corrections to the text and figures.

(1) In Figure 3, it would be helpful to point out that the plots are in the subspace spanned by the first three principal components.

Thank you for pointing out. We revised the caption of the Fig.3 as follows. See Results - Main Manuscript.

(2) Figure 4 caption: "eigenvectors" –> "eigenvectors of Σ_X_", just to make it crystal clear (since A also has eigenvectors).

Thank you for your suggestion. We have revised the caption of Figure 4. See Results - Main Manuscript.

(3) Page 5, Equation (8): Capital xi should be introduced in the main text of the paper as the covariance matrix for the noise term xi. Now it can only be understood after reading the Supplementary. Or maybe call the covariance matrix Σ_*ξ*_rather than Σ_*ξ*_? That will probably make it clearer.

Thank you for this suggestion. We have made both changes. First, we introduced the noise covariance matrix explicitly in the main text immediately after Eq. (1), defining. Second, we replaced the notation Σ_*ξ*_ with Σ_*ξ*_ lowercase subscript matching the noise variable $\Sigma_{\xi}=\mathbb{E}\left[\xi(t) \xi(t)^{T}\right]\gt0$ throughout the main text and supplementary.

(4) Page 7, Equation (14): The description of the equation states "The covariance matrices of x(t) for impulse inputs can be written as:... ". We assume this is supposed to be the "state vector," not "covariance matrices".

Thank you for catching this. We have corrected the wording to “state vector” instead of “covariance matrices". See Results - Main Manuscript

(5) Page 10, after introducing Figures 6a-b, potentially a sentence is missing (’...’ in the first line of the last paragraph).

Thank you for pointing this out. We have removed the placeholder along with updating the stimulation settings for Figure 6.

(6) Page 10, Figure 6 (e): A clearer labelling would be helpful, e.g., a title for the legend (e.g. node index) and a more informative title (e.g. Eigenvector alignment with nodes), a caption (what is the take-home message?), and y-axis labels (what is the ’value’?).

Thank you for this helpful suggestion. We have revised the Figure 6 taking your suggestion into account. The new figure includes a clearer title, axis labels, and an informative caption that highlights the key take-home message. See Results - Main Manuscript

(7) Page 11, paragraph 1: The sentence "...(as discussed in Section)" is missing a section reference.

Thank you for pointing this out. We have replaced the section reference placeholder with the equation reference to Equation (9), which is the relevant theoretical result.

(8) Page 13, caption of Figure 8c: compputed -> computed.

We have corrected this typo. We also reviewed the manuscript for any similar typographical errors and corrected them.

(9) Page 14 Figure 9: The y-axis label for "Controlled State Process" plots is missing.

Thank you for catching this. The y-axis was hidden by other elements in the figure. We have revised the figure layout to ensure that the y-axis label is visible.

(10) Page 16: The sentence "As described in Section, a preliminary ... " is missing a section reference

Thank you for pointing this out. We have corrected the missing reference. The sentence now reads “as shown in Fig. 10” rather than the incomplete “as described in Section.”

(11) Page 16, Fig. 10c: It looks like the errors have inconsistent color ranges. A shared colorbar would help.

Thank you for your suggestion. We have revised Figure 10c to use a shared colorbar across all subplots.

(12) Page 17, Paragraph preceding eq. 16: "the eigenvectors of X" --> "the eigenvectors of Sigma_X".

Thank you for catching this. We have revised the text. See Methods - Main Manuscript.

(13) S.1: This is a GLS, not an OLS estimator, if this assumes full noise covariance.

As stated in our response to the Weakness above, we assume the noise term *ξ*(*t*) is i.i.d. with covariance matrix Σ_*ξ*_, and the estimator we analyze is the OLS estimator. See Theoretical Details - Supplementary Material.

(14) S.2: Unclear that⊗is the Kronecker product (not outer), as it is not defined.

We thank the reviewer for pointing out this ambiguity. In the revised Supplementary Material, we have explicitly explained the Kronecker product with a reference to Hamilton [10, Appendix A.4, p. 732]. See Theoretical Details - Supplementary Material.

(15) S.51: There is an accidental comma between alpha and A after "xdiff(t) = "

Thank you for pointing this out. We have removed the accidental comma. See Theoretical Details - Supplementary Material.

(16) Section B.2: It would be useful to have the simulation details for that section (as is given for the other section in B.1).

We thank the reviewer for this helpful suggestion. We have added a dedicated “Simulation details” paragraph to Appendix B.5 (the section containing Fig. B.4) so that the setup is now described with the same level of specificity as the other appendix sections. See Experimental Conditions and Results - Supplementary Material.

References

(1) Irma N Angulo-Sherman, Marisol Rodríguez-Ugarte, Nadia Sciacca, Eduardo Iáñez, and José M Azorín. Effect of tDCS stimulation of motor cortex and cerebellum on EEG classification of motor imagery and sensorimotor band power. J. Neuroeng. Rehabil., 14(1):31, April 2017.

(2) Hassan Arbabi and I Mezić. Computation of transient koopman spectrum using hankeldynamic mode decompoisition. APS, page G1.009, November 2017.

(3) Steven L Brunton, Bingni W Brunton, Joshua L Proctor, Eurika Kaiser, and J Nathan Kutz. Chaos as an intermittently forced linear system. Nat. Commun., 8(1):19, May 2017.

(4) Adenauer G Casali, Olivia Gosseries, Mario Rosanova, Mélanie Boly, Simone Sarasso, Karina R Casali, Silvia Casarotto, Marie-Aurélie Bruno, Steven Laureys, Giulio Tononi, and Marcello Massimini. A theoretically based index of consciousness independent of sensory processing and behavior. Sci. Transl. Med., 5(198), August 2013.

(5) Karl Deisseroth. Optogenetics. Nat. Methods, 8(1):26–29, January 2011.

(6) Shikuang Deng, Jingwei Li, B T Thomas Yeo, and Shi Gu. Control theory illustrates the energy efficiency in the dynamic reconfiguration of functional connectivity. Commun. Biol., 5(1):295, April 2022.

(7) Shrey Grover, Renata Fayzullina, Breanna M Bullard, Victoria Levina, and Robert M G Reinhart. A meta-analysis suggests that tACS improves cognition in healthy, aging, and psychiatric populations. Sci. Transl. Med., 15(697):eabo2044, May 2023.

(8) Shi Gu, Fabio Pasqualetti, Matthew Cieslak, Qawi K Telesford, Alfred B Yu, Ari E Kahn, John D Medaglia, Jean M Vettel, Michael B Miller, Scott T Grafton, and Danielle S Bassett. Controllability of structural brain networks. Nat. Commun., 6:8414, October 2015.

(9) Mark Hallett, Riccardo Di Iorio, Paolo Maria Rossini, Jung E Park, Robert Chen, Pablo Celnik, Antonio P Strafella, Hideyuki Matsumoto, and Yoshikazu Ugawa. Contribution of transcranial magnetic stimulation to assessment of brain connectivity and networks. Clin. Neurophysiol., 128(11):2125–2139, November 2017.

(10) James Douglas Hamilton. Time Series Analysis. Princeton University Press, Princeton, 1994.

(11) Ann Huang, Mitchell Ostrow, Satpreet H Singh, Leo Kozachkov, Ila Fiete, and Kanaka Rajan. InputDSA: Demixing then comparing recurrent and externally driven dynamics. arXiv [q-bio.NC], November 2025.

(12) Shunsuke Kamiya, Genji Kawakita, Shuntaro Sasai, Jun Kitazono, and Masafumi Oizumi. Optimal control costs of brain state transitions in linear stochastic systems. J. Neurosci., 43(2):270–281, January 2023.

(13) Teresa M Karrer, Jason Z Kim, Jennifer Stiso, Ari E Kahn, Fabio Pasqualetti, Ute Habel, and Danielle S Bassett. A practical guide to methodological considerations in the controllability of structural brain networks. J. Neural Eng., 17(2):026031, April 2020.

(14) Genji Kawakita, Shunsuke Kamiya, Shuntaro Sasai, Jun Kitazono, and Masafumi Oizumi. Quantifying brain state transition cost via schrödinger bridge. Netw. Neurosci., 6(1):118– 134, February 2022.

(15) Hassan K Khalil. Nonlinear systems. Prentice-Hall, Upper Saddle River, NJ, 2002.

(16) Paul K LaFosse, Zhishang Zhou, Jonathan F O’Rawe, Nina G Friedman, Victoria M Scott, Yanting Deng, and Mark H Histed. Single-cell optogenetics reveals attenuationby-suppression in visual cortical neurons. bioRxivorg, page 2023.09.13.557650, May 2024.

(17) Andres M Lozano, Nir Lipsman, Hagai Bergman, Peter Brown, Stephan Chabardes, Jin Woo Chang, Keith Matthews, Cameron C McIntyre, Thomas E Schlaepfer, Michael Schulder, Yasin Temel, Jens Volkmann, and Joachim K Krauss. Deep brain stimulation: current challenges and future directions. Nat. Rev. Neurol., 15(3):148–160, March 2019.

(18) Yuki Minai, Matthew Smith, Joana Soldado-Magraner, and Byron Yu. MiSO: Optimizing brain stimulation to create neural activity states. In A Globerson, L Mackey, D Belgrave, A Fan, U Paquet, J Tomczak, and C Zhang, editors, Advances in Neural Information Processing Systems 37, volume 37, pages 24126–24149, San Diego, California, USA, 2024. Neural Information Processing Systems Foundation, Inc (NeurIPS).

(19) Davide Momi, Zheng Wang, and John D Griffiths. TMS-evoked responses are driven by recurrent large-scale network dynamics. Elife, 12(e83232), April 2023.

(20) Ali Moradi Amani, Amirhessam Tahmassebi, Andreas Stadlbauer, Uwe Meyer-Baese, Vincent Noblet, Frederic Blanc, Hagen Malberg, and Anke Meyer-Baese. Controllability of functional and structural brain networks. Complexity, 2024(1), January 2024.

(21) Norman S Nise. Control Systems Engineering. John Wiley & Sons, 8 edition, 2020.

(22) Katsuhiko Ogata. Modern Control Engineering. Prentice Hall, 2010.

(23) Mitchell Ostrow, Adam Eisen, and Ila Fiete. Delay embedding theory of neural sequence models. arXiv [cs.LG], June 2024.

(24) Yumi Shikauchi, Mitsuaki Takemi, Leo Tomasevic, Jun Kitazono, Hartwig R Siebner, and Masafumi Oizumi. Quantifying state-dependent control properties of brain dynamics from perturbation responses. J. Neurosci., page e0364252025, December 2025.

(25) Floris Takens. Detecting strange attractors in turbulence. In David Rand and Lai-SangYoung, editors, Dynamical Systems and Turbulence, Warwick 1980, volume 898 of Lecture Notes in Mathematics, pages 366–381. Springer, Berlin, Heidelberg, 1981.

(26) Liam C Tapsell, Matheus D Pinto, Ann-Maree Vallence, Casey Whife, Maria Luciana Perez Armendariz, Shaswat Senger, Jack Andringa-Bate, Dana Hince, and Myles C Murphy. What are the optimal transcranial direct current stimulation parameters and design elements to modulate corticospinal excitability? a systematic review and longitudinal meta-analysis. Neurol. Res. Pract., 7(1):86, November 2025.

(27) Lei Tong, Shanshan Han, Yao Xue, Minggang Chen, Fuyi Chen, Wei Ke, Yousheng Shu, Ning Ding, Joerg Bewersdorf, Z Jimmy Zhou, Peng Yuan, and Jaime Grutzendler. Single cell in vivo optogenetic stimulation by two-photon excitation fluorescence transfer. iScience, 26(10):107857, October 2023.

(28) Andrew Wagenmaker, Lu Mi, Marton Rozsa, Matthew S Bull, Karel Svoboda, Kayvon Daie, Matthew D Golub, and Kevin Jamieson. Active learning of neural population dynamics using two-photon holographic optogenetics. Adv. Neural Inf. Process. Syst., 37:31659–31687, 2024.
